# Multicomponent reaction-based synthesis and biological evaluation of tricyclic heterofused quinolines with multi-trypanosomatid activity

**DOI:** 10.1016/j.ejmech.2015.10.007

**Published:** 2015-11-13

**Authors:** Ornella Di Pietro, Esther Vicente-García, Martin C. Taylor, Diana Berenguer, Elisabet Viayna, Anna Lanzoni, Irene Sola, Helena Sayago, Cristina Riera, Roser Fisa, M. Victòria Clos, Belén Pérez, John M. Kelly, Rodolfo Lavilla, Diego Muñoz-Torrero

**Affiliations:** a Laboratori de Química Farmacèutica (Unitat Associada al CSIC), Facultat de Farmàcia, and Institut de Biomedicina (IBUB), Universitat de Barcelona, Av. Joan XXIII, 27-31, E-08028, Barcelona, Spain; b Barcelona Science Park, Baldiri Reixac, 10-12, E-08028, Barcelona, Spain; c Department of Pathogen Molecular Biology, London School of Hygiene and Tropical Medicine, Keppel Street, London WC1E 7HT, United Kingdom; d Laboratori de Parasitologia, Departament de Microbiologia i Parasitologia Sanitàries, Facultat de Farmàcia, Universitat de Barcelona, Av. Joan XXIII, 27-31, E-08028, Barcelona, Spain; e Departament de Farmacologia, de Terapèutica i de Toxicologia, Institut de Neurociències, Universitat Autònoma de Barcelona, E-08193, Bellaterra, Barcelona, Spain; f Laboratori de Química Orgànica, Facultat de Farmàcia, Universitat de Barcelona, Av. Joan XXIII, 27-31, E-08028, Barcelona, Spain

**Keywords:** Benzo[*h*][1,6]naphthyridines, Pyrano[3,2-*c*]quinolines, Povarov reaction, Trypanocidal agents, Leishmanicidal agents, Brain permeability

## Abstract

Human African trypanosomiasis (HAT), Chagas disease and leishmaniasis, which are caused by the trypanosomatids *Trypanosoma brucei*, *Trypanosoma cruzi* and *Leishmania* species, are among the most deadly neglected tropical diseases. The development of drugs that are active against several trypanosomatids is appealing from a clinical and economic viewpoint, and seems feasible, as these parasites share metabolic pathways and hence might be treatable by common drugs. From benzonapthyridine **1**, an inhibitor of acetylcholinesterase (AChE) for which we have found a remarkable trypanocidal activity, we have designed and synthesized novel benzo[*h*][1,6]naphthyridines, pyrrolo[3,2-*c*]quinolines, azepino[3,2-*c*]quinolines, and pyrano[3,2-*c*]quinolines through 2–4-step sequences featuring an initial multicomponent Povarov reaction as the key step. To assess the therapeutic potential of the novel compounds, we have evaluated their *in vitro* activity against *T. brucei*, *T. cruzi*, and *Leishmania infantum*, as well as their brain permeability, which is of specific interest for the treatment of late-stage HAT. To assess their potential toxicity, we have determined their cytotoxicity against rat myoblast L6 cells and their AChE inhibitory activity. Several tricyclic heterofused quinoline derivatives were found to display an interesting multi-trypanosomatid profile, with one-digit micromolar potencies against two of these parasites and two-digit micromolar potency against the other. Pyranoquinoline **39**, which displays IC_50_ values of 1.5 μM, 6.1 μM and 29.2 μM against *T. brucei*, *L. infantum* and *T. cruzi*, respectively, brain permeability, better drug-like properties (lower lipophilicity and molecular weight and higher CNS MPO desirability score) than hit **1**, and the lowest AChE inhibitory activity of the series (IC_50_ > 30 μM), emerges as an interesting multi-trypanosomatid lead, amenable to further optimization particularly in terms of its selectivity index over mammalian cells.

## Highlights

• Novel classes of tricyclic heterofused quinolines have been synthesized.
• Their 2–4-step syntheses involve a multicomponent Povarov reaction as the key step.
• Some compounds exhibit single digit micromolar potencies against 2 trypanosomatids.
• All compounds with multi-trypanosomatid activity can cross the blood–brain barrier.
• Most compounds with multi-trypanosomatid activity have drug like properties.

## Graphical abstract

**Figure:**
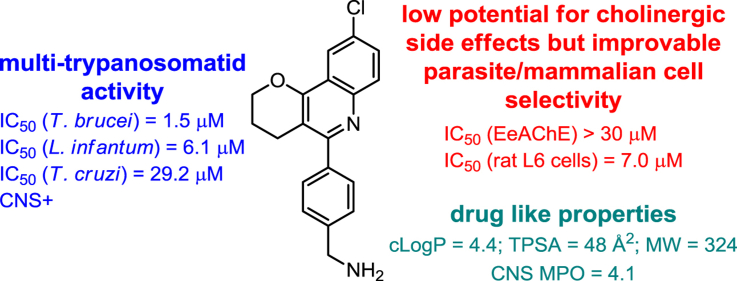

## 1 Introduction

Neglected tropical diseases (NTDs) are a group of 17 infectious diseases that globally affect more than 1 billion people from 149 countries [1]. Not only do NTDs cause a huge health impact, both in terms of disability-adjusted life years (DALY, 26 million DALYs) and mortality (534,000 deaths annually), but they also have harmful effects on the overall economic productivity of developing countries where these diseases are endemic, which become inexorably trapped in an unbreakable cycle of poverty [2], [3], [4].

Among NTDs, vector-borne kinetoplastid diseases are particularly deadly, with leishmaniasis, Chagas disease (or American trypanosomiasis) and human African trypanosomiasis (HAT or sleeping sickness) ranking first, fifth and sixth, respectively, in number of associated deaths [2]. Their causative agents are trypanosomatid parasites that are transmitted to humans through the intervention of an infected insect vector: *Trypanosoma brucei gambiense* and *T. brucei rhodesiense* (accounting for 98% and 2% of cases of HAT, respectively) spread through the bite of blood-feeding tsetse flies; *Trypanosoma cruzi* (for Chagas disease) transmitted most commonly through contact with the faeces of a blood-sucking triatomine bug (the so-called kissing bug); and different *Leishmania* species, prominently *Leishmania donovani* and *Leishmania infantum* (for visceral leishmaniasis), transmitted by the bite of female phlebotomine sand flies. In the absence of treatment, these diseases are frequently fatal, with their mortality being associated to particular stages or forms of the disease. In the case of HAT, after an initial hemo-lymphatic stage characterized by nonspecific clinical symptoms, parasites can cross the blood–brain barrier (BBB) and invade the central nervous system (CNS), giving rise to an array of severe neurological manifestations that include profound sleep disruptions and eventually coma and death. The initial phases of Chagas disease are usually asymptomatic or associated with non-specific symptoms of fever, malaise, or lymph node enlargement, but in about 30% of patients it evolves into a chronic phase, usually characterized by cardiomyopathy, and is a major cause of premature heart failure in Latin America. The most severe manifestation of leishmaniasis is visceral form, which leads to hepatosplenomegaly, progressive anaemia, and ultimately death in most cases.

These diseases are usually confined to rural areas of endemic countries (sub-Saharan Africa for HAT, mainly Central and South America for Chagas disease, and Middle East and Asia, East Africa, Central and South America and Southern Europe for leishmaniasis). However, climate changes due to global warming, which may result in an extension of the insect vector habitats, as well as international travel and immigration patterns may expand the geographical impact of these infectious diseases, thereby increasing the population at risk [5]. Travel to and immigration from endemic countries have made Chagas disease and several forms of leishmaniasis emerging infections in the United States (both infections), and Spain and Japan (Chagas disease) [6], [7].

The current therapies against HAT, Chagas disease and leishmaniasis suffer from important shortcomings. HAT first-stage treatments rely on pentamidine and suramin, which require parenteral administration and are ineffective against the second stage. Stage 2 HAT can be treated by painful intravenous administration of the arsenical drug melarsoprol, which may lead to fatal reactive encephalopathy in 5–10% of patients, or with eflornithine, which is much safer but requires intravenous administration and hospitalization [8]. Toxicity is also a major issue with the approved drugs against Chagas disease, the nitroderivatives benznidazole and nifurtimox, and with some of the drugs used for the treatment of visceral leishmaniasis (pentavalent antimonials, amphotericin B, paromomycin and miltefosine). Apart from complicated long courses of treatment, in most cases parenteral administration is required. In addition, the emergence of resistance to these drugs in areas of high transmission further challenges their clinical application [6], [8], [9], [10], [11], [12], [13].

In the absence of preventive or therapeutic vaccines and rigorous control of insect vectors [5], [14], the development of novel chemotherapies against these infectious diseases, with appropriate efficacy and safety profiles, is desperately needed [15], [16]. Besides combinations of approved antiprotozoan drugs or repurposing of known drugs with other indications [8], [14], [15], increasing research efforts are being made to design novel chemical entities that hit one or several biological targets which play a key role in the biology of the parasite and are sufficiently different from those in the mammalian host cells as to enable selective toxicity [5], [9], [17], [18], [19], [20], [21], [22], [23]. However, while we are gaining a better understanding of the relevant parasite targets, phenotypic whole cell screening of novel compounds or chemical libraries remains a very successful approach for anti-protozoan drug discovery [8], [24], [25]. Thus, anti-protozoan drug pipelines are being enriched through drug discovery campaigns involving the synthesis of novel chemical entities and their biological evaluation against the selected parasites [26], [27], [28]. Of particular interest are those compounds that can be active against several protozoan parasites [25], [29], [30], [31], as several NTDs usually coexist in endemic countries [1]. The trypanosomatid parasites that cause HAT, Chagas disease and leishmaniasis are taxonomically related, have similar structural and biochemical features, and seem to share many of their metabolic pathways [14], thereby rendering them especially amenable to modulation by common drugs. Indeed, several structural families featuring a 4-aminoquinoline moiety have been recently reported to display a multi-protozoan profile, namely trypanocidal and antiplasmodial activity [32], [33], [34], [35].

**Fig. 1:**
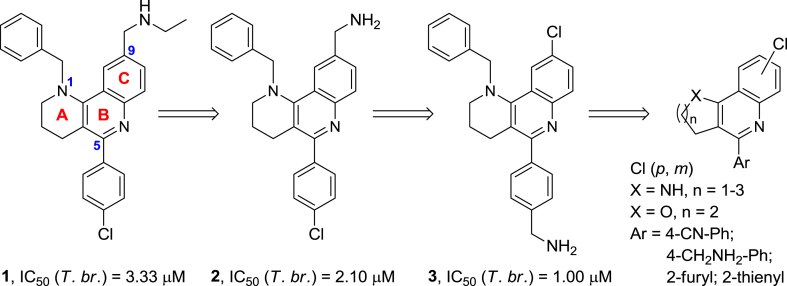
Design of the novel benzonaphthyridines **2** and **3** and other heterofused quinoline analogues from compound **1**.

The use of multicomponent reactions [36] appears to be a very useful strategy to rapidly build new hits in a modular manner. This approach is having a tremendous impact in modern medicinal chemistry. Apart from considerably speeding the process and manufacture of some drugs [37], it is especially relevant in drug discovery. It allows the preparation of new scaffolds and their straightforward decoration, and also facilitates the hit to lead transition and pharmacological issues [38], [39]. We have recently reported the synthesis and acetylcholinesterase (AChE) inhibitory activity of a series of 1,2,3,4-tetrahydrobenzo[*h*][1,6]naphthyridines such as **1** (Fig. 1), which are prepared using a multicomponent Povarov reaction as the key step [40]. This transformation requires a cyclic enamide as an activated olefin, which affords the ring A of **1** (Fig. 1), an aromatic aldehyde, which affords the substituent at position 5, and an aniline, which affords the ring C with the substituent at position 9. Because the tricyclic scaffold of the benzo[*h*][1,6]naphthyridine system of **1** contains a 4-aminoquinoline motif and a side chain with a second protonatable nitrogen atom, we inferred that this compound might display some anti-protozoan activity. Indeed, the outstanding IC_50_ value of 3.33 μM against *T. brucei* that we have found for **1** has confirmed our initial assumption. In light of this, and relying on the synthetic versatility of the multicomponent Povarov reaction [41], [42], which might enable the modification of ring A and the substituents at positions 1, 5 and 9 of benzonaphthyridine **1** by simply changing the starting materials, we planned the synthesis of a series of analogues of **1** and their evaluation against the trypanosomatids that cause HAT, Chagas disease and leishmaniasis.

Here, we report i) the synthesis of novel benzo[*h*][1,6]naphthyridine-, pyrrolo[3,2-*c*]quinoline-, azepino[3,2-*c*]quinoline-, and pyrano[3,2-*c*]quinoline-based analogues of **1** with different substituents at rings A, B, and C, as well as some quinoline derivatives resulting from opening of ring A of some of these tricyclic scaffolds; ii) their evaluation against *T. brucei*, *T. cruzi* and *L. infantum*; iii) and the assessment of their cytotoxic activity against rat myoblast L6 cells and their AChE inhibitory activity. Also, to determine their potential usefulness for late-stage HAT, the ability of the novel compounds to cross the BBB has been evaluated *in vitro* using a parallel artificial membrane permeability assay (PAMPA-BBB).

## 2 Results and discussion

### 2.1 Design and synthesis of the target compounds

**Scheme 1:**
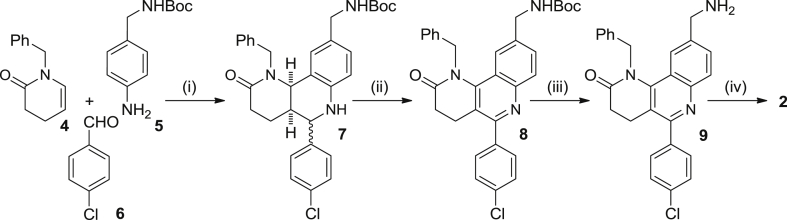
Reagents and conditions: (i) **5**, **6**, Sc(OTf)_3_, CH_3_CN, rt, 5 min; then, **4**, CH_3_CN, rt, 3 days; (ii) DDQ, CHCl_3_, rt, overnight, 47% overall; (iii) 4M HCl/dioxane, rt, overnight, 44%; (iv) (EtO)_3_SiH, Zn(OAc)_2_, THF, rt, 30 min; then, **9**, THF, 65 °C, 48 h, 66%.

**Scheme 2:**
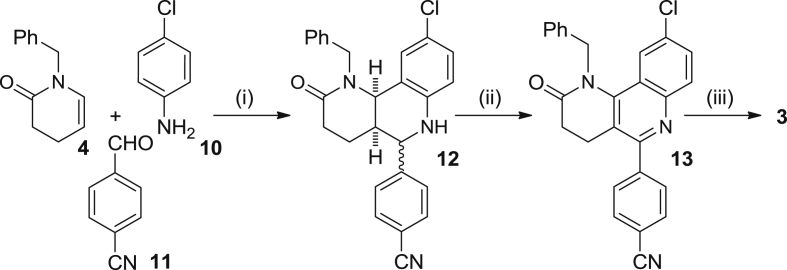
Reagents and conditions: (i) **10**, **11**, Sc(OTf)_3_, CH_3_CN, rt, 5 min; then, **4**, CH_3_CN, rt, 3 days; (ii) DDQ, CHCl_3_, rt, overnight, 35% overall; (iii) LiAlH_4_, THF, reflux, overnight, 22%.

Compound **1** is a rather quite lipophilic molecule, with a calculated log P value of 6.64 [43], hence clearly above the commonly accepted threshold for good oral bioavailability [44]. In order to decrease lipophilicity, we first envisaged the synthesis of compound **2**, i.e. the *N*-deethylated derivative of **1**, and its isomer **3** (Fig. 1 and Scheme 1, Scheme 2), in which the chloro and aminomethyl substituents at the *para* position of the phenyl substituent and at position 9, respectively, were interchanged. Interestingly, both analogues turned out to be more potent against *T. brucei* than hit **1** (Fig. 1, see Section 2.2), especially compound **3**, which was 3-fold more potent, albeit still too lipophilic (log P = 5.89).

**Scheme 3:**
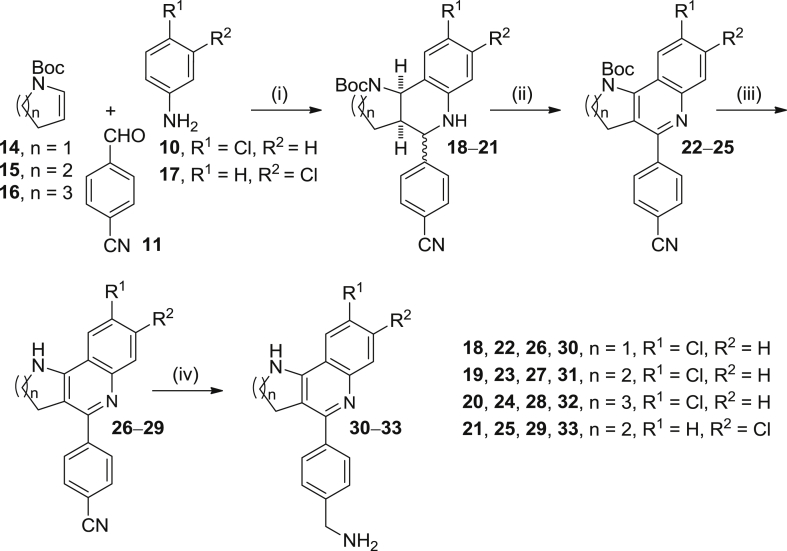
Reagents and conditions: (i) **10** or **17**, **11**, Sc(OTf)_3_, CH_3_CN, rt, 5 min; then, **14**, **15** or **16**, CH_3_CN, rt, 3 days; (ii) DDQ, CHCl_3_, rt, overnight, 59% (**22**), 69% (**23**), 79% (**24**), and 62% (**25**) overall; (iii) 4M HCl/dioxane, rt, overnight, 46% (**26**), 68% (**27**), 51% (**28**), and 94% (**29**); (iv) LiAlH_4_, THF, reflux, overnight, 74% (**30**), 87% (**31**), 55% (**32**), and 67% (**33**).

**Scheme 4:**
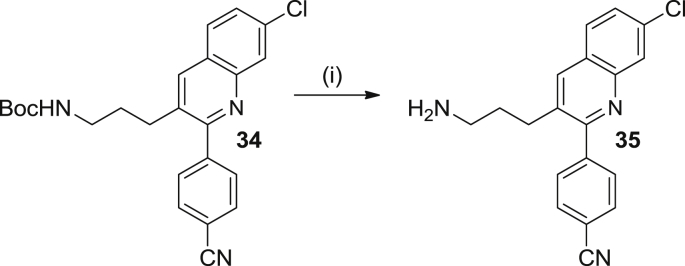
Reagents and conditions: (i) 4M HCl/dioxane, rt, overnight, 90%.

**Scheme 5:**
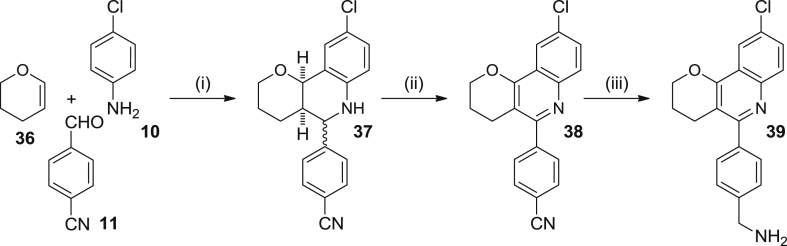
Reagents and conditions: (i) **10**, **11**, Sc(OTf)_3_, CH_3_CN, rt, 5 min; then, **36**, CH_3_CN, rt, 3 days, 95% (1:1 diastereomeric mixture); (ii) DDQ, CHCl_3_, rt, overnight, 41%; (iii) LiAlH_4_, THF, reflux, overnight, 59%.

**Scheme 6:**
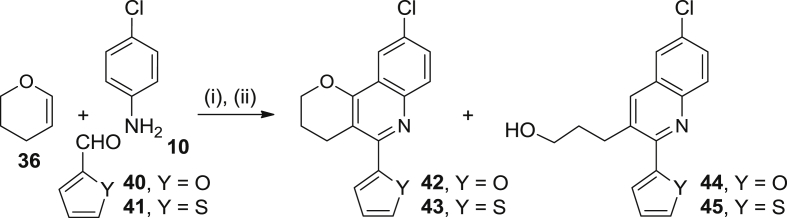
Reagents and conditions: (i) **10**, **40** or **41**, Sc(OTf)_3_, CH_3_CN, rt, 5 min; then, **36**, CH_3_CN, 50 °C (for **42**) or 80 °C (for **43**), 3 days; (ii) DDQ, CHCl_3_, rt, overnight, 27% (**42**) and 30% (**44**), 40% (**43**) and 24% (**45**) overall.

**Scheme 7:**
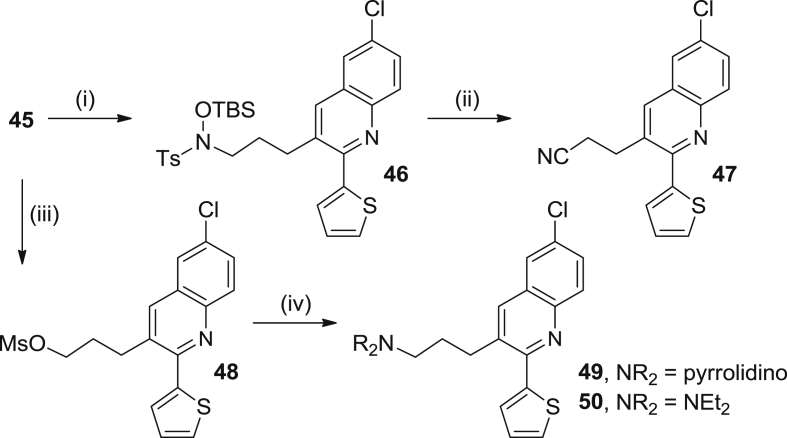
Reagents and conditions: (i) *N*-(*tert*-butyldimethylsilyloxy)-4-methylbenzenesulfonamide, PPh_3_, DEAD, toluene, THF, rt, 5 min; (ii) CsF, CH_3_CN, 60 °C, 2.5 h, 84% overall; (iii) MsCl, Et_3_N, 0 °C, 30 min; (iv) pyrrolidine or Et_2_NH, K_2_CO_3_, DMF, 85 °C, overnight, 31% (**49**), 16% (**50**) overall.

In this regard, we next envisioned a series of modifications around the structure of compound **3**, aimed at improving drug likeness and deriving structure–activity relationships first against *T. brucei*, and then against *T. cruzi* and *L. infantum*. Because removal of the benzyl group at position 1 of compound **3** should lead to a significant decrease of both lipophilicity and molecular weight and, hence, to improved drug likeness, we planned the synthesis of the *N*_1_-unsubstituted derivative **31** and its analogues resulting from ring contraction and ring expansion (**30** and **32**, respectively, Scheme 3), isomerization of the chlorine substituent from position 9 to position 8 (**33**, Scheme 3), and NH → O bioisosteric replacement (**39**, Scheme 5), using the corresponding nitriles as the immediate precursors. Moreover, to assess the relevance of the 4-aminomethylphenyl substituent at the B-ring, we decided to study the biological activity of the nitrile precursors (i.e. **26**–**29** and **38**, Scheme 3, Scheme 5), as well as that of compounds **42** and **43**, in which the 4-aminomethylphenyl group at the B-ring of compound **39** was replaced by a 2-furyl- or 2-thienyl- substituent (Scheme 6). During the synthesis of the target tricyclic heterofused quinoline compounds some byproducts arising from opening of the A-ring were obtained (see below) and, eventually, converted into additional target compounds featuring cyano, hydroxy or amino groups at the side chain (i.e. **35**, **44**, **45**, **47**, **49** and **50**, Scheme 4, Scheme 6, Scheme 7).

The synthesis of compound **2** was carried out by the four-step sequence depicted in Scheme 1. The multicomponent Povarov reaction between the known unsaturated lactam **4** as the activated olefin [45], [46], and commercially available 4-chlorobenzaldehyde, **6**, and aniline **5**, bearing an *N*-Boc-protected aminomethyl side chain, under Sc(OTf)_3_ catalysis in acetonitrile, followed by DDQ oxidation [47] of the resulting diastereomeric mixture of octahydrobenzonaphthyridines **7** afforded compound **8** in 47% overall yield, after silica gel column chromatography purification. *N*-Boc deprotection of **8**, followed by reduction of the resulting lactam **9** with (EtO)_3_SiH under Zn(OAc)_2_ catalysis [48], and silica gel column chromatography purification afforded the target benzonaphthyridine **2**, in 29% overall yield for the last two steps (Scheme 1).

The synthesis of compound **3** required only 3 steps, starting with a multicomponent Povarov reaction between the enamide **4**, 4-chloroaniline, **10**, and the aromatic aldehyde **11**, bearing a 4-cyano group as the precursor of the aminomethyl side chain. DDQ oxidation of the resulting diastereomeric mixture of octahydrobenzonaphthyridines **12**, followed by simultaneous LiAlH_4_ reduction of the lactam and nitrile functionalities of compound **13** afforded the target benzonaphthyridine **3** in low overall yield, after silica gel column chromatography purification (Scheme 2).

For the synthesis of the *N*_1_-debenzylated analogues **30**–**33** we used a 4-step protocol that involved an initial multicomponent Povarov reaction between chloroanilines **10** or **17**, cyano aldehyde **11** and commercially available *N*-Boc-protected cyclic enamines **14**–**16**, followed by DDQ oxidation, *N*-Boc acidic deprotection, and final LiAlH_4_ reduction of nitriles **26**–**29** (Scheme 3). After silica gel column chromatography purification, the target compounds **30**, **31**, **32**, and **33** were obtained in 20%, 41%, 22%, and 39% overall yield, respectively.

Of note, during the synthesis of compound **25**, a significant amount (17% yield) of ring-open by-product **34** (Scheme 4) was also formed. This compound was subjected to the standard acidic conditions for *N*-Boc deprotection, affording amine **35** in 90% yield (Scheme 4).

The synthesis of pyranoquinoline **39** was envisaged through a three-step sequence, analogous to that used for compound **3**, but starting from 3,4-dihydro-2*H*-pyran, **36**, as the activated olefin for the Povarov reaction instead of the enamide **4** (Scheme 5).

Because we wanted to assess the influence on anti-protozoan activity of the degree of oxidation of the B-ring of the heterofused quinoline compounds, at this point we decided to isolate the tetrahydroquinoline compound resulting from the Povarov reaction, before performing the oxidation to the final quinoline derivative. Thus, after the reaction between the cyclic enol ether **36**, the aniline **10** and the aldehyde **11**, the crude product was purified by silica gel column chromatography to obtain a 1:1 diastereomeric mixture **37** in 95% yield. After a second column chromatography from this material, a sample of the all-*cis*-diastereoisomer, all-*cis*-**37**, was isolated to be subjected to biological evaluation (see below). Eventually, the pyranoquinoline **39** was obtained in 23% overall yield by DDQ oxidation of the diastereomeric mixture **37** to the quinoline derivative **38**, followed by LiAlH_4_ reduction of the nitrile to an aminomethyl group (Scheme 5).

Analogously to the synthesis of **39**, starting from the cyclic enol ether **36** and aniline **10** but changing now the aromatic aldehyde to furan-2-carboxaldehyde and thiophene-2-carboxaldehyde, **40**, and **41**, respectively, the target pyranoquinoline derivatives **42** and **43**, substituted at position 5 with a 2-furyl or 2-thienyl group instead of an aminomethylphenyl substituent, were obtained in 27% and 40% yield, respectively, together with significant amounts of the ring-open byproducts **44** (30% yield) and **45** (24% yield), respectively (Scheme 6).

Finally, the Mitsunobu reaction of compound **45** with *N*-(*tert*-butyldimethylsilyloxy)-4-methylbenzenesulfonamide followed by treatment with CsF in acetonitrile afforded nitrile **47** in 84% overall yield, after silica gel column chromatography purification. Moreover, alcohol **45** was converted *via* mesylate into the corresponding amines **49** and **50** in 31% and 16% overall yield, respectively (Scheme 7).

All the compounds to be subjected to biological evaluation, except **37**, were transformed into the corresponding hydrochloride or dihydrochloride salts, and were chemically characterized through IR, ^1^H and ^13^C NMR spectra, HRMS and HPLC purity analysis and elemental analysis.

### 2.2 Biological profiling of the novel heterofused quinoline compounds and ring-open analogues

#### 2.2.1 In vitro activity against T. brucei

As previously mentioned, we first evaluated the activity of **1**, a compound that we had developed as an inhibitor of AChE [40], and the novel heterofused quinoline and ring-open analogues *in vitro* against cultured bloodstream forms of *T. brucei*, using nifurtimox as a reference compound. We also investigated their brain permeability to assess their potential usefulness in late-stage HAT. To determine potential toxic effects, their cytotoxicity against rat skeletal myoblast L6 cells and AChE inhibitory activity, which might result in cholinergic side-effects, were also evaluated.

All the novel heterofused quinoline derivatives featuring a protonatable aminomethylphenyl group at the B-ring, including the benzo[*h*][1,6]naphthyridines **2**, **3**, **31** and **33**, the pyrrolo[3,2-*c*]quinoline **30**, the azepino[3,2-*c*]quinoline **32**, and the pyrano[3,2-*c*]quinoline **39**, were found to be 1.5–4-fold more potent than hit **1** and 2–5-fold more potent than the reference compound nifurtimox, with IC_50_ and IC_90_ values in the range 0.92–2.44 μM and 1.19–3.48 μM, respectively (Table 1). In contrast, their nitrile precursors **26**–**29**, **37**, and **38**, as well as the 5-(2-furyl)- and 5-(2-thienyl)-substituted pyrano[3,2-*c*]quinolines **42** and **43**, all of them bearing a neutral group at the B-ring, were clearly less potent. This highlights the relevance for trypanosomal activity of the presence of a protonatable group at the side chain of the substituent at the B-ring of the tricyclic heterofused quinoline derivatives.

**Table 1:** Trypanocidal (*T. brucei*), cytotoxic and anticholinesterase activity and BBB permeability of the novel heterofused quinolines and related compounds.^a^

Compd	*T. brucei* IC_50_ (μM)	*T. brucei* IC_90_ (μM)	L6 cells IC_50_ (μM)	SI_*Tb*_^b^	*P*_e_ (10^−6^ cm s^−1^)^c^ (prediction)	*Ee*AChE IC_50_ (μM)
**1**	3.33 ± 0.18	4.39 ± 0.04	7.08 ± 0.11	2.1	d	0.15 ± 0.01^e^
**2**	2.10 ± 0.07	2.90 ± 0.02	7.99 ± 0.25	3.8	d	1.05 ± 0.18
**3**	1.00 ± 0.03	1.19 ± 0.02	6.78 ± 1.26	6.8	7.4 ± 0.2 (CNS+)	3.67 ± 0.55
**26**	>30	>30	d	d	6.8 ± 0.3 (CNS+)	1.19 ± 0.15
**27**	6.07 ± 0.54	10.5 ± 0.2	38.8 ± 1.7	6.4	14.3 ± 0.1 (CNS+)	1.13 ± 0.17
**28**	17.3 ± 2.1	28.6 ± 6.7	d	d	20.0 ± 1.1 (CNS+)	2.20 ± 0.30
**29**	>30	>30	d	d	16.2 ± 0.1 (CNS+)	3.27 ± 0.43
**30**	0.92 ± 0.05	1.57 ± 0.03	2.54 ± 0.92	2.8	7.4 ± 2.6 (CNS+)	0.87 ± 0.08
**31**	2.44 ± 0.13	3.48 ± 0.05	8.92 ± 0.25	3.7	4.6 ± 0.2 (CNS±)	0.97 ± 0.02
**32**	2.33 ± 0.12	3.35 ± 0.05	7.02 ± 0.27	3.0	11.6 ± 0.2 (CNS+)	1.20 ± 0.13
**33**	1.25 ± 0.05	2.12 ± 0.02	3.07 ± 0.66	2.5	4.0 ± 1.0 (CNS±)	0.44 ± 0.04
**35**	4.16 ± 0.07	5.61 ± 0.05	20.4 ± 0.4	4.9	18.3 ± 0.6 (CNS+)	d
**37**	>30	>30	d	d	7.0 ± 0.7 (CNS+)	d
**38**	9.61 ± 0.27	13.7 ± 1.0	36.3 ± 8.1	3.8	29.7 ± 3.8 (CNS+)	2.70 ± 0.45
**39**	1.46 ± 0.02	1.77 ± 0.02	7.00 ± 0.19	4.8	15.5 ± 1.8 (CNS+)	>30^f^
**42**	21.7 ± 1.2	29.3 ± 0.5	d	d	3.5 ± 0.4 (CNS±)	16.3 ± 1.5
**43**	18.0 ± 0.4	27.6 ± 1.1	d	d	14.4 ± 1.6 (CNS+)	9.39 ± 0.82
**44**	35.7 ± 1.8	70.6 ± 1.5	d	d	10.3 ± 1.5 (CNS+)	d
**45**	28.0 ± 0.7	42.9 ± 1.5	d	d	28.4 ± 1.2 (CNS+)	d
**47**	>30	>30	d	d	17.9 ± 1.1 (CNS+)	d
**49**	3.56 ± 0.17	4.93 ± 0.17	9.44 ± 0.14	2.7	12.9 ± 0.1 (CNS+)	∼30^g^
**50**	6.33 ± 0.94	12.6 ± 0.9	14.7 ± 2.3	2.3	20.9 ± 0.5 (CNS+)	d
nifurtimox	4.4 ± 0.7^h^		32.0 ± 1.1	7.3	d	d

a *In vitro* activity against bloodstream form of *T. brucei* (pH 7.4), rat myoblast L6 cells, and *Electrophorus electricus* AChE, expressed at the concentration that inhibited growth or enzyme activity by 50% (IC_50_) and 90% (IC_90_, for *T. brucei*). Data are the mean of triplicate experiments ± SEM.

b SI_*Tb*_: selectivity index as the ratio of cytotoxic to anti-*T. brucei* IC_50_ values.

c Permeability values from the PAMPA-BBB assay. Values are expressed as the mean ± SD of three independent experiments.

d Not determined.

e Taken from ref. [40], involving the same experimental conditions.

f 45% Inhibition at 30 μM (the IC_50_ could not be determined).

g 54% Inhibition at 30 μM (the IC_50_ could not be determined).

h Taken from Ref. [49].

Within the most potent tricyclic heterofused quinoline derivatives, the best substitution pattern for activity against *T. brucei* seems to involve i) Bn-N > O > NH at position 1 of the A-ring, with compound **3** being 1.5- and 2.4-fold more potent than **39** and **31**, respectively, ii) a five-membered A-ring, with compound **30** being 2.5-fold more potent than **31** and **32**, bearing a six- and seven-membered A ring, respectively, and iii) the presence of the chlorine atom at position *meta* relative to the quinoline nitrogen atom, with compound **33** being 2-fold more potent than **31**.

As in the tricyclic heterofused quinoline derivatives, the presence of a protonatable nitrogen atom at the side chain of ring-open quinoline analogues seems to be play a role in the activity of these compounds against *T. brucei*, with amines **35**, **49**, and **50** exhibiting IC_50_ values around 5 μM (Table 1), whereas compounds **44**, **45**, and **47**, with neutral hydroxy or cyano groups at the side chain were clearly less potent (IC_50_ > 25 μM).

#### 2.2.2 Brain permeation

Brain penetration is a desirable property for novel drugs against HAT, as it will make them effective against the late-stage disease, when parasites have invaded the CNS. Thus, the brain passive permeability of the novel compounds was evaluated *in vitro* through the widely used PAMPA-BBB method [50], which is based on the use of a porcine brain lipid extract as an artificial BBB model. The novel compounds had *in vitro* permeabilities (*P*_e_) clearly above the threshold established for a high BBB permeation, i.e. CNS+ with *Pe* (10^−6^ cm s^−1^) > 5.25, with the exceptions of compounds **31**, **33**, and **42**, for which an uncertain BBB permeation was predicted (Table 1).

Apart from a high brain passive permeability, a low P-glycoprotein (P-gp) efflux is a highly desirable property to ensure target engagement in the CNS. A number of knowledge-based approaches have emerged to assist the early phases of the drug discovery process, by addressing different aspects related with pharmacokinetic and pharmacodynamic properties to enhance the likelihood of deriving candidates with appropriate drug-like properties [51]. For compounds intended to act within the CNS, some molecular descriptor guidelines recommend to maintain topological polar surface area (TPSA) < 90 Å^2^ (preferably < 70 Å^2^) and the number of hydrogen bond donors (HBD) < 2 to maximize the probability of evading P-gp efflux [52], [53]. All of the novel compounds have TPSA values well below 70 Å^2^ and most of them also have a HBD number ≤ 2 (Table S1, Supplementary Material), so that they should be expected to show a low P-gp efflux liability.

To further support the lack of P-gp efflux liability in the novel compounds and gain additional insight into their CNS drug-likeness, we applied the Pfizer CNS multiparameter optimization (CNS MPO) algorithm [54]. This algorithm is based on a set of six physicochemical parameters, namely lipophilicity (cLogP), calculated distribution coefficient at pH 7.4 (cLogD), molecular weight, TPSA, number of HBD, and the p*K*_a_ value of the most basic centre, with all these parameters being weighted equally using a desirability score from 0 to 1. Increasing total CNS MPO scores for drugs has been correlated with increasing probabilities of appropriate pharmacokinetic and safety attributes, including high passive permeability and low P-gp efflux, amongst others. Particularly, drugs with CNS MPO desirability scores ≥4 (in a scale from 0 to 6) are expected to show a full alignment of the desired pharmacokinetic properties [54]. Interestingly, CNS MPO desirability scores ≥ 4 have been calculated for 12 out of the 21 novel compounds (Table S2, Supplementary Material), which supports their potential usefulness for treating late-stage HAT.

#### 2.2.3 Cytotoxicity and acetylcholinesterase inhibitory activity

To assess potential toxic effects of the novel derivatives, all the compounds with IC_50_ values against *T. brucei* below 10 μM were subjected to cytotoxicity studies using rat myoblast L6 cells. All the tested compounds were selective for *T. brucei* vs mammalian cells (selectivity indices (SI_*Tb*_) in the range 2.1–6.8 (Table 1), being more selective than the hit **1** but less selective than nifurtimox (SI_*Tb*_ = 7.3).

Because hit **1** was developed as an AChE inhibitor with potential application against Alzheimer's disease, this kind of activity might result in unwanted cholinergic side effects if the novel compounds were used as anti-protozoan agents. We therefore evaluated their AChE inhibitory activity using *Electrophorus electricus* AChE (*Ee*AChE), a widely used and affordable enzyme source for screening this activity. All the structural modifications carried out around the structure of hit **1** led to a decrease in *Ee*AChE inhibitory activity (3–110-fold). However, despite their decreased AChE inhibitory activity relative to hit **1**, most tested compounds were more potent against *Ee*AChE than against *T. brucei*. Only the benzonaphthyridine **3**, the pyranoquinoline **39** and the ring-open quinoline derivative **49** turned out to be more potent against *T. brucei* than against *Ee*AChE (4-, >20- and ∼8-fold, respectively).

Of note, hit **1** is 6-fold less potent against human recombinant AChE (hAChE) than against *Ee*AChE (IC_50_ = 0.15 μM compared with IC_50_ = 0.94 μM) [40]. Even though this might also be the case for the novel compounds reported here, future lead optimization should focus on decreasing AChE inhibitory activity and increasing selectivity indices.

#### 2.2.4 In vitro activity against T. cruzi and L. infantum

The occurrence of common metabolic pathways in trypanosomatid parasites makes it potentially feasible to develop anti-protozoan agents endowed with a multi-trypanosomatid profile [14], [55]. Thus, after having confirmed the significant activity against *T. brucei* of all the novel target heterofused quinoline derivatives and some of the ring-open analogues and assessed their brain permeation, cytotoxicity and AChE inhibitory activity, we undertook the evaluation of all the novel compounds against epimastigote forms of *T. cruzi* (strain MHOM/ES/2203/BCN590 (Tcl)) and promastigote forms of *L. infantum* (strain MCAN/ES/92/BCN722), using benznidazole and potassium antimony (III) tartrate hydrate as reference compounds.

Most of the tested compounds exhibited activity against *T. cruzi*, albeit with two-digit micromolar IC_50_ values (Table 2). Interestingly, there was a similar SAR profile as had been found with *T. brucei*. First, isomerization of the chlorine and aminomethyl substituents from compound **2** to **3** results in increased potency against *T. cruzi* (4-fold). Also, higher potencies were observed for those tricyclic heterofused quinoline analogues bearing a protonatable 4-(aminomethyl)phenyl group at the B-ring, relative to those bearing a 4-cyanophenyl, 2-furyl, or 2-thienyl groups. Thus, amines **30**, **31**, **32**, and **39** were 2–6-fold more potent than their nitrile precursors **26**, **27**, **28**, and **38**, respectively, and amine **39** was also 3- and 10-fold more potent than the 5-(2-furyl)- and 5-(2-thienyl)-substituted derivatives **42** and **43**, respectively. The sole exception was amine **33**, which turned out to be 2-fold less potent than its nitrile precursor **29**. Also, within the aminomethylphenyl-substituted analogues, the order of potencies related to the substitution at position 1 of the A-ring was: Bn-N > O > NH, with the *N*_1_-benzylated benzonapththyridine **3** being 3- and 5-fold more potent than pyranoquinoline **39** and *N*_1_-unsubstituted benzonapththyridine **31**. Regarding the size of the A-ring, again the presence of a five- or a seven-membered ring A led to increased potency relative to the derivatives with a six-membered A-ring, with the pyrroloquinoline **30** and the azepinoquinoline **32** being 1.5-fold more potent than the benzonaphthyridine **31**. For this activity, unlike against *T. brucei*, a higher potency seems to arise from the presence of a chlorine atom at position *para* relative to the quinoline nitrogen atom, with compound **31** being 1.5-fold more potent than **33**. Overall, the most potent analogue of the series against *T. cruzi* was compound **3**, which exhibited an IC_50_ value of 9.47 μM, 4-fold more potent than the reference compound benznidazole. In addition, six other derivatives turned out to be slightly more potent or equipotent to benznidazole, with IC_50_ values around 30 μM. However, all the novel compounds were clearly less selective than benznidazole for *T. cruzi* vs mammalian cells, with SI_*Tc*_ < 1 for the novel compounds and 14.1 for benznidazole (Table 2).

**Table 2:** *In vitro* activity of the novel heterofused quinolines and related compounds against epimastigotes of *Trypanosoma cruzi* and promastigotes of *Leishmania infantum*.^a^

Compd	*T. cruzi* IC_50_ (μM)	*L. infantum* IC_50_ (μM)	SI_*Tc*_^b^	SI_*Li*_^c^
**2**	38.1 ± 9.5	8.1 ± 1.9	0.2	1.0
**3**	9.5 ± 2.5	31.1 ± 2.9	0.7	0.2
**26**	>150	>200	d	d
**27**	147 ± 19	15.3 ± 3.6	0.3	2.5
**28**	64.6 ± 12.5	13.7 ± 2.2	d	d
**29**	39.4 ± 0.2	19.0 ± 3.0	d	d
**30**	37.3 ± 11.0	6.5 ± 1.4	0.1	0.4
**31**	50.5 ± 14.3	11.6 ± 2.5	0.2	0.8
**32**	32.4 ± 0.6	4.8 ± 0.7	0.2	1.5
**33**	76.1 ± 3.2	13.0 ± 0.6	0.04	0.2
**35**	78.8 ± 1.7	16.2 ± 4.0	0.3	1.3
**37**	676 ± 37	>200	d	d
**38**	178 ± 92	23.5 ± 2.4	0.2	1.5
**39**	29.2 ± 3.7	6.1 ± 0.2	0.2	1.1
**42**	76.1 ± 7.8	38.4 ± 21.6	d	d
**43**	>250	51.4 ± 2.3	d	d
**44**	29.3 ± 12.0	19.3 ± 11.3	d	d
**45**	51.7 ± 19.4	37.3 ± 2.3	d	d
**47**	51.7 ± 5.1	23.3 ± 5.9	d	d
**49**	35.2 ± 12.6	13.1 ± 5.8	0.3	0.7
**50**	77.6 ± 11.2	13.6 ± 1.5	0.2	1.1
Benznidazole	36.2 ± 5.1	d	14.1^e^	d
Sb (III)^f^	d	24.3 ± 1.7	d	0.1^g^

a *In vitro* activity against epimastigote form of *T. cruzi* and promastigote form of *L. infantum*, expressed at the concentration that inhibited growth by 50% (IC_50_). Data are the mean ± SD of duplicate experiments performed at least twice.

b SI_*Tc*_: selectivity index as the ratio of cytotoxic to anti-*T. cruzi* IC_50_ values.

c SI_*Li*_: selectivity index as the ratio of cytotoxic to anti-*L. infantum* IC_50_ values.

d Not determined.

e IC_50_ value against rat myoblast L6 cells = 510 ± 22.

f Potassium antimony (III) tartrate hydrate.

g IC_50_ value against rat myoblast L6 cells = 3.27 ± 0.24.

All of the novel compounds turned out to be leishmanicidal agents, with IC_50_ values in the low micromolar range in most cases, being more potent (up to 5-fold) than the reference compound potassium antimony (III) tartrate hydrate (Table 2). Thus, these compounds were more active against *L. infantum* than against *T. cruzi*, with the sole exception of compound **3**, the most potent antichagasic derivative of the series. Even though the novel compounds were 2–25-fold more selective for *L. infantum* vs mammalian cells than potassium antimony (III) tartrate hydrate (SI_*Li*_ = 0.1), their selectivity indices were rather low (SI_*Li*_ in the range 0.2–2.5) (Table 2).

Some of the SARs of the leishmanicidal activity of the novel tricyclic heterofused quinoline analogues were very similar to those found for anti-trypanosome activities, with the best substitution pattern involving the presence of: i) a protonatable 4-(aminomethyl)phenyl group at the B-ring, with amines **30**, **31**, **32**, **33**, and **39** being 1.5 to >31-fold more potent than their nitrile precursors **26**, **27**, **28**, **29**, and **38**, respectively, and amine **39** being 6- and 8-fold more potent than the 5-(2-furyl)- and 5-(2-thienyl)-substituted derivatives **42** and **43**, respectively; ii) a five- or a seven-membered A-ring, with the pyrroloquinoline **30** and the azepinoquinoline **32** being approximately 2-fold more potent than the benzonaphthyridine **31**, bearing a six-membered ring A; and iii) an aromatic B-ring, with compound **38** being >9-fold more potent than the saturated analogue **37**. For this activity, the presence of an oxygen atom a position 1 of the A-ring led to increased potency relative to an NH group, with compound **39** being 2-fold more potent than **31**. However, a reverse SAR trend relative to those found for the anti-trypanosome activities was observed regarding the presence of a Bn-N group at position 1 of the A-ring and the isomerization of the chlorine and aminomethyl substituents. Thus, the presence of a Bn-N group at position 1 of the A-ring was detrimental for leishmanicidal activity, with compound **3** being 3- and 5-fold less potent than the NH- and O-substituted counterparts **31** and **39**, respectively, whereas the interchange of the chlorine and aminomethyl substituents from compound **2** to **3** resulted in decreased potency against *L. infantum* (4-fold). The position of the chlorine substituent at the C-ring had no influence on the leishmanicidal activity of the novel compounds. Of note, as found when measuring *T. brucei* activities, the three ring-open analogues featuring a protonatable amino group at the side chain, i.e. **35**, **49**, and **50**, exhibited significant leishmanicidal activity, with IC_50_ values around 15 μM (Table 2).

The most potent analogues of the series against *L. infantum* were benzonaphthyridines **2**, **30**, and **32**, and pyranoquinoline **39**, with IC_50_ values in the range 5–8 μM.

## 3 Conclusion

We have synthesized a series of tricyclic heterofused quinolines, namely benzo[*h*][1,6]naphthyridines, pyrrolo[3,2-*c*]quinolines, azepino[3,2-*c*]quinolines, and pyrano[3,2-*c*]quinolines, through 2–4-step synthetic sequences that involve as the key step an initial Povarov multicomponent reaction between a cyclic enamide or enol ether as an activated olefin and a properly substituted aniline and aromatic aldehyde. The novel compounds have been designed from benzonaphthyridine **1**, a submicromolar inhibitor of AChE previously developed in our group that has been found to display a significant *in vitro* activity against *T. brucei*. Initial structural modifications around hit **1**, including *N*-dealkylation of the side chain at position 9 and isomerization by interchange of the chlorine atom at the *para* position of the 5-phenyl group and the aminomethyl substituent at position 9, have led to benzonaphthyridine **3**, which has turned out to be 3-fold more potent than hit **1** against *T. brucei*. The structure of compound **3** has been further modified by *N*_1_-debenzylation, A-ring contraction and expansion, bioisosteric NH → O replacement at position 1, and substitution of the 5-(4-aminomethyl)phenyl group by 5-(2-furyl) and 5-(2-thienyl). During the synthesis of the target tricyclic compounds, some quinoline derivatives with a side chain at position 3, arising from opening of the A-ring, were obtained. To further expand the SAR studies, the structure of these ring-open derivatives was subsequently modified by introduction of a protonatable amino group or a neutral cyano group at the end of the side chain.

Trypanosomatid parasites responsible for HAT, Chagas disease and visceral leishmaniasis seem to share common metabolic pathways [14], [55], thereby being potentially amenable to treatment by common drugs. Consistent with this, we have found some common SAR trends related to the activities of the novel tricyclic heterofused quinoline analogues against *T. brucei*, *T. cruzi* and *L. infantum*, with several of these compounds being moderately potent against two or three of these parasites. Thus, the presence of an oxidized B-ring featuring a protonatable 4-(aminomethyl)phenyl group and the bioisosteric NH → O replacement at position 1 led to higher potencies against the three parasites. Benzonaphthyridines **2**, **30** and **32**, and pyranoquinoline **39** exhibit an interesting multi-trypanosomatid profile, with single digit micromolar IC_50_ values against *T. brucei* and *L. infantum* and IC_50_ around 30 μM against *T. cruzi*, whereas benzonaphthyridine **3** exhibits single digit micromolar IC_50_ values against *T. brucei* and *T. cruzi* and IC_50_ around 30 μM against *L. infantum*. Interestingly, all of these multi-trypanosomatid compounds have been predicted to be able to cross the BBB, which is of utmost importance for the treatment of late-stage HAT, and have better drug-like properties than hit **1**, both in terms of lower lipophilicity and molecular weight. They also display higher CNS MPO desirability scores, and hence, are expected to be endowed with more appropriate and aligned pharmacokinetic attributes, including low P-gp efflux. A significant AChE inhibitory activity in most of these compounds, albeit lower than that of hit **1**, and significant toxicity to rat L6 cells are their main drawbacks, which should be addressed in further lead optimization. To this end, the dual trypanocidal and leishmanicidal pyranoquinoline **39**, which displays the lowest AChE inhibitory activity, and hence, the lowest potential for cholinergic side effects, is likely to be the best starting point.

## 4 Experimental part

### 4.1 Chemistry. General methods

Melting points were determined in open capillary tubes with a MFB 595010M Gallenkamp melting point apparatus. 400 MHz ^1^H/100.6 MHz ^13^C NMR spectra were recorded on a Varian Mercury 400 spectrometer. The chemical shifts are reported in ppm (*δ* scale) relative to solvent signals (CD_3_OD at 3.31 and 49.0 ppm in the ^1^H and ^13^C NMR spectra, respectively; CDCl_3_ at 7.26 and 77.0 ppm in the ^1^H and ^13^ C NMR spectra, respectively), and coupling constants are reported in Hertz (Hz). Assignments given for the NMR spectra of the new compounds have been carried out by comparison with the NMR data of **3**, **9**, **28**, **37**, **42**, **43**, **44**, **45**, **47**, and **50**, which in turn, were assigned on the basis of DEPT, COSY ^1^H/^1^H (standard procedures), and COSY ^1^H/^13^C (gHSQC or gHMBC sequences) experiments. IR spectra were run on a Perkin–Elmer Spectrum RX I or on a Thermo Nicolet Nexus spectrophotometer. Absorption values are expressed as wavenumbers (cm^−1^); only significant absorption bands are given. Column chromatography was performed on silica gel 60 AC.C (35–70 mesh, SDS, ref 2000027). Thin-layer chromatography was performed with aluminium-backed sheets with silica gel 60 F_254_ (Merck, ref 1.05554), and spots were visualized with UV light and 1% aqueous solution of KMnO_4_. NMR spectra of all of the new compounds were performed at the Centres Científics i Tecnològics of the University of Barcelona (CCiTUB), while elemental analyses and high resolution mass spectra were carried out at the Mycroanalysis Service of the IIQAB (CSIC, Barcelona, Spain) with a Carlo Erba model 1106 analyser, and at the CCiTUB with a LC/MSD TOF Agilent Technologies spectrometer, respectively. The HPLC measurements were performed using a HPLC Waters Alliance HT apparatus comprising a pump (Edwards RV12) with degasser, an autosampler, a diode array detector and a column as specified below. The reverse phase HPLC determinations were carried out on a YMC-Pack ODS-AQ column (50 × 4.6 mm, D S. 3 μm, 12 nm). Solvent A: water with 0.1% formic acid; Solvent B: acetonitrile with 0.1% formic acid. Gradient: 5% of B to 100% of B within 3.5 min. Flux: 1.6 mL/min at 50 °C. The analytical samples of all of the compounds that were subjected to pharmacological evaluation were dried at 65 ºC/2 Torr for at least 2 days (standard conditions) and possess a purity ≥95% as indicated by their elemental analyses and/or HPLC measurements.

#### 4.1.1 1-Benzyl-9-(tert-butoxycarbonylaminomethyl)-5-(4-chlorophenyl)-1,2,3,4-tetrahydro-2-oxobenzo[h][1,6]naphthyridine **8**

To a stirred solution of *p*-chlorobenzaldehyde, **6** (1.16 g, 8.25 mmol) and aniline **5** (1.83 g, 8.23 mmol) in anhydrous CH_3_CN (30 mL), 4 Å molecular sieves and Sc(OTf)_3_ (0.81 g, 1.65 mmol) were added. The mixture was stirred at room temperature under argon atmosphere for 5 min and then treated with a solution of enamine **4** (1.50 g, 8.01 mmol) in anhydrous CH_3_CN (16 mL). The resulting suspension was stirred at room temperature under argon atmosphere for 3 days. Then, the resulting mixture was diluted with sat. aq. NaHCO_3_ (150 mL) and extracted with EtOAc (3 × 200 mL). The combined organic extracts were dried over anhydrous Na_2_SO_4_ and evaporated under reduced pressure to give a solid residue (4.71 g), mainly consisting of a diastereomeric mixture of octahydrobenzonaphthyridines **7**, which was used in the next step without further purification.

To a solution of crude diastereomeric mixture **7** (4.58 g of a crude of 4.71 g) in anhydrous CHCl_3_ (150 mL), DDQ (4.85 g, 21.4 mmol) was added. The reaction mixture was stirred at room temperature under argon atmosphere overnight, diluted with CH_2_Cl_2_ (150 mL) and washed with sat. aq. NaHCO_3_ (3 × 250 mL). The combined organic extracts were dried over anhydrous Na_2_SO_4_ and evaporated under reduced pressure to give a solid residue (5.33 g), which was purified through column chromatography (35–70 μm silica gel, hexane/EtOAc mixtures, gradient elution). On elution with hexane/EtOAc 80:20, compound **8** (1.94 g, 47% yield) was isolated as a white solid; *R*_*f*_ 0.83 (hexane/EtOAc 1:1).

A solution of **8** (30 mg, 0.06 mmol) in CH_2_Cl_2_ (5 mL) was filtered through a 0.2 μm PTFE filter and evaporated at reduced pressure. The solid was washed with pentane (3 × 5 mL) to give, after drying under standard conditions, the analytical sample of **8** (27 mg): mp 90–91 °C; IR (ATR) *ν* 3347 (NH st), 1691 (C=O, Ar–C–C and Ar–C–N st) cm^−1^; ^1^H NMR (400 MHz, CDCl_3_) *δ* 1.47 [s, 9H, C(CH_3_)_3_], 2.61 (t, *J* = 6.8 Hz, 2H, 4-H_2_), 2.90 (t, *J* = 6.8 Hz, 2H, 3-H_2_), 4.36 (d, *J* = 6.0 Hz, 2H, 9-C*H*_2_-NH), 4.96 (br s, 1H, 9-CH_2_-N*H*), 5.33 (s, 2H, 1-C*H*_2_-Ar), 7.12 [dd, *J* = 8.0 Hz, *J′* = 1.6 Hz, 2H, 1-CH_2_-Ar–C2(6)-*H*], 7.20–7.29 [complex signal, 3H, 1-CH_2_-Ar–C3(5)-*H*, 1-CH_2_-Ar–C4–*H*], 7.46 [dm, *J* = 8.8 Hz, 2H, 5-Ar–C3(5)-*H*], 7.53 [dm, *J* = 8.8 Hz, 2H, 5-Ar–C2(6)-*H*], 7.61 (dd, *J* = 8.8 Hz, *J′* = 1.6 Hz, 1H, 8-H), 7.77 (br s, 1H, 10-H), 8.11 (d, *J* = 8.8 Hz, 1H, 7-H); ^13^C NMR (100.6 MHz, CDCl_3_) *δ* 23.5 (CH_2_, C3), 28.4 [3CH_3_, C(*C*H_3_)_3_], 32.7 (CH_2_, C4), 44.5 (CH_2_, 9-CH_2_-NH), 52.2 (CH_2_, 1-*C*H_2_-Ar), 79.9 [C, *C*(CH_3_)_3_], 119.9 (C, C10a), 120.8 (CH), 127.3 (CH), 127.5 (CH), 128.5 (CH), 128.6 (CH), 130.5 (CH) (Ar–CH), 121.7 (C, C4a), 129.2 (C, C9), 135.1 (C, 1-CH_2_-Ar–*C*1), 137.4 (C), 137.5 (C) (5-Ar–*C*1, 5-Ar–*C*4), 146.8 (C, C6a), 147.7 (C, C5), 155.9 (C), 156.6 (C) (C10b, NCOO), 172.8 (C, C2); HRMS (ESI), calcd for [C_31_H_30_^35^ClN_3_O_3_ + H^+^] 528.2048, found 528.2045.

#### 4.1.2 N-{1-Benzyl-5-(4-chlorophenyl)-1,2,3,4-tetrahydro-2-oxobenzo[h][1,6]naphthyridin-9-yl}methanamine **9**

Compound **8** (1.94 g, 3.67 mmol) was dissolved in 4M HCl/dioxane solution (24 mL) at 0 °C. The mixture was stirred at room temperature overnight and concentrated *in vacuo*. The solid residue was diluted in water (20 mL), treated with 1N NaOH (20 mL), and the aqueous phase was extracted with a 10% MeOH/CHCl_3_ mixture (3 × 50 mL). The combined organic extracts were dried over anhydrous Na_2_SO_4_ and evaporated at reduced pressure to give a crude product (1.42 g), which was purified through column chromatography (35–70 μm silica gel, EtOAc/MeOH/50% aq. NH_4_OH, gradient elution). On elution with EtOAc/MeOH/50% aq. NH_4_OH 98.8:1:0.2 to 94.8:5:0.2, amine **9** (690 mg, 44% yield) was isolated as a yellow solid; *R*_*f*_ 0.78 (CH_2_Cl_2_/MeOH/50% aq. NH_4_OH 9:1:0.05).

A solution of **9** (130 mg, 0.30 mmol) in CH_2_Cl_2_ (5 mL) was filtered through a 0.2 μm PTFE filter and treated with a methanolic solution of HCl (0.53 N, 1.58 mL, 0.84 mmol). The resulting solution was evaporated at reduced pressure and the solid was washed with pentane (3 × 5 mL) to give, after drying under standard conditions, **9**·2HCl (119 mg) as a yellow solid: mp 218–220 °C; IR (ATR) *ν* 3500–2500 (max at 3386, 2919, 2850, 2610, ^+^NH and CH st), 1704 (C=O st), 1609, 1590, 1580, 1520, 1503 (C=O, Ar–C–C and Ar–C–N st) cm^−1^; ^1^H NMR (400 MHz, CD_3_OD) *δ* 2.83 (br t, *J* = 6.8 Hz, 2H, 3-H_2_), 3.09 (br t, *J* = 6.8 Hz, 2H, 4-H_2_), 4.28 (s, 2H, 9-C*H*_2_-NH_2_), 4.88 (s, ^+^NH, ^+^NH_3_), 5.57 (s, 2H, 1-CH_2_-Ar), 7.24–7.36 (complex signal, 5H, 1-CH_2_-Ar–*H*), 7.75 [br d, *J* = 8.0 Hz, 2H, 5-Ar–C3(5)-H], 7.82 [br d, *J* = 8.0 Hz, 2H, 5-Ar–C2(6)-H], 8.19 (br d, *J* = 8.8 Hz, 1H, 8-H), 8.29 (d, *J* = 8.8 Hz, 1H, 7-H), 8.43 (br s, 1H, 10-H); ^13^C NMR (100.6 MHz, CD_3_OD) *δ* 23.2 (CH_2_, C4), 31.8 (CH_2_, C3), 43.9 (CH_2_, 9-CH_2_-NH_2_), 54.7 (CH_2_, 1-*C*H_2_-Ar), 121.1 (C, C10a), 123.2 (CH, C7), 124.7 (C, C4a), 127.3 (CH, C10), 128.4 [2CH, 1-CH_2_-Ar–*C*2(6)], 128.8 (CH, 1-CH_2_-Ar–*C*4), 129.9 [2CH, 1-CH_2_-Ar–*C*3(5)], 130.7 [2CH, 5-Ar–*C*3(5)], 130.8 (C, 5-Ar–*C*1), 132.5 [2CH, 5-Ar–*C*2(6)], 134.9 (C, C9), 135.6 (CH, C8), 138.6 (C, 1-CH_2_-Ar–*C*1), 139.4 (C, 5-Ar–*C*4), 140.7 (C, C6a), 154.3 (C, C5), 156.9 (C, C10b), 173.3 (C, C2); HRMS (ESI), calcd for [C_26_H_22_^35^ClN_3_O + H^+^] 428.1524, found 428.1522.

#### 4.1.3 N-{1-Benzyl-5-(4-chlorophenyl)-1,2,3,4-tetrahydrobenzo[h][1,6]naphthyridin-9-yl}methanamine **2**

To a suspension of Zn(OAc)_2_ (49 mg, 0.27 mmol) in anhydrous THF (0.7 mL) (EtO)_3_SiH (0.10 mL, 89 mg, 0.54 mmol) was added. The mixture was stirred at room temperature for 30 min and then treated with a solution of amino lactam **9** (117 mg, 0.27 mmol) in anhydrous THF (2.1 mL). The reaction mixture was stirred at 65 °C for 48 h in a sealed vessel. The resulting mixture was cooled to room temperature, poured onto 1N NaOH (5 mL), stirred for 10 min, and then extracted with EtOAc (3 × 15 mL). The combined organic extracts were dried over anhydrous Na_2_SO_4_ and concentrated under reduced pressure to give a solid (313 mg), which was purified through column chromatography (35–70 μm silica gel, CH_2_Cl_2_/MeOH/50% aq. NH_4_OH mixtures, gradient elution). On elution with CH_2_Cl_2_/MeOH/50% aq. NH_4_OH 98.5:1.5:0.2, amine **2** (74 mg, 66% yield) was isolated as a white solid; *R*_*f*_ 0.50 (CH_2_Cl_2_/MeOH/50% aq. NH_4_OH 9:1:0.05).

A solution of **2** (54 mg, 0.13 mmol) in CH_2_Cl_2_ (5 mL) was filtered through a 0.2 μm PTFE filter and treated with a methanolic solution of HCl (0.53 N, 2.25 mL, 1.19 mmol). The resulting solution was evaporated at reduced pressure and the solid was washed with pentane (3 × 5 mL) to give, after drying under standard conditions, **2**·2HCl (54 mg) as a yellowish solid: mp 230–232 °C; IR (ATR) *ν* 3500–2500 (max at 3369, 2918, 2855, 2616, ^+^NH and CH st), 1633, 1595, 1578, 1560, 1518 (Ar–C–C and Ar–C–N st) cm^−1^; ^1^H NMR (400 MHz, CD_3_OD) *δ* 2.05 (m, 2H, 3-H_2_), 2.75 (t, *J* = 6.0 Hz, 2H, 4-H_2_), 3.64 (t, *J* = 4.8 Hz, 2H, 2-H_2_), 4.07 (s, 2H, 9-C*H*_2_-NH_2_), 4.85 (s, ^+^NH, ^+^NH_3_), 5.32 (s, 2H, 1-C*H*_2_-Ar), 7.39–7.53 (complex signal, 5H, 1-CH_2_-Ar–*H*), 7.66–7.71 (complex signal, 4H, 5-Ar–*H*), 7.94–8.01 (complex signal, 2H, 7-H, 8-H), 8.18 (br s, 1H, 10-H); ^13^C NMR (100.6 MHz, CD_3_OD) *δ* 21.4 (CH_2_, C3), 26.5 (CH_2_, C4), 44.2 (CH_2_, C2), 52.9 (CH_2_, 9-CH_2_-NH_2_), 61.7 (CH_2_, 1-*C*H_2_-Ar), 115.4 (C, C4a), 118.1 (C, C10a), 121.9 (CH, C7), 128.3 [2CH, 1-CH_2_-Ar–*C*2(6)], 128.5 (CH, 1-CH_2_-Ar–*C*4), 129.4 (CH, C10), 130.48 [2CH, 1-CH_2_-Ar–*C*3(5)], 130.50 [2CH, 5-Ar–*C*3(5)], 131.3 (C, C9), 131.9 [2CH, 5-Ar–*C*2(6)], 132.4 (C, 5-Ar–*C*4), 134.5 (CH, C8), 137.0 (C, 1-CH_2_-Ar–*C*1), 138.2 (C, 5-Ar–*C*1), 140.6 (C, C6a), 150.3 (C, C5), 159.7 (C, C10b); HRMS (ESI), calcd for [C_26_H_24_^3^^5^ClN_3_ + H^+^] 414.1732, found 414.1732; Elemental analysis, calcd for C_26_H_24_ClN_3_·2HCl·2.2H_2_O C 59.31%, H 5.82%, N 7.98%, found C 59.70%, H 5.71%, N 7.58%. HPLC purity: 97%.

#### 4.1.4 4-{1-Benzyl-9-chloro-1,2,3,4-tetrahydro-2-oxobenzo[h][1,6]naphthyridin-5-yl}benzonitrile **13**

It was prepared as described for **8**. From 4-chloroaniline, **10** (794 mg, 6.22 mmol), 4-formylbenzonitrile, **11** (816 mg, 6.22 mmol), Sc(OTf)_3_ (595 mg, 1.21 mmol), and enamine **4** (1.13 g, 6.04 mmol), a solid residue (2.71 g), mainly consisting of a diastereomeric mixture of octahydrobenzonaphthyridines **12**, was obtained and used in the next step without further purification.

From crude diastereomeric mixture **12** (2.71 g) and DDQ (3.65 g, 16.1 mmol), a solid residue (1.97 g) was obtained and purified through column chromatography (35–70 μm silica gel, hexane/EtOAc mixtures, gradient elution). On elution with hexane/EtOAc 75:25 to 60:40, compound **13** (886 mg, 35% yield) was isolated as a brown solid; *R*_*f*_ 0.46 (hexane/EtOAc 7:3).

A solution of **13** (396 mg, 0.93 mmol) in CH_2_Cl_2_ (10 mL) was filtered through a 0.2 μm NYL filter and evaporated at reduced pressure. The resulting solid was recrystallized from EtOAc (6 mL) and washed with pentane (3 × 5 mL) to give, after drying under standard conditions, the analytical sample of **13** (228 mg) as a beige solid: mp 252–254 °C; IR (KBr) *ν* 2228 (CN st), 1687, 1605, 1559, 1543 (C=O, Ar–C–C and Ar–C–N st) cm^−1^; ^1^H NMR (400 MHz, CDCl_3_) *δ* 2.64 (t, *J* = 6.8 Hz, 2H, 4′-H_2_), 2.90 (t, *J* = 6.8 Hz, 2H, 3′-H_2_), 5.33 (s, 2H, 1′-C*H*_2_–Ar), 7.13 [dm, *J* = 8.4 Hz, 2H, 1′–CH_2_–Ar–C2(6)-*H*], 7.22–7.31 (complex signal, 3H, 1′-CH_2_-Ar–C3(5)-*H*, 1′-CH_2_-Ar–C4–*H*), 7.64 (dd, *J* = 9.2 Hz, *J′* = 2.4 Hz, 1H, 8′-H), 7.71 (dm, *J* = 8.4 Hz, 2H) and 7.80 (dm, *J* = 8.4 Hz, 2H) [C2(6)-H, C3(5)-H], 7.92 (d, *J* = 2.4 Hz, 1H, 10′- H), 8.06 (d, *J* = 9.2 Hz, 1H, 7′-H); ^13^C NMR (100.6 MHz, CDCl_3_) *δ* 23.5 (CH_2_, C3′), 32.5 (CH_2_, C4′), 52.2 (CH_2_, 1′-*C*H2–Ar), 112.8 (C, C1), 118.4 (C, CN), 120.9 (C, C10a′), 122.1 (C, C4a′), 122.2 (CH), 127.2 (2 CH), 127.7 (CH), 128.6 (2 CH), 129.9 (2 CH), 130.6 (CH), 132.1 (CH), 132.3 (2 CH) (Ar–*C*H), 132.7 (C, C9′), 137.0 (C, 1′-CH_2_-Ar–*C*1), 143.5 (C), 146.4 (C), 146.9 (C) (C4, C6a′, C10b′), 156.2 (C, C5′), 172.3 (C, C2′); HRMS (ESI), calcd for [C_26_H_18_^35^ClN_3_O + H^+^] 424.1211, found 424.1213.

#### 4.1.5 4-{1-Benzyl-9-chloro-1,2,3,4-tetrahydrobenzo[h][1,6]naphthyridin-5-yl}benzylamine **3**

A solution of compound **13** (120 mg, 0.28 mmol) in anhydrous THF (10 mL) was treated with LiAlH_4_ (71 mg, 1.87 mmol). The reaction mixture was stirred under reflux overnight, cooled to 0 °C with an ice bath and treated dropwise with 1N NaOH (5 mL), then diluted with H_2_O (5 mL), and extracted with EtOAc (3 × 15 mL). The combined organic extracts were dried over anhydrous Na_2_SO_4_ and evaporated under reduced pressure to give a brown oily residue (210 mg), which was purified through column chromatography (35–70 μm silica gel, CH_2_Cl_2_/MeOH/50% aq. NH_4_OH mixtures, gradient elution). On elution with CH_2_Cl_2_/MeOH/50% aq. NH_4_OH 99:1:0.2 to 98:2:0.2, amine **3** (26 mg, 22% yield) was isolated as a beige solid; *R*_*f*_ 0.35 (CH_2_Cl_2_/MeOH/NH_4_OH 9.75:0.25:0.1).

A solution of **3** (26 mg, 0.06 mmol) in CH_2_Cl_2_ (40 mL) was filtered through a 0.2 μm NYL filter and treated with a methanolic solution of HCl (2.36 N, 0.12 mL, 0.28 mmol). The resulting solution was evaporated at reduced pressure and the solid was washed with pentane (3 × 2 mL) to give, after drying under standard conditions, **3**·2HCl (21 mg) as a beige solid: mp 171–173 °C; IR (ATR) *ν* 3500–2500 (max at 3382, 2921, 2850, ^+^NH, NH and CH st), 1633, 1623, 1615, 1575, 1567, 1559, 1539, 1534, 1515 (Ar–C–C and Ar–C–N st) cm^−1^; ^1^H NMR (400 MHz, CD_3_OD) *δ* 2.07 (m, 2H, 3′-H_2_), 2.76 (t, *J* = 6.0 Hz, 2H, 4′-H_2_), 3.68 (t, *J* = 5.6 Hz, 2H, 2′-H_2_), 4.30 (s, 2H, 1-C*H*_2_-NH_2_), 4.85 (s, ^+^NH, ^+^NH_3_) 5.20 (s, 2H, 1′-C*H*_2_–Ar), overimposed in part 7.44 (m, 1H, 1′-CH_2_-Ar–C4–*H*), 7.47 [d, *J* = 7.2 Hz, 2H, 1′-CH_2_-Ar–C2(6)-*H*], 7.54 [t, *J* = 7.2 Hz, 2H, 1′-CH_2_-Ar–C3(5)-*H*], 7.77–7.79 [complex signal, 4H, C2(6)-H, C3(5)-H], 7.82 (dd, *J* = 9.2 Hz, *J′* = 2.0 Hz, 1H, 8′-H), 7.90 (d, *J* = 9.2 Hz, 1H, 7′-H), 8.07 (d, *J* = 2.0 Hz, 1H, 10′-H); ^13^C NMR (100.6 MHz, CD_3_OD) *δ* 21.1 (CH_2_, C3′), 26.7 (CH_2_, C4′), 43.9 (CH_2_, 1-CH_2_-NH_2_), 53.4 (CH_2_, C2′), 61.4 (CH_2_, 1′-*C*H_2_–Ar), 115.4 (C, C4a′), 119.1 (C, C10a′), 122.8 (CH, C7′), 126.1 (CH, C10′), 127.5 [2 CH, 1′-CH_2_-Ar–*C*2(6)]), 129.4 (CH, 1′-CH_2_-Ar–*C*4), 130.6 [2 CH, 1′-CH_2_-Ar–*C*3(5)], 130.8 (2 CH) and 130.9 (2 CH) [C2(6), C3(5)], 132.1 (C, C9′), 134.5 (C, C4), 134.6 (CH, C8′), 136.7 (C, 1′-CH_2_-Ar–*C*1), 137.4 (C, C1), 139.0 (C, C6a′), 150.7 (C, C5′), 159.0 (C, C10b′); HRMS (ESI), calcd for [C_26_H_24_^35^ClN_3_ + H^+^] 414.1732, found 414.1743; Elemental analysis, calcd for C_26_H_24_ClN_3_·2HCl·0.75H_2_O C 62.41%, H 5.54%, found C 62.65%, H 5.70%. HPLC purity: 97%.

#### 4.1.6 4-{1-(tert-Butoxycarbonyl)-8-chloro-2,3-dihydro-1H-pyrrolo[3,2-c]quinolin-4-yl}benzonitrile **22**

It was prepared as described for **8**. From 4-chloroaniline, **10** (1.33 g, 10.4 mmol), 4-formylbenzonitrile, **11** (1.36 g, 10.4 mmol), Sc(OTf)_3_ (1.02 g, 2.07 mmol), and enamine **14** (1.80 mL, 1.76 g, 10.4 mmol), a sticky yellow solid residue (5.00 g), mainly consisting of a diastereomeric mixture of pyrroloquinolines **18**, was obtained and used in the next step without further purification.

From crude diastereomeric mixture **18** (5.00 g) and DDQ (4.72 g, 20.8 mmol), a solid residue (4.53 g) was obtained and purified through column chromatography (35–70 μm silica gel, hexane/EtOAc mixtures, gradient elution). On elution with hexane/EtOAc 70:30, compound **22** (2.47 g, 59% yield) was isolated as a grey solid; *R*_*f*_ 0.60 (hexane/EtOAc 1:1).

A solution of **22** (100 mg, 0.25 mmol) in CH_2_Cl_2_ (5 mL) was filtered through a 0.2 μm PTFE filter and diluted with hexane/EtOAc 2:1 (6 mL). The resulting solid precipitate was washed with pentane (3 × 5 mL) to give, after drying under standard conditions, the analytical sample of **22** (74 mg) as a grey solid: mp 210–211 °C; IR (ATR) *ν* 2229 (CN st), 1700 (C=O st), 1604, 1564, 1540 (Ar–C–C and Ar–C–N st) cm^−1^; ^1^H NMR (400 MHz, CDCl_3_) *δ* 1.59 [s, 9H, C(C*H*_3_)_3_], 3.31 (t, *J* = 8.0 Hz, 2H, 3′-H_2_), 4.30 (t, *J* = 8.0 Hz, 2H, 2′-H_2_), 7.61 (dd, *J* = 8.8 Hz, *J′* = 2.4 Hz, 1H, 7′-H), 7.80 (dm, *J* = 8.8 Hz, 2H) and 7.94 (dm, *J* = 8.8 Hz, 2H) [C2(6)-H, C3(5)-H], 8.05 (d, *J* = 8.8 Hz, 1H, 6′-H), 8.16 (d, *J* = 2.4 Hz, 1H, 9′-H); ^13^C NMR (100.6 MHz, CDCl_3_) *δ* 28.2 [3CH_3_, C(*C*H_3_)_3_], 28.8 (CH_2_, C3′), 51.5 (CH_2_, C2′), 82.9 [C, *C*(CH_3_)_3_], 112.5 (C, C1), 118.6 (C, CN), 119.6 (C), 124.4 (C) (C3a′, C9a′), 124.9 (CH), 130.3 (CH), 131.4 (CH) (C6′, C7′, C9′), 129.1 (2CH), 132.3 (2CH) [C2(6), C3(5)], 131.1 (C, C8′), 143.7 (C), 147.7 (C), 148.1 (C) (C4, C5a′, C9b′), 153.0 (C), 153.2 (C) (NCOO, C4′); HRMS (ESI), calcd for [C_23_H_20_^35^ClN_3_O_2_ + H^+^] 406.1317, found 406.1308.

#### 4.1.7 4-{1-(tert-Butoxycarbonyl)-9-chloro-1,2,3,4-tetrahydrobenzo[h][1,6]naphthyridin-5-yl}benzonitrile **23**

It was prepared as described for **8**. From 4-chloroaniline, **10** (1.24 g, 9.72 mmol), 4-formylbenzonitrile, **11** (1.27 g, 9.69 mmol), Sc(OTf)_3_ (0.95 g, 1.93 mmol), and enamine **15** (1.80 mL, 1.78 g, 9.70 mmol), a yellow solid residue (5.31 g), mainly consisting of a diastereomeric mixture of benzonaphthyridines **19**, was obtained and used in the next step without further purification.

From crude diastereomeric mixture **19** (5.31 g) and DDQ (4.40 g, 19.4 mmol), a brown residue (4.74 g) was obtained and purified through column chromatography (35–70 μm silica gel, hexane/EtOAc mixtures, gradient elution). On elution with hexane/EtOAc 90:10, compound **23** (2.79 g, 69% yield) was isolated as a white solid; *R*_*f*_ 0.60 (hexane/EtOAc 1:1).

A solution of **23** (71 mg, 0.17 mmol) in CH_2_Cl_2_ (8 mL) was filtered through a 0.2 μm PTFE filter and evaporated at reduced pressure. The resulting solid was washed with pentane (3 × 5 mL) to give, after drying under standard conditions, the analytical sample of **23** (70 mg) as a white solid: mp 146–147 °C; IR (ATR) *ν* 2222 (CN st), 1702 (C=O st), 1602, 1578, 1560 (Ar–C–C and Ar–C–N st) cm^−1^; ^1^H NMR (400 MHz, CDCl_3_) *δ* 1.40 [s, 9H, C(C*H*_3_)_3_], 1.99 (br signal, 2H, 3′-H_2_), 2.76 (t, *J* = 6.8 Hz, 2H, 4′-H_2_), 3.20–4.20 (br signal, 2H, 2′-H_2_), 7.58 (dd, *J* = 9.2 Hz, *J′* = 2.4 Hz, 1H, 8′-H), 7.69 (dm, *J* = 8.4 Hz, 2H), 7.79 (dm, *J* = 8.4 Hz, 2H) [C2(6)-H, C3(5)-H], superimposed 7.80 (m, 1H, 10′-H), 7.98 (d, *J* = 9.2 Hz, 1H, 7′-H); ^13^C NMR (100.6 MHz, CDCl_3_) *δ* 23.9 (CH_2_, C3′), 25.3 (CH_2_, C4′), 27.9 [3CH_3_, C(*C*H_3_)_3_)], 44.6 (CH_2_, C2′), 82.3 [C, *C*(CH_3_)_3_], 112.3 (C, C1), 118.6 (C, CN), 123.6 (CH + C), 124.5 (C), 129.8 (3CH), 131.1 (CH), 132.2 (2CH) [C2(6), C3(5), C4a′, C7′, C8′, C10′, C10a′), 131.8 (C, C9′), 144.1 (C), 144.4 (C), 145.4 (C) (C4, C6a′, C10b′), 153.8 (C, C5′), 157.7 (C, NCOO); HRMS (ESI), calcd for [C_24_H_22_^35^ClN_3_O_2_ + H^+^] 420.1473, found 420.1475.

#### 4.1.8 4-{1-(tert-Butoxycarbonyl)-10-chloro-2,3,4,5-tetrahydro-1H-azepino[3,2-c]quinolin-6-yl}benzonitrile **24**

It was prepared as described for **8**. From 4-chloroaniline, **10** (0.59 g, 4.62 mmol), 4-formylbenzonitrile, **11** (0.61 g, 4.65 mmol), Sc(OTf)_3_ (0.46 g, 0.93 mmol), and enamine **16** (0.90 mL, 924 mg, 4.69 mmol), a yellow solid residue (2.33 g), mainly consisting of a diastereomeric mixture of benzonaphthyridines **20**, was obtained and used in the next step without further purification.

From crude diastereomeric mixture **20** (2.33 g) and DDQ (2.11 g, 9.30 mmol), a solid residue (1.99 g) was obtained and purified through column chromatography (35–70 μm silica gel, hexane/EtOAc mixtures, gradient elution). On elution with hexane/EtOAc 80:20, compound **24** (1.58 g, 79% yield) was isolated as a white solid; *R*_*f*_ 0.58 (hexane/EtOAc 1:1).

A solution of **24** (200 mg, 0.46 mmol) in CH_2_Cl_2_ (10 mL) was filtered through a 0.2 μm PTFE filter and diluted with hexane/EtOAc 2:1 (3 mL). The solid precipitate was washed with pentane (3 × 5 mL) to give, after drying under standard conditions, the analytical sample of **24** (100 mg) as a white solid: mp 177–179 °C; IR (ATR) *ν* 2223 (CN st), 1698 (C=O st), 1602, 1579, 1564, 1550 (Ar–C–C and Ar–C–N st) cm^−1^; ^1^H NMR (400 MHz, CDCl_3_) *δ* 1.25 [s, C(C*H*_3_)_3_], 1.46 (m), 1.84–1.94 (complex signal), 1.98–2.16 (complex signal), 2.70 (tm, *J* = 14.0 Hz), 2.82 (tm, *J* = 14.0 Hz), 2.92 (ddm, *J* = 14.4 Hz, *J′* = 5.2 Hz), 4.40 (dm, *J* = 14.4 Hz, 2′-H_2_ minor rotamer), 4.58 (dt, *J* = 14.0 Hz, *J′* = 3.2 Hz, 2′-H_2_ major rotamer), superimposed in part 7.61 (dd, *J* = 9.2 Hz, *J′* = 2.4 Hz, 9′-H minor rotamer), 7.63 (dd, *J* = 9.2 Hz, *J′* = 2.4 Hz, 9′-H major rotamer), superimposed in part 7.65 [dm, *J* = 8.4 Hz, C2(6)-H or C3(5)-H minor rotamer], 7.70 (dm, *J* = 8.4 Hz), 7.80 dm, (*J* = 8.4 Hz) [C2(6)-H, C3(5)-H major rotamer], 7.78 (d, *J* = 2.4 Hz, 11′-H minor rotamer), superimposed in part 7.79 [dm, *J* = 8.4 Hz, C3(5)-H or C2(6)-H minor rotamer], 7.82 (d, *J* = 2.4 Hz, 11′-H major rotamer), 8.01 (d, *J* = 9.2 Hz, 8′-H minor rotamer) 8.03 (d, *J* = 9.2 Hz, 8′-H major rotamer); ^13^C NMR (100.6 MHz, CDCl_3_) *δ* significant signals of the major rotamer 24.8 (CH_2_, C4′), 28.0 [3CH_3_, C(*C*H_3_)_3_], 28.6 (CH_2_), 29.81 (CH_2_) (C3′, C5′), 47.3 (CH_2_, C2′), 81.2 [C, *C*(CH_3_)_3_], 112.3 (C, C1), 118.56 (C, CN), 122.2 (C), 125.5 (C) (C5a′, C11a′), 144.9 (C), 145.6 (C), 147.7 (C) (C4, C7a′, C11b′), 152.8 (C, C5′), 158.9 (C, NCOO); ^13^C NMR (100.6 MHz, CDCl_3_) *δ* significant signals of the minor rotamer 24.9 (CH_2_, C4′), 28.3 [3CH_3_, C(*C*H_3_)_3_], 29.4 (CH_2_), 29.75 (CH_2_) (C3′, C5′), 48.4 (CH_2_, C2′), 81.5 [C, *C*(CH_3_)_3_], 112.2 (C, C1), 118.59 (C, CN), 122.0 (C), 125.7 (C) (C5a’, C11a′), 145.0 (C), 145.8 (C), 147.9 (C) (C4, C7a′, C11b′), 153.5 (C, C5′), 159.0 (C, NCOO); HRMS (ESI), calcd for [C_25_H_24_^35^ClN_3_O_2_ + H^+^] 434.1630, found 434.1628.

#### 4.1.9 4-{1-(tert-Butoxycarbonyl)-8-chloro-1,2,3,4-tetrahydrobenzo[h][1,6]naphthyridin-5-yl}benzonitrile **25**

It was prepared as described for **8**. From 3-chloroaniline, **17** (1.03 mL, 1.25 g, 9.77 mmol), 4-formylbenzonitrile, **11** (1.27 g, 9.69 mmol), Sc(OTf)_3_ (0.95 g, 1.93 mmol), and enamine **15** (1.80 mL, 1.78 g, 9.70 mmol), a solid residue (4.90 g), mainly consisting of a diastereomeric mixture of benzonaphthyridines **21**, was obtained and used in the next step without further purification.

From crude diastereomeric mixture **21** (4.90 g) and DDQ (4.40 g, 19.4 mmol), a residue (4.42 g) was obtained and purified through column chromatography (35–70 μm silica gel, hexane/EtOAc mixtures, gradient elution). On elution with hexane/EtOAc 90:10, compound **25** (2.53 g, 62% yield) and 4-{3-[3-(*tert*-butoxycarbonylamino)propyl]-7-chloroquinolin-2-yl}benzonitrile, **34** (705 mg, 17% yield) were successively isolated; *R*_*f*__(**26**)_ 0.46; *R*_*f*__(**35**)_ 0.24 (hexane/EtOAc 70:30).

A solution of **25** (2.53 g, 6.04 mmol) in CH_2_Cl_2_ (25 mL) was filtered through a 0.2 μm PTFE filter and evaporated at reduced pressure. The resulting solid was washed with Et_2_O (3 × 10 mL) and pentane (3 × 8 mL) to give, after drying under standard conditions, the analytical sample of **25** (1.09 g) as a white solid: mp 162–164 °C; IR (ATR) *ν* 2223 (CN st), 1698 (C=O st), 1608, 1575, 1561 (Ar–C–C and Ar–C–N st) cm^−1^; ^1^H NMR (400 MHz, CDCl_3_) *δ* 1.41 [s, 9H, C(C*H*_3_)_3_], 1.99 (br signal, 2H, 3′-H_2_), 2.75 (t, *J* = 6.8 Hz, 2H, 4′-H_2_), 3.4–4.4 (br signal, 2H, 2′-H_2_), 7.47 (dd, *J* = 8.8 Hz, *J′* = 2.0 Hz, 1H, 9′-H), 7.69 (dm, *J* = 8.4 Hz, 2H), 7.79 (dm, *J* = 8.4 Hz, 2H) [C2(6)-H, C3(5)-H], 7.74 (d, *J* = 8.8 Hz, 10′-H), 8.04 (d, *J* = 2.0 Hz, 1H, 7′-H); ^13^C NMR (100.6 MHz, CDCl_3_) *δ* 23.9 (CH_2_, C3′), 25.2 (CH_2_, C4′), 28.0 [3CH_3_, C(*C*H_3_)_3_], 44.8 (CH_2_, C2′), 82.1 [C, *C*(CH_3_)_3_], 112.3 (C, C1), 118.6 (C, CN), 122.3 (C), 123.0 (C) (C4a′, C10a′), 125.8 (CH), 126.7 (CH), 128.3 (CH) (C7′, C9′, C10′), 129.7 (2CH), 132.2 (2CH) [C2(6), C3(5)], 134.8 (C, C8′), 144.4 (C), 145.0 (C), 147.4 (C) (C4, C6a′, C10b′), 153.9 (C, C5′), 158.5 (C, NCOO); HRMS (ESI), calcd for [C_24_H_22_^35^ClN_3_O_2_ + H^+^] 420.1473, found 420.1469.

A solution of **34** (30 mg, 0.07 mmol) in CH_2_Cl_2_ (2 mL) was filtered through a 0.2 μm PTFE filter and evaporated at reduced pressure. The resulting solid was washed with Et_2_O (3 × 5 mL) and pentane (3 × 5 mL) to give, after drying under standard conditions, the analytical sample of **34** (19 mg) as a yellow solid: mp 124–126 °C; IR (ATR) *ν* 3358 (NH st), 2229 (CN st), 1680 (C=O st), 1623, 1591, 1519 (Ar–C–C and Ar–C–N st) cm^−1^; ^1^H NMR (400 MHz, CDCl_3_) *δ* 1.41 [s, 9H, C(C*H*_3_)_3_], 1.71 (tt, *J* = 7.6 Hz, *J′*=6.4 Hz, 2H, 2″-H_2_), 2.78 (t, *J* = 7.6 Hz, 2H, 1″-H_2_), 3.06 (dt, *J* = *J′* = 6.4 Hz, 2H, 3″-H_2_), 4.42 (br s, 2H, NHCOO), 7.52 (dd, *J* = 9.2 Hz, *J′* = 2.0 Hz, 1H, 6′-H), 7.67 (dm, *J* = 8.4 Hz, 2H), 7.80 (dm, *J* = 8.4 Hz, 2H) [C2(6)-H, C3(5)-H], 7.76 (d, *J* = 9.2 Hz, 1H, 5′-H), 8.08 (br s, 1H, 4′-H), 8.09 (d, *J* = 2.0 Hz, 1H, 8′-H); ^13^C NMR (100.6 MHz, CDCl_3_) *δ* 28.4 [3CH_3_, C(*C*H_3_)_3_], 29.9 (CH_2_), 31.0 (CH_2_) (C1″, C2″), 39.9 (CH_2_, C3″), 79.4 [C, *C*(CH_3_)_3_], 112.4 (C, C1), 118.5 (C, CN), 126.1 (C, C4a′), 128.2 (2CH), 128.3 (CH), 129.6 (2CH), 132.3 (2CH) [C2(6), C3(5), C5′, C6′, C8′], 132.7 (C), 135.3 (C) (C7′, C8a′), 136.2 (CH, C4′), 144.8 (C), 146.8 (C) (C4, C3′), 155.8 [C, C2′], 159.2 (C, NHCOO); HRMS (ESI), calcd for [C_24_H_24_^35^ClN_3_O_2_ + H^+^] 422.1630, found 422.1623.

#### 4.1.10 4-{8-Chloro-2,3-dihydro-1H-pyrrolo[3,2-c]quinolin-4-yl}benzonitrile **26**

It was prepared as described for **9**. From **22** (877 mg, 2.16 mmol) and 4M HCl/dioxane solution (18 mL), a solid residue (430 mg) was obtained. Recrystallization from EtOAc/hexane 1:1.5 afforded nitrile **26** (307 mg, 46% yield) as a orange solid; *R*_*f*_ 0.30 (hexane/EtOAc 1:1).

A solution of **26** (100 mg, 0.33 mmol) in CH_2_Cl_2_ (10 mL) was filtered through a 0.2 μm PTFE filter and treated with a methanolic solution of HCl (0.53 N, 1.85 mL, 0.98 mmol). The resulting solution was evaporated at reduced pressure and the solid was washed with pentane (3 × 5 mL) to give, after drying under standard conditions, **26**·HCl (108 mg) as a yellow solid: mp 324–326 °C; IR (ATR) *ν* 3500–2500 (max at 3359, 3183, 3023, 2925, 2749, ^+^NH, NH and CH st), 2228 (CN st), 1633, 1604, 1588, 1556, 1519 (Ar–C–C and Ar–C–N st) cm^−1^; ^1^H NMR (400 MHz, CD_3_OD) *δ* 3.42 (t, *J* = 9.0 Hz, 2H, 3′-H_2_), 4.18 (t, *J* = 9.0 Hz, 2H, 2′-H_2_), 4.85 (s, ^+^NH, NH), 7.90–7.92 (complex signal, 2H, 6′-H, 7′-H), 7.97 [br d, *J* = 8.4 Hz, 2H, C3(5)-H], 8.02 [br d, *J* = 8.4 Hz, 2H, C2(6)-H], 8.21 (m, 1H, 9′-H); ^13^C NMR (100.6 MHz, CD_3_OD) *δ* 27.1 (CH_2_, C3′), 49.4 (CH_2_, C2′), 114.0 (C, C3a′), 116.0 (C, C1), 118.8 (C, CN), 119.7 (C, C9a′), 122.7 (CH), 124.6 (CH) (C6′, C9′), 130.7 [2CH, C3(5)], 133.0 (C, C8′), 134.2 [2CH, C2(6)], 136.2 (CH, C7′), 137.5 (C, C5a′), 140.1 (C, C4), 146.0 (C, C4′), 162.6 (C, C9b′); HRMS (ESI), calcd for [C_18_H_12_^35^ClN_3_ + H^+^] 306.0793, found 306.0792; Elemental analysis, calcd for C_18_H_12_ClN_3_·HCl·1/2H_2_O C 61.55%, H 4.02%, N 11.96%, found C 61.35%, H 4.07%, N 11.73%. HPLC purity: 94%.

#### 4.1.11 4-{9-Chloro-1,2,3,4-tetrahydrobenzo[h][1,6]naphthyridin-5-yl}benzonitrile **27**

It was prepared as described for **9**. From **23** (1.76 g, 4.20 mmol) and 4M HCl/dioxane solution (29 mL), nitrile **27** (915 mg, 68% yield) was obtained as a yellowish solid; *R*_*f*_ 0.60 (hexane/EtOAc 1:1).

A solution of **27** (154 mg, 0.48 mmol) in CH_2_Cl_2_ (10 mL) was filtered through a 0.2 μm PTFE filter and treated with a methanolic solution of HCl (0.53 N, 2.73 mL, 1.45 mmol). The resulting solution was evaporated at reduced pressure and the solid was washed with pentane (3 × 5 mL) to give, after drying under standard conditions, **27**·HCl (148 mg) as a beige solid: mp 343–344 °C; IR (ATR) *ν* 3500–2500 (max at 3170, 3077, 2708, ^+^NH, NH and CH st), 2226 (CN st), 1627, 1584, 1566 (Ar–C–C and Ar–C–N st) cm^−1^; ^1^H NMR (400 MHz, CD_3_OD) *δ* 1.98 (tt, *J* = 6.0 Hz, *J′* = 5.6 Hz, 2H, 3′-H_2_), 2.71 (t, *J* = 6.0 Hz, 2H, 4′-H_2_), 3.69 (t, *J* = 5.6 Hz, 2H, 2′-H_2_), 4.85 (s, ^+^NH, NH), 7.84 (d, *J* = 8.8 Hz, 1H, 7′-H), 7.85 [br d, *J* = 8.4 Hz, 2H, C3(5)-H], 7.90 (dd, *J* = 8.8 Hz, *J′* = 2.0 Hz, 1H, 8′-H), 8.02 [br d, *J* = 8.4 Hz, 2H, C2(6)-H], 8.41 (d, *J* = 2.0 Hz, 1H, 10′-H); ^13^C NMR (100.6 MHz, CD_3_OD) *δ* 19.9 (CH_2_, C3′), 24.9 (CH_2_, C4′), 43.0 (CH_2_, C2′), 109.8 (C, C4a′), 115.8 (C, C1), 117.8 (C, C10a′), 118.9 (C, CN), 122.8 (CH), 122.9 (CH) (C7′, C10′), 131.3 [2CH, C3(5)], 133.6 (C, C9′), 134.0 [2CH, C2(6)], 135.1 (CH, C8′), 137.4 (C, C6a′), 138.0 (C, C4), 150.3 (C, C5′), 154.7 (C, C10b′); HRMS (ESI), calcd for [C_19_H_14_^35^ClN_3_ + H^+^] 320.0949, found 320.0948; Elemental analysis, calcd for C_19_H_14_ClN_3_·HCl C 64.06%, H 4.24%, N 11.80%, found C 63.79%, H 4.22%, N 11.79%. HPLC purity >99%.

#### 4.1.12 4-{10-Chloro-2,3,4,5-tetrahydro-1H-azepino[3,2-c]quinolin-6-yl}benzonitrile **28**

It was prepared as described for **9**. From **24** (997 mg, 2.30 mmol) and 4M HCl/dioxane solution (16 mL), nitrile **28** (392 mg, 51% yield) was obtained as a beige solid; *R*_*f*_ 0.50 (hexane/EtOAc 1:1).

A solution of **28** (392 mg, 1.17 mmol) in CH_2_Cl_2_ (8 mL) was filtered through a 0.2 μm PTFE filter and diluted with hexane/EtOAc 2:1 (3 mL). The solid precipitate (aliquot of 111 mg of a total amount of 232 mg) was treated with a methanolic solution of HCl (0.53 N, 1.88 mL, 1.00 mmol). The resulting solution was evaporated at reduced pressure and the solid was washed with pentane (3 × 5 mL) to give, after drying under standard conditions, **28**·HCl (114 mg) as a yellowish solid: mp 338–340 °C; IR (ATR) *ν* 3500–2500 (max at 3167, 3085, 3033, 2930, 2795, 2715, 2635, ^+^NH, NH and CH st), 2222 (CN st), 1627, 1599, 1573, 1560, 1501 (Ar–C–C and Ar–C–N st) cm^−1^; ^1^H NMR (400 MHz, CD_3_OD) *δ* 2.04 (m, 2H, 4′-H_2_), 2.19 (m, 2H, 3′-H_2_), 2.85 (m, 2H, 5′-H_2_), 3.96 (m, 2H, 2′-H_2_), 4.85 (s, ^+^NH, NH), superimposed in part 7.82 (d, *J* = 8.8 Hz, 1H, 8′-H), 7.84 [br d, *J* = 8.4 Hz, 2H, C3(5)-H], 7.89 (dd, *J* = 8.8 Hz, *J′* = 2.4 Hz, 1H, 9′-H), 8.02 [br d, *J* = 8.4 Hz, 2H, C2(6)-H], 8.43 (d, *J* = 2.4 Hz, 1H, 11′-H); ^13^C NMR (100.6 MHz, CD_3_OD) *δ* 26.3 (CH_2_, C4′), 27.7 (CH_2_, C3′), 27.9 (CH_2_, C5′), 44.9 (CH_2_, C2′), 114.7 (C, C5a’), 115.9 (C, C1), 118.8 (C, CN), 119.3 (C, C11a′), 122.7 (CH, C8′), 123.0 (CH, C11′), 131.4 [2CH, C3(5)], 133.8 (C, C10′), 134.0 [2CH, C2(6)], 135.1 (CH, C9′), 137.3 (C, C7a′), 138.8 (C, C4), 152.6 (C, C6′), 160.0 (C, C11b′); HRMS (ESI), calcd for [C_20_H_16_^35^ClN_3_ + H^+^] 334.1105, found 334.1106; Elemental analysis, calcd for C_20_H_16_ClN_3_·HCl·0.3H_2_O C 63.94%, H 4.72%, N 11.18%, found C 63.72%, H 4.56%, N 10.85%. HPLC purity >99%.

#### 4.1.13 4-{8-Chloro-1,2,3,4-tetrahydrobenzo[h][1,6]naphthyridin-5-yl}benzonitrile **29**

It was prepared as described for **9**. From **25** (2.24 g, 5.34 mmol) and 4M HCl/dioxane solution (37 mL), nitrile **29** (1.60 g, 94% yield) was obtained as a yellow solid; *R*_*f*_ 0.34 (hexane/EtOAc 1:1).

A solution of **29** (275 mg, 0.86 mmol) in CH_2_Cl_2_ (15 mL) was filtered through a 0.2 μm PTFE filter and treated with a methanolic solution of HCl (0.53 N, 5.16 mL, 2.73 mmol). The resulting solution was evaporated at reduced pressure and the solid was recrystallized from MeOH/hexane 1:3.75 (1.9 mL) and washed with pentane (3 × 8 mL) to give, after drying under standard conditions, **29**·HCl (166 mg) as a brown solid: mp 360–362 °C; IR (ATR) *ν* 3500–2500 (max at 3148, 3078, 3035, 2905, 2734, ^+^NH, NH and CH st), 2228 (CN st), 1629, 1583, 1500 (Ar–C–C and Ar–C–N st) cm^−1^; ^1^H NMR (400 MHz, CD_3_OD) *δ* 1.98 (tt, *J* = 6.0 Hz, *J′* = 5.2 Hz, 2H, 3′-H_2_), 2.70 (t, *J* = 6.0 Hz, 2H, 4′-H_2_), 3.69 (t, *J* = 5.2 Hz, 2H, 2′-H_2_), 4.85 (s, ^+^NH, NH), 7.67 (dd, *J* = 8.8 Hz, *J′* = 1.6 Hz, 1H, 9′-H), superimposed in part 7.84 (m, 1H, 7′-H), 7.85 (br d, *J* = 7.6 Hz, 2H), 8.02 (br d, *J* = 7.6 Hz, 2H) [C2(6)-H, C3(5)-H], 8.29 (d, *J* = 8.8 Hz, 1H, 10′-H); ^13^C NMR (100.6 MHz, CD_3_OD) *δ* 20.0 (CH_2_, C3′), 24.9 (CH_2_, C4′), 43.1 (CH_2_, C2′), 109.7 (C, C4a′), 115.4 (C), 115.9 (C) (C1, C10a′), 118.9 (C, CN), 120.0 (CH), 125.5 (CH), 128.4 (CH) (C7′, C9′, C10′), 131.3 (2CH), 134.0 (2CH) [C2(6), C3(5)], 138.0 (C), 139.6 (C), 140.8 (C) (C4, C6a′, C8′), 150.5 (C, C5′), 155.4 (C, C10b′); HRMS (ESI), calcd for [C_19_H_14_^35^ClN_3_ + H^+^] 320.0949, found 320.0946; Elemental analysis, calcd for C_19_H_14_ClN_3_·HCl C 64.06%, H 4.24%, N 11.80%, found C 64.02%, H 4.41%, N 11.85%. HPLC purity >99%.

#### 4.1.14 4-{8-Chloro-2,3-dihydro-1H-pyrrolo[3,2-c]quinolin-4-yl}benzylamine **30**

It was prepared as described for **3**. From nitrile **26** (216 mg, 0.71 mmol) and LiAlH_4_ (0.13 g, 3.43 mmol), a yellow solid residue (210 mg) was obtained and purified by column chromatography (35–70 μm silica gel, CH_2_Cl_2_/MeOH/50% aq. NH_4_OH mixtures, gradient elution). On elution with CH_2_Cl_2_/MeOH/50% aq. NH_4_OH 99:1:0.2 to 98.5:1.5:0.2, the amine **30** (163 mg, 74% yield) was isolated as a beige solid; *R*_*f*_ 0.22 (CH_2_Cl_2_/MeOH/50% aq. NH_4_OH 9:1:0.05).

A solution of **30** (138 mg, 0.45 mmol) in CH_2_Cl_2_ (10 mL) was filtered through a 0.2 μm PTFE filter and treated with a methanolic solution of HCl (0.53 N, 7.54 mL, 4.00 mmol). The resulting solution was evaporated at reduced pressure and the solid was recrystallized from MeOH/EtOAc 1:3 (4 mL), and then washed with pentane (3 × 5 mL) to give, after drying under standard conditions, **30**·2HCl (108 mg) as a yellow solid: mp 319–320 °C; IR (ATR) *ν* 3500–2500 (max at 3364, 3111, 3043, 2943, 2614, ^+^NH, NH and CH st), 1638, 1605, 1573, 1529, 1511 (Ar–C–C and Ar–C–N st) cm^−1^; ^1^H NMR (400 MHz, CD_3_OD) *δ* 3.42 (t, *J* = 8.8 Hz, 2H, 3′-H_2_), 4.17 (t, *J* = 8.8 Hz, 2H, 2′-H_2_), 4.29 (s, 2H, 1-C*H*_2_-NH_2_), 4.85 (s, ^+^NH, ^+^NH_3_, NH), 7.78 (br d, *J* = 8.4 Hz, 2H), 7.90 (br d, *J* = 8.4 Hz, 2H) [C2(6)-H, C3(5)-H], 7.86 (dd, *J* = 9.2 Hz, *J′* = 2.4 Hz, 1H, 7′-H), 7.95 (d, *J* = 9.2 Hz, 1H, 6′-H), 8.19 (m, 1H, 9′-H); ^13^C NMR (100.6 MHz, CD_3_OD) *δ* 27.4 (CH_2_, C3′), 43.8 (CH_2_, 1-CH_2_-NH_2_), 49.2 (CH_2_, C2′), 113.9 (C, C3a’), 119.1 (C, C9a′), 122.7 (CH, C6′), 124.6 (CH, C9′), 130.4 (2CH), 131.0 (2CH) [C2(6), C3(5)], 132.7 (C, C4), 133.8 (C, C8′), 135.9 (CH, C7′), 137.7 (C, C1), 140.0 (C, C5a′), 147.2 (C, C4′), 162.4 (C, C9b′); HRMS (ESI), calcd for [C_18_H_16_^35^ClN_3_ + H^+^] 310.1106, found 310.1101; Elemental analysis, calcd for C_18_H_16_ClN_3_·2HCl·1.5H_2_O C 52.76%, H 5.17%, N 10.26%, found C 52.63%, H 5.21%, N 9.91%. HPLC purity >99%.

#### 4.1.15 4-{9-Chloro-1,2,3,4-tetrahydrobenzo[h][1,6]naphthyridin-5-yl}benzylamine **31**

It was prepared as described for **3**. From nitrile **27** (720 mg, 2.25 mmol) and LiAlH_4_ (0.43 g, 11.3 mmol), a white solid residue (892 mg) was obtained and purified by column chromatography (35–70 μm silica gel, CH_2_Cl_2_/MeOH/50% aq. NH_4_OH mixtures, gradient elution). On elution with CH_2_Cl_2_/MeOH/50% aq. NH_4_OH 97:3:0.2 to 95:5:0.2, the amine **31** (632 mg, 87% yield) was isolated as a white solid; *R*_*f*_ 0.18 (CH_2_Cl_2_/MeOH/50% aq. NH_4_OH 9:1:0.05).

A solution of **31** (632 mg, 1.95 mmol) in CH_2_Cl_2_ (10 mL) was filtered through a 0.2 μm PTFE filter and treated with a methanolic solution of HCl (0.53 N, 33 mL, 17.5 mmol). The resulting solution was evaporated at reduced pressure and the solid was washed with pentane (3 × 10 mL) to give, after drying under standard conditions, **31**·2HCl (640 mg) as a beige solid: mp 346–347 °C; IR (ATR) *ν* 3500–2500 (max at 3028, 2987, 2941, ^+^NH, NH and CH st), 1612, 1573, 1511 (Ar–C–C and Ar–C–N st) cm^−1^; ^1^H NMR (400 MHz, CD_3_OD) *δ* 1.97 (tt, *J* = 6.4 Hz, *J′* = 5.2 Hz, 2H, 3′-H_2_), 2.74 (t, *J* = 6.4 Hz, 2H, 4′-H_2_), 3.69 (t, *J* = 5.2 Hz, 2H, 2′-H_2_), 4.30 (s, 2H, 1-C*H*_2_-NH_2_), 4.86 (s, ^+^NH, ^+^NH_3_, NH), 7.75 (br d, *J* = 8.4 Hz, 2H), 7.78 (br d, *J* = 8.4 Hz, 2H) [C2(6)-H, C3(5)-H], 7.86 (dd, *J* = 9.2 Hz, *J′* = 2.0 Hz, 1H, 8′-H), 7.89 (d, *J* = 9.2 Hz, 1H, 7′-H), 8.41 (d, *J* = 2.0 Hz, 1H, 10′-H); ^13^C NMR (100.6 MHz, CD_3_OD) *δ* 20.0 (CH_2_, C3′), 25.1 (CH_2_, C4′), 42.9 (CH_2_, C2′), 43.9 (CH_2_, 1-CH_2_-NH_2_), 109.7 (C, C4a′), 117.7 (C, C10a′), 122.8 (CH, C7′), 122.9 (CH, C10′), 130.7 (2CH), 130.9 (2CH) [C2(6), C3(5)], 133.4 (C, C9′), 134.3 (C, C4), 134.8 (CH, C8′), 137.4 (2C, C1, C6a′), 151.6 (C, C5′), 154.5 (C, C10b′); HRMS (ESI), calcd for [C_19_H_18_^35^ClN_3_ + H^+^] 324.1262, found 324.1263; Elemental analysis, calcd for C_19_H_18_ClN_3_·2HCl·1/2H_2_O C 56.24%, H 5.22%, N 10.36%, found C 56.44%, H 5.25%, N 10.14%. HPLC purity >99%.

#### 4.1.16 4-{10-Chloro-2,3,4,5-tetrahydro-1H-azepino[3,2-c]quinolin-6-yl}benzylamine **32**

It was prepared as described for **3**. From nitrile **28** (172 mg, 0.52 mmol) and LiAlH_4_ (0.10 g, 2.64 mmol), a yellow solid residue (363 mg) was obtained and purified by column chromatography (35–70 μm silica gel, CH_2_Cl_2_/MeOH/50% aq. NH_4_OH mixtures, gradient elution). On elution with CH_2_Cl_2_/MeOH/50% aq. NH_4_OH 99:1:0.2, impure amine **32** (189 mg) was obtained. Recrystallization from hexane/EtOAc 2:1 (3 mL) afforded pure amine **32** (97 mg, 55% yield) as a white solid; *R*_*f*_ 0.20 (CH_2_Cl_2_/MeOH/50% aq. NH_4_OH 9:1:0.05).

A solution of **32** (97 mg, 0.29 mmol) in CH_2_Cl_2_ (5 mL) was filtered through a 0.2 μm PTFE filter and treated with a methanolic solution of HCl (0.53 N, 4.88 mL, 2.59 mmol). The resulting solution was evaporated at reduced pressure and the solid was washed with pentane (3 × 5 mL) to give, after drying under standard conditions, **32**·2HCl (98 mg) as a yellowish solid: mp 310–311 °C; IR (ATR) *ν* 3500–2500 (max at 3379, 3219, 3028, 2936, ^+^NH, NH and CH st), 1627, 1586, 1511 (Ar–C–C and Ar–C–N st) cm^−1^; ^1^H NMR (400 MHz, CD_3_OD) *δ* 2.02 (m, 2H, 4′-H_2_), 2.18 (m, 2H, 3′-H_2_), 2.88 (m, 2H, 5′-H_2_), 3.96 (m, 2H, 2′-H_2_), 4.30 (s, 2H, 1-C*H*_2_-NH_2_), 4.86 (s, ^+^NH, ^+^NH_3_, NH), 7.74 (br d, *J* = 8.4 Hz, 2H), 7.78 (br d, *J* = 8.4 Hz, 2H) [C2(6)-H, C3(5)-H], 7.84–7.90 (complex signal, 2H, 8′-H, 9′-H), 8.44 (m, 1H, 11′-H); ^13^C NMR (100.6 MHz, CD_3_OD) *δ* 26.4 (CH_2_, C4′), 27.8 (CH_2_, C3′), 28.0 (CH_2_, C5′), 43.9 (CH_2_, 1-CH_2_-NH_2_), 44.9 (CH_2_, C2′), 114.7 (C, C5a′), 119.2 (C, C11a′), 122.7 (CH, C8′), 123.0 (CH, C11′), 130.8 (2CH), 131.1 (2CH) [C2(6), C3(5)], 133.6 (C, C10′), 134.8 (CH, C9′), 135.1 (C, C4), 137.3 (C) and 137.5 (C) (C1, C7a′), 153.9 (C, C6′), 159.8 (C, C11b′); HRMS (ESI), calcd for [C_20_H_20_^35^ClN_3_ + H^+^] 338.1419, found 338.1416; Elemental analysis, calcd for C_20_H_20_ClN_3_·2HCl·2.5H_2_O C 52.70%, H 5.97%, N 9.22%, found C 53.02%, H 5.69%, N 8.72%. HPLC purity >99%.

#### 4.1.17 4-{8-Chloro-1,2,3,4-tetrahydrobenzo[h][1,6]naphthyridin-5-yl}benzylamine **33**

It was prepared as described for **3**. From nitrile **29** (1.17 g, 3.66 mmol) and LiAlH_4_ (0.69 g, 18.2 mmol), a yellow solid residue (3.07 g) was obtained. An aliquot of this crude product (1.50 g) was purified by column chromatography (35–70 μm silica gel, EtOAc/MeOH/50% aq. NH_4_OH mixtures, gradient elution). On elution with EtOAc/MeOH/50% aq. NH_4_OH 98:2:0.2, the amine **33** (390 mg, 67% yield) was isolated; *R*_*f*_ 0.29 (EtOAc/MeOH/50% aq. NH_4_OH 7:3:0.05).

A solution of **33** (390 mg, 1.20 mmol) in CH_2_Cl_2_ (20 mL) was filtered through a 0.2 μm PTFE filter and treated with a methanolic solution of HCl (0.53 N, 20.4 mL, 10.8 mmol). The resulting solution was evaporated at reduced pressure and the solid was washed with pentane (3 × 15 mL) to give, after drying under standard conditions, **33**·2HCl (400 mg) as a yellowish solid: mp 305–307 °C; IR (ATR) *ν* 3500–2500 (max at 3369, 3212, 2926, 2869, 2614, ^+^NH, NH and CH st), 1625, 1583, 1516 (Ar–C–C and Ar–C–N st) cm^−1^; ^1^H NMR (400 MHz, CD_3_OD) *δ* 1.97 (tt, *J* = 6.0 Hz, *J′* = 5.6 Hz, 2H, 3′-H_2_), 2.74 (t, *J* = 6.0 Hz, 2H, 4′-H_2_), 3.70 (t, *J* = 5.6 Hz, 2H, 2′-H_2_), 4.30 (s, 2H, 1-C*H*_2_-NH_2_), 4.85 (s, ^+^NH, ^+^NH_3_, NH), 7.63 (dd, *J* = 9.2 Hz, *J′* = 2.0 Hz, 1H, 9′-H), 7.75 (br d, *J* = 8.4 Hz, 2H), 7.78 (br d, *J* = 8.4 Hz, 2H) [C2(6)-H, C3(5)-H], 7.90 (d, *J* = 2.0 Hz, 1H, 7′-H), 8.32 (d, *J* = 9.2 Hz, 1H, 10′-H); ^13^C NMR (100.6 MHz, CD_3_OD) *δ* 20.0 (CH_2_, C3′), 25.0 (CH_2_, C4′), 43.0 (CH_2_, C2′), 43.9 (CH_2_, 1-CH_2_-NH_2_), 109.5 (C, C4a′), 115.3 (C, C10a′), 119.9 (CH), 125.5 (CH), 128.2 (CH) (C7′, C9′, C10′), 130.8 (2CH), 130.9 (2CH) [C2(6), C3(5)], 134.3 (C), 137.4 (C), 139.5 (C), 140.5 (C) (C1, C4, C6a′, C8′), 151.7 (C, C5′), 155.1 (C, C10b′); HRMS (ESI), calcd for [C_19_H_18_^35^ClN_3_ + H^+^] 324.1262, found 324.1258; Elemental analysis, calcd for C_19_H_18_ClN_3_·2HCl·1.8H_2_O C 53.17%, H 5.54%, N 9.79%, found C 53.56%, H 5.54%, N 9.34%. HPLC purity: 98%.

#### 4.1.18 4-[3-(3-Aminopropyl)-7-chloroquinolin-2-yl]benzonitrile **35**

It was prepared as described for **9**. From **34** (705 mg, 1.67 mmol) and 4M HCl/dioxane solution (12 mL), amino nitrile **35** (486 mg, 90% yield) was obtained; *R*_*f*_ 0.17 (EtOAc/MeOH/50% aq. NH_4_OH 7:3:0.05).

A solution of **35** (109 mg, 0.34 mmol) in CH_2_Cl_2_ (8 mL) was filtered through a 0.2 μm PTFE filter and treated with a methanolic solution of HCl (0.80 N, 1.27 mL, 1.02 mmol). The resulting solution was evaporated at reduced pressure and the solid was recrystallized from MeOH/EtOAc 1:4 (3 mL) and washed with pentane (3 × 5 mL) to give, after drying under standard conditions, **35**·2HCl (70 mg) as a red solid: mp 153–154 °C; IR (ATR) *ν* 3500–2500 (max at 3369, 2954, 2609, ^+^NH, NH and CH st), 2226 (CN st), 1698, 1639, 1607, 1591, 1555, 1534, 1500 (Ar–C–C and Ar–C–N st) cm^−1^; ^1^H NMR (400 MHz, CD_3_OD) *δ* 1.99 (tt, *J* = 8.0 Hz, *J′* = 7.6 Hz, 2H, 2″-H_2_), superimposed in part 2.93 (t, *J* = 7.6 Hz, 2H), 2.97 (t, *J* = 8.0 Hz, 2H) (1″-H_2_, 3″-H_2_), 4.88 (s, ^+^NH_3_, ^+^NH), 7.92 (dd, *J* = 8.8 Hz, *J′* = 2.0 Hz, 1H, 6′-H), 7.96 (br d, *J* = 8.0 Hz, 2H), 8.07 (br d, *J* = 8.0 Hz, 2H) [C2(6)-H, C3(5)-H], 8.23 (d, *J* = 2.0 Hz, 1H, 8′-H), 8.32 (d, *J* = 8.8 Hz, 1H, 5′-H), 9.10 (s, 1H, 4′-H); ^13^C NMR (100.6 MHz, CD_3_OD) *δ* 28.7 (CH_2_), 29.8 (CH_2_) (C1″, C2″), 40.0 (CH_2_, C3″), 116.0 (C, C1), 118.9 (C, CN), 122.4 (CH), 131.46 (CH), 131.8 (CH) (C5′, C6′, C8′), 128.6 (C, C4a′), 131.51 (2CH), 134.09 (2CH) [C2(6), C3(5)], 135.4 (C), 138.7 (C), 141.0 (C), 141.1 (C) (C4, C3′, C7′, C8a′), 145.8 (CH, C4′), 157.6 (C, C2′); HRMS (ESI), calcd for [C_19_H_16_^35^ClN_3_ + H^+^] 322.1106, found 322.1103; Elemental analysis, calcd for C_19_H_16_ClN_3_·2HCl·1.2H_2_O C 54.81%, H 4.94%, N 10.09%, found C 55.02%, H 4.75%, N 9.61%. HPLC purity: 94%.

#### 4.1.19 4-{9-Chloro-3,4-dihydro-2H-pyrano[3,2-c]quinolin-5-yl}benzonitrile **38**

It was prepared as described for **8**. From 4-chloroaniline, **10** (1.51 g, 11.8 mmol), 4-formylbenzonitrile, **11** (1.56 g, 11.9 mmol), Sc(OTf)_3_ (1.18 g, 2.40 mmol), and 3,4-dihydro-2*H*-pyran, **36** (1.08 mL, 994 mg, 11.8 mmol), a solid residue (4.72 g) was obtained and purified by column chromatography (35–70 μm silica gel, hexane/EtOAc mixtures, gradient elution). On elution with hexane/EtOAc 80:20 to 50:50, a 1:1 diastereomeric mixture **37** (3.65 g, 95% yield) was isolated as a white solid. After a second column chromatography separation of 1.00 g of diastereomeric mixture **37** (35–70 μm silica gel, hexane/EtOAc mixtures, gradient elution), pure all-*cis*-diatereoisomer (90 mg) was isolated as a white solid; *R*_*f*_ 0.43 (hexane/EtOAc 7:3).

A solution of all-*cis*-**37** (90 mg, 0.28 mmol) in CH_2_Cl_2_ (5 mL) was filtered through a 0.2 μm PTFE filter and evaporated at reduced pressure. The resulting solid was washed with pentane (3 × 5 mL) to give, after drying under standard conditions, the analytical sample of all-*cis*-**37** (85 mg) as a white solid: mp 284–285 °C; IR (ATR) *ν* 3348 (NH st), 2225 (CN st), 1604, 1575 (Ar–C–C and Ar–C–N st) cm^−1^; ^1^H NMR (400 MHz, CDCl_3_) *δ* 1.16–1.22 (m, 1H, 4′-H_a_), 1.42–1.56 (complex signal, 3H, 3′-H_a_, 3′-H_b_, 4′-H_b_), 2.17 (m, 1H, 4a′-H), 3.41 (ddd, *J* = *J′* = 11.2 Hz, *J″* = 3.2 Hz, 1H, 2′-H_a_), 3.62 (dm, *J* = 11.2 Hz, 1H, 2′-H_b_), 3.86 (br s, 1H, NH), 4.72 (d, *J* = 2.4 Hz, 1H, 5′-H), 5.27 (d, *J* = 5.6 Hz, 1H,10b′-H), 6.56 (d, *J* = 8.4 Hz, 1H, 7′-H), 7.06 (ddd, *J* = 8.4 Hz, *J′* = 2.4 Hz, *J″* = 0.8 Hz, 1H, 8′-H), 7.40 (dd, *J* = 2.4 Hz, *J′* = 0.8 Hz, 1H, 10′-H), 7.53 [dm, *J* = 8.4 Hz, 2H, C3(5)-H], 7.68 [dm, *J* = 8.4 Hz, 2H, C2(6)-H]; ^13^C NMR (100.6 MHz, CDCl_3_) *δ* 18.0 (CH_2_, C4′), 25.1 (CH_2_, C3′), 38.5 (CH, C4a′), 59.2 (CH, C5′), 60.8 (CH_2_, C2′), 72.1 (CH, C10b′), 111.6 (C, C1), 116.0 (CH, C7′), 118.6 (C, CN), 121.7 (C, C10a′), 123.9 (C, C9′), 127.3 (CH, C10′), 127.5 [2CH, C3(5)], 128.2 (CH, C8′), 132.3 [2CH, C2(6)], 143.0 (C, C6a′), 146.3 (C, C4); HRMS (ESI), calcd for [C_19_H_17_^35^ClN_2_O + H^+^] 325.1102, found 325.1101; Elemental analysis, calcd for C_19_H_17_ClN_2_O C 70.26%, H 5.28%, N 8.62%, found C 70.34%, H 5.37%, N 8.80%. HPLC purity >99%.

From diastereomeric mixture **37** (2.70 g, 8.31 mmol) and DDQ (3.77 g, 16.6 mmol), a solid residue (2.55 g) was obtained and purified through column chromatography (35–70 μm silica gel, hexane/EtOAc mixtures, gradient elution). On elution with hexane/EtOAc 80:20, compound **38** (1.09 g, 41% yield) was isolated as a white solid; *R*_*f*_ 0.57 (hexane/EtOAc 1:1).

A solution of **38** (200 mg, 0.62 mmol) in CH_2_Cl_2_ (15 mL) was filtered through a 0.2 μm PTFE filter and treated with a methanolic solution of HCl (0.53 N, 3.53 mL, 1.87 mmol). The resulting solution was evaporated at reduced pressure and the solid was washed with pentane (3 × 10 mL) to give, after drying under standard conditions, **38**·HCl (200 mg) as a beige solid: mp 222–224 °C; IR (ATR) *ν* 3650–2500 (max at 3628, 3342, 3085, 3061, 2921, 2560, ^+^NH and CH st), 2231 (CN st), 1634, 1602, 1576, 1505 (Ar–C–C and Ar–C–N st) cm^−1^; ^1^H NMR (400 MHz, CD_3_OD) *δ* 2.20 (tt, *J* = 6.0 Hz, *J* = 5.2 Hz, 2H, 3′-H_2_), 2.84 (t, *J* = 6.0 Hz, 2H, 4′-H_2_), 4.82 (t, *J* = 5.2 Hz, 2H, 2′-H_2_), 4.87 (s, ^+^NH), 7.97 (br d, *J* = 8.0 Hz, 2H), 8.08 (br d, *J* = 8.0 Hz, 2H) [C2(6)-H, C3(5)-H], overimposed in part 8.07 (dm, *J* = 8.8 Hz, 1H, 8′-H), 8.15 (d, *J* = 8.8 Hz, 1H, 7′-H), 8.42 (d, *J* = 2.0 Hz, 1H, 10′-H); ^13^C NMR (100.6 MHz, CD_3_OD) *δ* 21.4 (CH_2_, C3′), 23.3 (CH_2_, C4′), 71.5 (CH_2_, C2′), 115.8 (C, C4a′), 116.6 (C, C1), 118.7 (C, CN), 122.0 (C, C10a′), 123.09 (CH), 123.13 (CH) (C7′, C10′), 131.5 (2CH), 134.1 (2CH) [C2(6), C3(5)], 136.0 (C, C9′), 136.2 (CH, C8′), 136.5 (C, C4), 138.0 (C, C6a′), 155.9 (C, C5′), 166.5 (C, C10b′); HRMS (ESI), calcd for [C_19_H_13_^35^ClN_2_O + H^+^] 321.0789, found 321.0786; Elemental analysis, calcd for C_19_H_13_ClN_2_O·HCl·0.4H_2_O C 62.62%, H 4.09%, N 7.69%, found C 62.79%, H 4.26%, N 7.48%. HPLC purity >99%.

#### 4.1.20 4-{9-Chloro-3,4-dihydro-2H-pyrano[3,2-c]quinolin-5-yl}benzylamine **39**

It was prepared as described for **3**. From nitrile **38** (1.00 g, 3.12 mmol) and LiAlH_4_ (59 mg, 1.55 mmol), a residue (1.29 g) was obtained and purified by column chromatography (35–70 μm silica gel, EtOAc/MeOH/50% aq. NH_4_OH mixtures, gradient elution). On elution with EtOAc/MeOH/50% aq. NH_4_OH 95:5:0.2 to 80:20:0.2, the amine **39** (595 mg, 59% yield) was isolated as a yellow solid; *R*_*f*_ 0.14 (EtOAc/MeOH/50% aq. NH_4_OH 9:1:0.05).

A solution of **39** (595 mg, 1.84 mmol) in CH_2_Cl_2_ (25 mL) was filtered through a 0.2 μm PTFE filter and treated with a methanolic solution of HCl (0.53 N, 31 mL, 16.4 mmol). The resulting solution was evaporated at reduced pressure and the solid was recrystallized from MeOH/hexane 1:4 (5 mL) and washed with pentane (3 × 20 mL) to give, after drying under standard conditions, **39**·2HCl (720 mg) as a beige solid: mp 153–155 °C; IR (ATR) *ν* 3500–2500 (max at 3374, 2858, 2706, 2614, ^+^NH and CH st), 1634, 1615, 1604, 1580, 1513 (Ar–C–C and Ar–C–N st) cm^−1^; ^1^H NMR (400 MHz, CD_3_OD) *δ* 2.18 (tt, *J* = 6.4 Hz, *J′* = 5.2 Hz, 2H, 3′-H_2_), 2.85 (t, *J* = 6.4 Hz, 2H, 4′-H_2_), 4.32 (s, 2H, 1-C*H*_2_-NH_2_), 4.80 (t, *J* = 5.2 Hz, 2H, 2′-H_2_), 4.86 (s, ^+^NH, ^+^NH_3_), 7.82 (d, *J* = 8.4 Hz, 2H), 7.86 (d, *J* = 8.4 Hz, 2H) [C2(6)-H, C3(5)-H], 8.04 (dd, *J* = 8.8 Hz, *J′* = 2.4 Hz, 1H, 8′-H), 8.15 (d, *J* = 8.8 Hz, 1H, 7′-H), 8.40 (d, *J* = 2.4 Hz, 1H, 10′-H); ^13^C NMR (100.6 MHz, CD_3_OD) *δ* 21.5 (CH_2_, C3′), 23.6 (CH_2_, C4′), 43.8 (CH_2_, 1-CH_2_-NH_2_), 71.3 (CH_2_, C2′), 115.7 (C, C4a′), 121.9 (C, C10a′), 123.0 (CH), 123.3 (CH) (C7′, C10′), 130.8 (2CH), 131.1 (2CH) [C2(6), C3(5)], 133.2 (C, C4), 135.7 (C, C9′), 135.9 (CH, C8′), 138.17 (C), 138.23 (C) (C1, C6a′), 157.4 (C, C5′), 166.0 (C, C10b′); HRMS (ESI), calcd for [C_19_H_17_^35^ClN_2_O + H^+^] 325.1102, found 325.1096; Elemental analysis, calcd for C_19_H_17_ClN_2_O·2HCl·1.1H_2_O C 54.65%, H 5.12%, N 6.71%, found C 54.96%, H 5.66%, N 6.44%. HPLC purity: 96%.

#### 4.1.21 9-Chloro-5-(2-furyl)-3,4-dihydro-2H-pyrano[3,2-c]quinoline **42**

It was prepared as described for **8**. From 4-chloroaniline, **10** (1.52 g, 11.9 mmol), furan-2-carboxaldehyde, **40** (0.98 mL, 1.14 g, 11.8 mmol), Sc(OTf)_3_ (1.18 g, 2.40 mmol), and 3,4-dihydro-2*H*-pyran, **36** (1.08 mL, 994 mg, 11.8 mmol), and heating the reaction mixture at 50 °C, a brown oily residue (4.67 g) was obtained and used in the next step without further purification.

From this crude (4.67 g) and DDQ (5.39 g, 23.7 mmol), a brown solid residue (4.22 g) was obtained and purified through column chromatography (35–70 μm silica gel, hexane/EtOAc mixtures, gradient elution). On elution with hexane/EtOAc 80:20, compound **42** (916 mg, 27% yield) was isolated as a yellowish solid. On elution with hexane/EtOAc 50:50, 3-[6-chloro-2-(2-furyl)quinolin-3-yl]-1-propanol, **44** (1.01 g, 30% yield) was isolated as a yellowish solid; *R*_*f*__(**42**)_ 0.51; *R*_*f*__(**44**)_ 0.35 (hexane/EtOAc 1:1).

Compound **42** (916 mg, 3.21 mmol) was recrystallized from hexane/EtOAc 57:43 (7 mL), taken in CH_2_Cl_2_ (15 mL), filtered through a 0.2 μm PTFE filter and treated with a methanolic solution of HCl (0.53 N, 11 mL, 5.83 mmol). The resulting solution was evaporated at reduced pressure and the solid was washed with pentane (3 × 15 mL) to give, after drying under standard conditions, **42**·HCl (585 mg) as a yellowish solid: mp 243–245 °C; IR (ATR) *ν* 3500–2400 (max at 3085, 2435, ^+^NH and CH st), 1629, 1602, 1580, 1514 (Ar–C–C and Ar–C–N st) cm^−1^; ^1^H NMR (400 MHz, CD_3_OD) *δ* 2.30 (tt, *J* = 6.4 Hz, *J′* = 5.6 Hz, 2H, 3-H_2_), 3.17 (t, *J* = 6.4 Hz, 2H, 4-H_2_), 4.79 (t, *J* = 5.6 Hz, 2H, 2-H_2_), 4.85 (s, ^+^NH), 6.96 (dd, *J* = 3.6 Hz, *J′* = 1.6 Hz, 1H, 4′-H), 7.72 (d, *J* = 3.6 Hz, 1H, 3′-H), 7.99 (dd, *J* = 8.8 Hz, *J′* = 2.4 Hz, 1H, 8-H), 8.18 (d, *J* = 1.6 Hz, 1H, 5′-H), 8.26 (d, *J* = 8.8 Hz, 1H, 7-H), 8.29 (d, *J* = 2.4 Hz, 1H, 10-H); ^13^C NMR (100.6 MHz, CD_3_OD) *δ* 21.3 (CH_2_, C3), 23.7 (CH_2_, C4), 70.8 (CH_2_, C2), 113.4 (C, C4a), 115.3 (CH, C4′), 121.2 (C, C10a), 122.7 (CH, C3′), 122.8 (CH, C7), 122.9 (CH, C10), 135.3 (C, C9), 135.9 (CH, C8), 137.7 (C, C6a), 144.8 (C, C5), 145.2 (C, C2′), 149.8 (CH, C5′), 165.6 (C, C10b); HRMS (ESI), calcd for [C_16_H_12_^35^ClNO_2_ + H^+^] 286.0629, found 286.0629; Elemental analysis, calcd for C_16_H_12_ClNO_2_·HCl C 59.65%, H 4.07%, N 4.35%, found C 59.72%, H 4.00%, N 4.46%. HPLC purity: 97%.

A solution of **44** (100 mg, 0.35 mmol) in CH_2_Cl_2_ (7 mL) was filtered through a 0.2 μm PTFE filter and treated with a methanolic solution of HCl (0.53 N, 2.1 mL, 1.11 mmol). The resulting solution was evaporated at reduced pressure and the solid was recrystallized from MeOH/hexane 1:2 (4.5 mL) and washed with pentane (3 × 5 mL) to give, after drying under standard conditions, **44**·HCl (110 mg) as a brown solid: mp 202–203 °C; IR (ATR) *ν* 3500–2400 (max at 3384, 3121, 3072, 3053, 2927, 2487, ^+^NH, OH, and CH st), 1696, 1633, 1589, 1556, 1524 (Ar–C–C and Ar–C–N st) cm^−1^; ^1^H NMR (400 MHz, CD_3_OD) *δ* 2.03 (tt, *J* = 8.0 Hz, *J′* = 6.0 Hz, 2H, 2-H_2_), 3.30 (t, *J* = 8.0 Hz, 2H, 3-H_2_), 3.76 (t, *J* = 6.0 Hz, 2H, 1-H_2_), 4.88 (s, OH and ^+^NH), 6.99 (dd, *J* = 3.6 Hz, *J* = 1.6 Hz, 1H, 4″-H), 7.93 (d, *J* = 3.6 Hz, 1H, 3″-H), 8.05 (dd, *J* = 9.2 Hz, *J′* = 2.4 Hz, 1H, 7′-H), 8.21 (d, *J* = 1.6 Hz, 1H, 5″-H), 8.28 (d, *J* = 2.4 Hz, 1H, 5′-H), 8.38 (d, *J* = 9.2 Hz, 1H, 8′-H), 8.93 (s, 1H, 4′-H); ^13^C NMR (100.6 MHz, CD_3_OD) *δ* 30.6 (CH_2_, C3), 32.8 (CH_2_, C2), 61.8 (CH_2_, C1), 115.6 (CH, C4″), 123.2 (CH, C3″), 123.3 (CH, C8′),128.3 (CH, C5′), 129.4 (C, C4a′), 136.0 (CH, C7′), 136.2 (C, C6′), 136.4 (C, C8a′), 137.1 (C, C3′), 144.7 (C, C2′), 145.6 (C, C2″), 146.5 (CH, C4′), 150.4 (CH, C5″); HRMS (ESI), calcd for [C_16_H_14_^35^ClNO_2_ + H^+^] 288.0786, found 288.0785; Elemental analysis, calcd for C_16_H_14_ClNO_2_·HCl C 59.28%, H 4.66%, N 4.32%, found C 58.92%, H 4.68%, N 4.10%.

#### 4.1.22 9-Chloro-3,4-dihydro-5-(2-thienyl)-2H-pyrano[3,2-c]quinoline **43**

It was prepared as described for **8**. From 4-chloroaniline, **10** (1.52 g, 11.9 mmol), thiophene-2-carboxaldehyde, **41** (1.11 mL, 1.33 g, 11.9 mmol), Sc(OTf)_3_ (1.17 g, 2.38 mmol), and 3,4-dihydro-2*H*-pyran, **36** (1.08 mL, 994 mg, 11.8 mmol), and heating the reaction mixture at 80 °C, a brown oily residue (3.64 g) was obtained and used in the next step without further purification.

From this crude (3.64 g) and DDQ (5.40 g, 23.8 mmol), a residue (4.07 g) was obtained and purified through column chromatography (35–70 μm silica gel, hexane/EtOAc mixtures, gradient elution). On elution with hexane/EtOAc 90:10, compound **43** (1.43 g, 40% yield) was isolated. On elution with hexane/EtOAc 60:40, 3-[6-chloro-2-(2-thienyl)quinolin-3-yl]-1-propanol, **45** (854 mg, 24% yield) was isolated; *R*_*f*__(**43**)_ 0.70 (hexane/EtOAc 7:3); *R*_*f*__(**45**)_ 0.34 (hexane/EtOAc 1:1).

A solution of **43** (500 mg, 1.66 mmol) in CH_2_Cl_2_ (10 mL) was filtered through a 0.2 μm PTFE filter and treated with a methanolic solution of HCl (0.53 N, 9.4 mL, 4.98 mmol). The resulting solution was evaporated at reduced pressure and the solid was recrystallized from MeOH/EtOAc 1:2 (3 mL) and washed with pentane (3 × 8 mL) to give, after drying under standard conditions, **43**·HCl (469 mg) as a yellowish solid: mp 224–225 °C; IR (ATR) *ν* 3500–2500 (max at 3245, 3056, 2935, 2603, ^+^NH and CH st), 1629, 1602, 1569, 1530 (Ar–C–C and Ar–C–N st) cm^−1^; ^1^H NMR (400 MHz, CD_3_OD) *δ* 2.22 (tt, *J* = 6.0 Hz, *J′* = 5.4 Hz, 2H, 3-H_2_), 3.08 (t, *J* = 6.0 Hz, 2H, 4-H_2_), 4.79 (t, *J* = 5.4 Hz, 2H, 2-H_2_), 4.88 (s, ^+^NH), 7.43 (dd, *J* = 5.2 Hz, *J′* = 4.0 Hz, 1H, 4′-H), 7.88 (dd, *J* = 4.0 Hz, *J′* = 1.2 Hz, 1H, 3′-H), 8.02 (dd, *J* = 8.8 Hz, *J′* = 2.4 Hz, 1H, 8-H), 8.08 (dd, *J* = 5.2 Hz, *J′* = 1.2 Hz, 1H, 5′-H), 8.16 (d, *J* = 8.8 Hz, 1H, 7-H), 8.35 (d, *J* = 2.4 Hz, 1H, 10-H); ^13^C NMR (100.6 MHz, CD_3_OD) *δ* 21.5 (CH_2_, C3), 24.1 (CH_2_, C4), 71.1 (CH_2_, C2), 115.7 (C, C4a), 121.7 (C, C10a), 123.0 (2CH, C7, C10), 129.5 (CH, C4′), 131.9 (C, C2′), 134.2 (CH, C5′), 134.9 (CH, C3′), 135.5 (C, C9), 136.0 (CH, C8), 138.1 (C, C6a), 151.6 (C, C5), 165.9 (C, C10b); HRMS (ESI), calcd for [C_16_H_12_^35^ClNOS + H^+^] 302.0401, found 302.0399; Elemental analysis, calcd for C_16_H_12_ClNOS·HCl C 56.81%, H 3.87%, N 4.14%, S 9.48%, found C 56.99%, H 3.85%, N 3.98%, S 9.27%. HPLC purity: 97%.

A solution of **45** (411 mg, 1.35 mmol) in CH_2_Cl_2_ (4.5 mL) was filtered through a 0.2 μm PTFE filter and treated with a methanolic solution of HCl (0.53 N, 8.1 mL, 4.29 mmol). The resulting solution was evaporated at reduced pressure and the solid was washed with pentane (3 × 5 mL) to give, after drying under standard conditions, **45**·HCl (110 mg) as a yellowish solid: mp 198–199 °C; IR (ATR) *ν* 3500–2400 (max at 3337, 3076, 3029, 2918, 2886, 2476, ^+^NH, OH and CH st), 1633, 1583, 1538, 1514 (Ar–C–C and Ar–C–N st) cm^−1^; ^1^H NMR (400 MHz, CD_3_OD) *δ* 1.92 (tt, *J* = 8.0 Hz, *J′* = 6.0 Hz, 2H, 2-H_2_), 3.21 (t, *J* = 8.0 Hz, 2H, 3-H_2_), 3.63 (t, *J* = 6.0 Hz, 2H, 1-H_2_), 4.92 (s, OH and ^+^NH), 7.43 (dd, *J* = 5.2 Hz, *J′* = 4.0 Hz, 1H, 4″-H), 7.90 (dd, *J* = 4.0 Hz, *J′* = 1.2 Hz, 1H, 3″-H), 8.07 (dd, *J* = 9.2 Hz, *J′* = 2.0 Hz, 1H, 7′-H), superimposed in part 8.08 (dd, *J* = 5.2 Hz, *J′* = 1.2 Hz, 1H, 5″-H), 8.28 (d, *J* = 9.2 Hz, 1H, 8′-H), 8.34 (d, *J* = 2.0 Hz, 1H, 5′-H), 9.01 (s, 1H, 4′-H); ^13^C NMR (100.6 MHz, CD_3_OD) *δ* 30.4 (CH_2_, C3), 33.7 (CH_2_, C2), 61.8 (CH_2_, C1), 123.6 (CH, C8′), 128.3 (CH, C5′), 129.6 (CH, C4″), 130.2 (C, C4a′), 132.4 (C, C2″), 134.3 (CH, C5″), 135.1 (CH, C3″), 135.9 (CH, C7′), 136.7 (C, C6′), 137.6 (C, C8a′), 138.6 (C, C3′), 146.2 (CH, C4′), 151.9 (C, C2′); HRMS (ESI), calcd for [C_16_H_14_^35^ClNOS + H^+^] 304.0557, found 304.0555; Elemental analysis, calcd for C_16_H_14_ClNOS·HCl.1/5H_2_O C 55.89%, H 4.51%, N 4.07%, found C 55.86%, H 4.73%, N 3.55%.

#### 4.1.23 N-(tert-butyldimethylsilyloxy)-N-[3-(6-chloro-2-(2-thienyl)quinolin-3-yl)propyl]-4-methylbenzenesulfonamide **46**

To a solution of alcohol **45** (400 mg, 1.32 mmol) in anhydrous toluene (4 mL) and THF (1.2 mL), *N*-(*tert*-butyldimethylsilyloxy)-4-methylbenzenesulfonamide (397 mg, 1.32 mmol) and PPh_3_ (691 mg, 2.63 mmol) were added. The mixture was cooled to 0 °C and treated dropwise with DEAD (40% solution in toluene, 0.86 mL, 1.98 mmol). The reaction mixture was stirred at room temperature for 5 min, concentrated at reduced pressure and purified through column chromatography (35–70 μm silica gel, hexane/EtOAc mixtures, gradient elution). On elution with hexane/EtOAc 90:10, slightly impure **46** (913 mg) was isolated; *R*_*f*_ 0.60 (hexane/EtOAc 1:1); mp 91–92 °C; IR (ATR) *ν* 1594, 1548, 1522 (Ar–C–C and Ar–C–N st), 1359, 1168 (SO_2_) cm^−1^; ^1^H NMR (400 MHz, CDCl_3_) *δ* 0.09 [s, 6H, Si(CH_3_)_2_], 0.72 [s, 9H, Si[C(CH_3_)_3_]], 1.80 (tt, *J* = *J′* = 7.4 Hz, 2H, 2-H_2_), 2.25 (s, 3H, tosyl CH_3_), 2.81 (t, *J* = 7.2 Hz, 2H), 2.90 (t, *J* = 7.6 Hz, 2H) (1-H_2_ and 3-H_2_), 6.97 (dd, *J* = 4.8 Hz, *J′* = 4.0 Hz, 1H, 4″-H), 7.09–7.18 (complex signal), 7.20–7.24 (complex signal) [4H, 3″-H, 5″-H, and tosyl-C3(5)-H], 7.42 (dd, *J* = 9.2 Hz, *J′* = 2.0 Hz, 1H, 7′-H), 7.51 [br d, *J* = 8.4 Hz, 2H, tosyl-C2(6)-H], 7.57 (d, *J* = 2.0 Hz, 1H, 5′-H), 7.74 (s, 1H, 4′-H), 7.85 (d, *J* = 9.2 Hz, 1H, 8′-H); ^13^C NMR (100.6 MHz, CDCl_3_) *δ* – 4.1 [2CH_3_, Si(CH_3_)_3_], 18.4 [C, Si*C*(CH_3_)_3_], 21.8 (CH_3_, tosyl CH_3_), 26.1 [3CH_3_, SiC(*C*H_3_)_3_], 27.3 (CH_2_, C2), 30.8 (CH_2_, C3), 55.4 (CH_2_, C1), significant aromatic signals: 125.7 (CH, C8′), 129.5 [2CH, tosyl-*C*3(5)], 130.0 [2CH, tosyl-*C*2(6)], 132.4 (C), 132.8 (C) (C6′, C8a′), 136.0 (CH, C4′), 144.8 (C, C3′), 152.9 (C, C2′); HRMS (ESI), calcd for [C_29_H_35_^35^ClN_2_O_3_S_2_Si + H^+^] 587.1620, found 587.1596.

#### 4.1.24 3-[6-Chloro-2-(2-thienyl)quinolin-3-yl]propanenitrile **47**

To a solution of **46** (450 mg of a total amount of 913 mg of slightly impure **46**, obtained from 1.32 mmol of alcohol **45**) in anhydrous CH_3_CN (8 mL), CsF (233 mg, 1.53 mmol) was added. The reaction mixture was stirred at 60 °C for 2.5 h, diluted with saturated aq. NH_4_Cl (20 mL), and extracted with EtOAc (2 × 20 mL). The combined organic extracts were washed with brine (50 mL), dried over anhydrous Na_2_SO_4_ and evaporated at reduced pressure to give a crude (252 mg), which was purified through column chromatography (35–70 μm silica gel, hexane/EtOAc mixtures, gradient elution). On elution with hexane/EtOAc 70:30, nitrile **47** (164 mg, 84% overall yield from alcohol **45**) was isolated as a yellowish solid; *R*_*f*_ 0.57 (hexane/EtOAc 1:1).

A solution of **47** (32 mg, 0.11 mmol) in CH_2_Cl_2_ (4.5 mL) was filtered through a 0.2 μm PTFE filter and treated with a methanolic solution of HCl (0.53 N, 1.7 mL, 0.90 mmol). The resulting solution was evaporated at reduced pressure and the solid was washed with pentane (3 × 5 mL) to give, after drying under standard conditions, **47**·HCl (34 mg) as a yellowish solid: mp 65–67 °C; IR (ATR) *ν* 3600–2400 (max at 3371, 3111, 3070, 2925, 2853, 2599, ^+^NH and CH st), 2243 (CN st), 1636, 1591, 1540 (ar–C–C and ar–C–N st) cm^−1^; ^1^H NMR (400 MHz, CD_3_OD) *δ* 2.89 (t, *J* = 5.6 Hz, 2H, 2-H_2_), 3.46 (m, 2H, 3-H_2_), 4.92 (s, ^+^NH), 7.42 (m, 1H, 4″-H), 7.86 (br d, *J* = 2.8 Hz, 1H, 3″-H), 8.02–8.08 (complex signal, 2H, 7′-H, 5″-H), 8.25 (d, *J* = 8.8 Hz, 1H, 8′-H), 8.33 (br s, 1H, 5′-H), 9.01 (s, 1H, 4′-H); ^13^C NMR (100.6 MHz, CD_3_OD) *δ* 17.8 (CH_2_, C2), 29.5 (CH_2_, C3), 119.8 (C, C1), 125.7 (CH, C8′), 128.2 (CH, C5′), 129.6 (CH, C4″), 129.9 (C, C4a′), 133.3 (CH, C5″), 133.8 (CH, C3″), 134.3 (C, C3′), 134.8 (C, C2″), 135.3 (CH, C7′), 136.2 (C, C6′), 140.2 (C, C8a′), 144.2 (CH, C4′), 152.6 (C, C2′); HRMS (ESI), calcd for [C_16_H_11_^35^ClN_2_S + H^+^] 299.0404, found 299.0402; Elemental analysis, calcd for C_16_H_11_ClN_2_S·HCl.1.3H_2_O C 53.58%, H 4.10%, found C 53.80%, H 3.79%; HPLC purity: 96%.

#### 4.1.25 3-[6-Chloro-2-(2-thienyl)quinolin-3-yl]propyl methanesulfonate **48**

A solution of alcohol **45** (494 mg, 1.63 mmol) and freshly distilled Et_3_N (271 μL, 197 mg, 1.94 mmol) was cooled to 0 °C and treated dropwise with MsCl (163 μL, 241 mg, 2.11 mmol) over 10 min. The reaction mixture was stirred at 0 °C for 30 min, poured into a mixture of H_2_O (30 mL) and ice (6 mL), and extracted with CH_2_Cl_2_ (2 × 20 mL). The combined organic extracts were washed with saturated aq. NaHCO_3_ (20 mL), brine (20 mL), dried over anhydrous Na_2_SO_4_ and evaporated at reduced pressure to give crude mesylate **48** (633 mg), which was used in the next step without further purification; *R*_*f*_ 0.50 (hexane/EtOAc 1:1); mp 130–132 °C; IR (ATR) *ν* 1593, 1548, 1522 (ar–C–C and ar–C–N st), 1358, 1339, 1169 (SO_2_) cm^−1^; ^1^H NMR (400 MHz, CDCl_3_) *δ* 2.11 (tt, *J* = 7.6 Hz, *J′* = 6.0 Hz, 2H, 2-H_2_), 3.00 (s, 3H, CH_3_SO_3_), 3.21 (t, *J* = 7.6 Hz, 2H, 3-H_2_), 4.27 (t, *J* = 6.0 Hz, 2H, 1-H_2_), 7.20 (dd, *J* = 4.8 Hz, *J′* = 4.0 Hz, 1H, 4″-H), 7.55 (d, *J* = 4.8 Hz, 1H, 5″-H), 7.62 (d, *J* = 4.0 Hz, 1H, 3″-H), superimposed in part 7.64 (dd, *J* = 9.2 Hz, *J′* = 2.0 Hz, 1H, 7′-H), 7.77 (d, *J* = 2.0 Hz, 1H, 5′-H), 8.03 (s, 1H, 4′-H), 8.15 (d, *J* = 9.2 Hz, 1H, 8′-H); ^13^C NMR (100.6 MHz, CDCl_3_) *δ* 29.3 (CH_2_), 29.5 (CH_2_) (C2, C3), 37.4 (CH_3_, CH_3_SO_3_), 68.6 (CH_2_, C1), 125.6 (C + CH, C4a′, C8′), 127.7 (C, C2″), 127.9 (CH), 129.0 (CH), 129.1 (CH), 129.6 (CH), 131.2 (CH) (C5′, C7′, C3″, C4″, C5″), 132.4 (C), 133.0 (C) (C6′, C8a′), 137.2 (CH, C4′), 143.7 (C, C3′), 152.2 (C, C2′); HRMS (ESI), calcd for [C_17_H_16_^35^ClNO_3_S_2_ + H^+^] 382.0333, found 382.0335.

#### 4.1.26 N-{3-[6-Chloro-2-(2-thienyl)quinolin-3-yl]propyl}pyrrolidine **49**

To a solution of crude mesylate **48** (296 mg of a total amount of 633 mg of crude mesylate **48** obtained from 1.63 mmol of alcohol **45**) in anhydrous DMF (10 mL), pyrrolidine (0.13 mL, 111 mg, 1.56 mmol) and K_2_CO_3_ (322 mg, 2.33 mmol) were added. The reaction mixture was stirred at 85 °C overnight, cooled to room temperature, treated with saturated aq. NaHCO_3_ (20 mL), and extracted with EtOAc (3 × 30 mL). The combined organic extracts were washed with H_2_O (3 × 15 mL), dried over anhydrous Na_2_SO_4_ and evaporated at reduced pressure to give a crude (165 mg), which was purified through column chromatography (35–70 μm silica gel, EtOAc/MeOH/50% aq. NH_4_OH mixtures, gradient elution). On elution with EtOAc/MeOH/50% aq. NH_4_OH 80:20:0.2, amine **49** (84 mg, 31% overall yield from alcohol **45**) was isolated; *R*_*f*_ 0.10 (hexane/EtOAc 1:1).

A solution of **49** (84 mg, 0.24 mmol) in CH_2_Cl_2_ (4.5 mL) was filtered through a 0.2 μm PTFE filter and treated with a methanolic solution of HCl (0.53 N, 4.0 mL, 2.12 mmol). The resulting solution was evaporated at reduced pressure and the solid was washed with pentane (3 × 5 mL) to give, after drying under standard conditions, **49**·2HCl (88 mg) as a yellowish solid: mp 136–138 °C; IR (ATR) *ν* 3200–2600 (max at 3076, 2941, 2931, 2858, ^+^NH and CH st), 1594, 1548, 1525 (ar–C–C and ar–C–N st) cm^−1^; ^1^H NMR (400 MHz, CD_3_OD) *δ* 2.04 (br signal, 2H, 2-H_2_), 2.12–2.26 [complex signal, 4H, pyrrolidine 3(4)-H_2_], 3.10 (br signal, 2H), 3.24 (br signal, 4H), 3.66 (br signal, 2H) [1-H_2_, 3-H_2_, pyrrolidine 2(5)-H_2_], 7.45 (br s, 1H, 4″-H), 7.93 (br s, 1H, 3″-H), 8.04–8.12 (complex signal, 2H, 7′-H, 5″-H), 8.30 (d, *J* = 8.4 Hz, 1H, 8′-H), 8.39 (br s, 1H, 5′-H), 9.14 (s, 1H, 4′-H); ^13^C NMR (100.6 MHz, CD_3_OD) *δ* 24.1 [2CH_2_, pyrrolidine C3(4)], 27.1 (CH_2_, C2), 30.6 (CH_2_, C3), 55.19 (CH_2_, C1), 55.21 [2CH_2_, pyrrolidine C2(5)], 123.8 (CH, C8′), 128.5 (CH, C5′), 129.8 (CH, C4″), 130.2 (C, C4a′), 132.2 (C, C2″), 134.4 (CH, C5″), 135.4 (CH, C3″), 136.1 (CH, C7′), 136.66 (C), 136.73 (C) (C6′, C8a′), 137.9 (C, C3′), 146.3 (CH, C4′), 152.0 (C, C2′); HRMS (ESI), calcd for [C_20_H_21_^35^ClN_2_S + H^+^] 357.1187, found 357.1187; Elemental analysis, calcd for C_20_H_21_ClN_2_S·2HCl.3H_2_O C 49.64%, H 6.04%, N 5.79%, found C 49.98%, H 5.72%, N 5.29%; HPLC purity: 96%.

#### 4.1.27 N-{3-[6-Chloro-2-(2-thienyl)quinolin-3-yl]propyl}-N,N-diethylamine **50**

It was prepared as described for **49**. From crude mesylate **48** (293 mg of a total amount of 585 mg of crude mesylate **48** obtained from 0.82 mmol of alcohol **45**) and freshly distilled Et_2_NH (320 μL, 226 mg, 3.09 mmol), a crude product (157 mg) was obtained and purified by column chromatography (35–70 μm silica gel, EtOAc/MeOH/50% aq. NH_4_OH mixtures, gradient elution). On elution with EtOAc/MeOH/50% aq. NH_4_OH 80:20:0.2, the amine **50** (24 mg, 16% overall yield from alcohol **45**) was isolated; *R*_*f*_ 0.10 (hexane/EtOAc 1:1).

A solution of **50** (24 mg, 66.9 μmol) in CH_2_Cl_2_ (4.5 mL) was filtered through a 0.2 μm PTFE filter and treated with a methanolic solution of HCl (0.53 N, 1.2 mL, 0.64 mmol). The resulting solution was evaporated at reduced pressure and the solid was washed with pentane (3 × 5 mL) to give, after drying under standard conditions, **50**·2HCl (24 mg) as a yellowish solid: mp 98–100 °C; IR (ATR) *ν* 3500–2500 (max at 3422, 3117, 3071, 2933, 2863, 2648, ^+^NH and CH st), 1637, 1592, 1540 (ar–C–C and ar–C–N st) cm^−1^; ^1^H NMR (400 MHz, CD_3_OD) *δ* 1.32 [m, 6H, N(CH_2_C*H*_3_)_2_], 2.16 (br signal, 2H, 2-H_2_), 3.16–3.28 [complex signal, 8H, 1-H_2_, 3-H_2_, N(C*H*_2_CH_3_)_2_], 4.89 (s, ^+^NH), 7.43 (br s, 1H, 4″-H), 7.90 (br s, 1H, 3″-H), 8.04–8.09 (complex signal, 2H, 7′-H, 5″-H), 8.25 (d, *J* = 8.8 Hz, 1H, 8′-H), 8.36 (br s, 1H, 5′-H), 9.08 (s, 1H, 4′-H); ^13^C NMR (100.6 MHz, CD_3_OD) *δ* 9.24 [CH_3_, N(CH_2_*C*H_3_)_2_], 25.2 (CH_2_, C2), 30.7 (CH_2_, C3), 48.6 [CH_2_, N(*C*H_2_CH_3_)_2_], 52.2 (CH_2_, C1), 124.3 (CH, C8′), 128.4 (CH, C5′), 129.7 (CH, C4″), 130.2 (C, C4a′), 133.1 (C, C2″), 134.0 (CH, C5″), 134.9 (CH, C3″), 135.8 (CH, C7′), 136.5 (C), 136.6 (C) (C6′, C8a′), 138.5 (C, C3′), 145.6 (CH, C4′), 152.2 (C, C2′); HRMS (ESI), calcd for [C_20_H_23_^35^ClN_2_S + H^+^] 359.1343, found 359.1347; Elemental analysis, calcd for C_20_H_23_ClN_2_S·2HCl.2.5H_2_O C 50.37%, H 6.34%, N 5.87%, found C 50.55%, H 6.07%, N 5.38%; HPLC purity: > 99%.

### 4.2 Biological assays

#### 4.2.1 T. brucei culturing and evaluation of trypanocidal activity

Bloodstream form *T. brucei* (strain 221) was cultured at 37 °C in modified Iscove's medium [56]. Trypanocidal activity was assessed by growing parasites in the presence of various concentrations of the novel compounds and determining the levels which inhibited growth by 50% (IC_50_) and 90% (IC_90_). *T. brucei* in the logarithmic phase of growth were diluted back to 2.5 × 10^4^ mL^−1^ and aliquoted into 96-well plates. The compounds were then added at a range of concentrations and the plates incubated at 37 °C. Each drug concentration was tested in triplicate. Resazurin was added after 48 h and the plates incubated for a further 16 h and the plates then read in a Spectramax plate reader. Results were analysed using GraphPad Prism.

#### 4.2.2 T. cruzi and L. infantum culturing and evaluation of trypanocidal and leishmanicidal activity

Stock solutions of the novel compounds in DMSO were prepared at concentrations of 20 mg mL^−1^, with the final DMSO concentration being lower than 2% for all experiments. Trypanocidal and leishmanicidal activity was assessed by growing parasites in the presence of various concentrations of the novel compounds and determining the levels which inhibited growth by 50% (IC_50_). The IC_50_ was determined from a least-squares linear regression of growth rate versus log drug concentrations.

*T. cruzi* strain MHOM/ES/2203/BCN590 (Tcl) was used. Epimastigote forms were cultured in liver infusion tryptose broth (LIT) with 10% foetal bovine serum and 1% penicillin (100 U mL^−1^) – streptomycin (100 μg mL^−1^) solution (Sigma P-4333) at 28 °C.

For evaluation of *T. cruzi* activity serial dilutions of the novel compounds in LIT culture medium were aliquoted in 96-well microtiter plates (Costar 3596). Then 4 × 10^6^ mL^−1^ epimastigotes culture medium in the logarithmic growth phase were added to each well, incubating at 28 °C for 72 h. Benznidazole was used as the reference drug at concentrations from 2.50 mM to 2.42 μM. Parasite inhibition for each drug concentration was determined using an automated cell counter (TC20 BIO-RAD). All assays were performed in duplicate at least twice.

*L. infantum* strain MCAN/ES/92/BCN722, isolated from a dog with visceral leishmaniosis, was used. Promastigote forms were cultured in Schneider's insect medium (Sigma S-8995), pH 7, with 20% heat-inactivated foetal calf serum, 25 μg mL^−1^ gentamycin solution (Sigma G-1397), and 1% penicillin (100 U mL^−1^) – streptomycin (100 μg mL^−1^) solution (Sigma P-4333) at 26 °C. Serial dilutions of the novel compounds in Schneider culture medium were performed in 96-well microtiter plates (Costar 3596). Then 10^6^ mL^−1^ promastigotes in their logarithmic growth phase was added to each well (100 μL/well), incubating at 26 °C for 48 h. Potassium antimony (III) tartrate hydrate was used as the reference drug at concentrations from 815 to 0.80 μM. Growth was measured through the acid phosphatase activity [57]. All assays were performed in duplicate at least twice.

#### 4.2.3 Cytotoxic activity against rat skeletal myoblast L6 cells

Cytotoxicity against mammalian cells was assessed using microtitre plates following a described procedure [58]. Briefly, rat skeletal muscle L6 cells were seeded at 1 × 10^4^ mL^−1^ in 200 μL of growth medium containing different compound concentrations. The plates were incubated for 6 days at 37 °C and 20 μL resazurin was then added to each well. After a further 8 h incubation, the fluorescence was determined using a Spectramax plate reader.

#### 4.2.4 Acetylcholinesterase inhibitory activity

The inhibitory activity against *E. electricus* (Ee) AChE (Sigma–Aldrich) was evaluated spectrophotometrically by the method of Ellman et al. [59]. The reactions took place in a final volume of 300 μL of 0.1 M phosphate-buffered solution pH 8.0, containing EeAChE (0.03 u/mL) and 333 μM 5,5′-dithiobis(2-nitrobenzoic) acid (DTNB; Sigma–Aldrich) solution used to produce the yellow anion of 5-thio-2-nitrobenzoic acid. Inhibition curves were performed in duplicates using at least 10 increasing concentrations of inhibitors and preincubated for 20 min at 37 °C before adding the substrate. One duplicate sample without inhibitor was always present to yield 100% of AChE activity. Then substrate, acetylthiocholine iodide (450 μM; Sigma–Aldrich), was added and the reaction was developed for 5 min at 37 °C. The colour production was measured at 414 nm using a labsystems Multiskan spectrophotometer.

Data from concentration−inhibition experiments of the inhibitors were calculated by non-linear regression analysis, using the GraphPad Prism program package (GraphPad Software; San Diego, USA), which gave estimates of the IC_50_ (concentration of drug producing 50% of enzyme activity inhibition). Results are expressed as mean ± S.E.M. of at least 4 experiments performed in duplicate.

#### 4.2.5 Determination of brain permeability: PAMPA-BBB assay

The *in vitro* permeability (*P*_e_) of the novel compounds and fourteen commercial drugs through lipid extract of porcine brain membrane was determined by using a parallel artificial membrane permeation assay [50]. Commercial drugs and the target compounds were tested using a mixture of PBS:EtOH 70:30. Assay validation was made by comparing experimental and described permeability values of the commercial drugs, which showed a good correlation: *P*_e_ (exp) = 1.583 *P*_e_ (lit) – 1.079 (R^2^ = 0.9305). From this equation and the limits established by Di et al. for BBB permeation, three ranges of permeability were established: compounds of high BBB permeation (CNS+): *P*_e_ (10^−6^ cm s^−1^) > 5.25; compounds of low BBB permeation (CNS–): *P*_e_ (10^−6^ cm s^−1^) < 2.09; and compounds of uncertain BBB permeation (CNS±): 5.25 > *P*_e_ (10^−6^ cm s^−1^) > 2.09.

## Appendix A Supplementary material

Supplementary material related to this article can be found at http://dx.doi.org/. These data include calculated molecular properties of the novel compounds, CNS MPO desirability scores, additional results from the PAMPA-BBB assay, as well as copies of the ^1^H and ^13^C NMR spectra of the tested compounds.
