# Supplementary material for: Multicomponent reaction-based synthesis and biological evaluation of tricyclic heterofused quinolines with multi-trypanosomatid activity
Source: Eur J Med Chem. 2015 Nov 13;105:120–37. doi: 10.1016/j.ejmech.2015.10.007 (PMC4638191; doi:10.1016/j.ejmech.2015.10.007)

Supplementary Material

Multicomponent reaction-based synthesis and biological evaluation of tricyclic heterofused quinolines with multi-trypanosomatid activity

Ornella Di Pietro[a], Esther Vicente-García[b], Martin C. Taylor[c], Diana Berenguer[d], Elisabet Viayna[a], Anna Lanzoni[a], Irene Sola[a], Helena Sayago[b], Cristina Riera[d], Roser Fisa[d], M. Victòria Clos[e], Belén Pérez[e], John M. Kelly[c], Rodolfo Lavilla[b,f], Diego Muñoz-Torrero[a,*]

[a] *Laboratori de Química Farmacèutica (Unitat Associada al CSIC), Facultat de Farmàcia, and Institut de Biomedicina (IBUB), Universitat de Barcelona, Av. Joan XXIII, 27-31, E-08028, Barcelona, Spain*

[b] *Barcelona Science Park, Baldiri Reixac 10-12, E-08028, Barcelona, Spain*

[c] *Department of Pathogen Molecular Biology, London School of Hygiene and Tropical Medicine, Keppel Street, London WC1E 7HT, United Kingdom*

[d] *Laboratori de Parasitologia, Departament de Microbiologia i Parasitologia Sanitàries, Facultat de Farmàcia, Universitat de Barcelona, Av. Joan XXIII, 27-31, E-08028, Barcelona, Spain*

[e] *Departament de Farmacologia, de Terapèutica i de Toxicologia, Institut de Neurociències, Universitat Autònoma de Barcelona, E-08193, Bellaterra, Barcelona, Spain*

[f] *Laboratori de Química Orgànica, Facultat de Farmàcia, Universitat de Barcelona, Av. Joan XXIII, 27-31, E-08028, Barcelona, Spain*

* Corresponding author. Tel.: +34 934024533; fax: +34 934035941.

E-mail address: dmunoztorrero@ub.edu (D. Muñoz-Torrero).

S3 **Table S1.** Calculated molecular properties of the tested compounds.

S4 **Table S2.** Calculated CNS MPO desirability scores of the tested compounds.

S5 **Table S3.** Reported and experimental permeability values of the commercial drugs used for the PAMPA-BBB assay validation.

S5 **References**.

S6 **Copies of ^{1}H and ^{13}C NMR spectra of the tested compounds**.

Table S1

Calculated molecular properties of the novel heterofused quinolines and related compounds.[a]

Compd	miLogP	TPSA	MW	nON	nOHNH	nrotb	volume	nviolations
**1**	6.64	28.16	442.01	3	1	6	411.95	1
**2**	5.89	42.15	413.95	3	2	4	377.47	1
**3**	5.89	42.15	413.95	3	2	4	377.47	1
**26**	4.30	48.71	305.77	3	1	1	260.85	0
**27**	4.82	48.71	319.80	3	1	1	277.65	0
**28**	5.09	48.71	333.82	3	1	1	294.45	1
**29**	4.82	48.71	319.80	3	1	1	277.65	0
**30**	3.73	50.94	309.80	3	3	2	272.08	0
**31**	4.25	50.94	323.83	3	3	2	288.88	0
**32**	4.52	50.94	337.85	3	3	2	305.68	0
**33**	4.25	50.94	323.83	3	3	2	288.88	0
**35**	3.67	62.71	321.81	3	2	4	287.38	0
**37**	4.31	45.05	324.81	3	1	1	286.44	0
**38**	5.02	45.92	320.78	3	0	1	274.23	1
**39**	4.45	48.15	324.81	3	2	2	285.46	0
**42**	4.40	35.27	285.73	3	0	1	238.94	0
**43**	5.04	22.13	301.80	2	0	1	248.08	1
**44**	3.62	46.26	287.75	3	1	4	248.81	0
**45**	4.26	33.12	303.81	2	1	4	257.96	0
**47**	4.08	36.68	298.80	2	0	3	250.00	0
**49**	5.33	16.13	356.92	2	0	5	319.09	1
**50**	5.68	16.13	358.94	2	0	7	329.45	1

[a] Molecular properties (Log P, topological polar surface area (TPSA), molecular weight (MW), number of hydrogen bond acceptors (nON), number of hydrogen bond donors (nOHNH), number of rotatable bonds (nrotb), molecular volume, and number of violations of Lipinski's rules (nviolations)) calculated using Molinspiration (http://molinspiration.com).

Table S2

Calculated CNS MPO desirability scores of the novel heterofused quinolines and related compounds.[a]

Compd	pK_a[b]	cLogP[b]	cLogD[b]	CNS MPO
**1**	9.75	7.0	4.44	1.8
**2**	9.39	6.21	3.98	2.4
**3**	9.43	6.21	4.13	2.4
**26**	6.84	4.14	4.03	4.3
**27**	6.87	4.58	4.47	4.0
**28**	6.87	5.03	4.91	3.8
**29**	6.93	4.58	4.46	4.0
**30**	9.43	3.41	1.31	4.2
**31**	9.43	3.85	1.74	4.0
**32**	9.43	4.30	2.19	3.7
**33**	9.43	3.85	1.74	4.0
**35**	10.05	4.48	1.99	3.8
**37**	2.38	3.96	3.96	4.4
**38**	4.63	4.95	4.95	4.0
**39**	9.43	4.22	2.23	4.1
**42**	2.80	4.16	4.16	4.2
**43**	3.63	4.87	4.87	3.2
**44**	1.32	3.79	3.79	4.5
**45**	2.14	4.51	4.51	3.7
**47**	1.64	4.69	4.69	4.0
**49**	9.86	5.63	3.20	2.5
**50**	10.06	5.93	3.33	2.3

[a] CNS MPO scores calculated using the algorithm reported in ref. [1]. TPSA values, MW, and the number of hydrogen bond donors (nOHNH), used in the algorithm, are shown in Table S1.

[b] Marvin was used for predicting pK_a, cLogP, and cLogD values, Marvin 5.12.0, 2013, ChemAxon (http://www.chemaxon.com).

Table S3

Reported and experimental permeability values (P_e 10^{-6} cm s^{-1}) of the 14 commercial drugs used for the PAMPA-BBB assay validation.

Compound	Literature value[a]	Experimental value[b]
Cimetidine	0.0	0.70 ± 0.03
Lomefloxacin	1.1	0.70 ± 0.04
Norfloxacin	0.1	0.90 ± 0.02
Ofloxacin	0.8	0.98 ± 0.04
Hydrocortisone	1.9	1.40 ± 0.05
Piroxicam	2.5	1.80 ± 0.02
Clonidine	5.3	6.50 ± 0.05
Corticosterone	5.1	6.70 ± 0.10
Imipramine	13	12.3 ± 0.10
Promazine	8.8	13.8 ± 0.30
Progesterone	9.3	16.8 ± 0.30
Desipramine	12	17.8 ± 0.10
Testosterone	17	23.1 ± 0.20
Verapamil	16	25.8 ± 0.30

[a] Taken from ref. [2].

[b] Values are expressed as the mean ± SD of three independent experiments.

References

[1] T.T. Wager, X. Hou, P.R. Verhoest, A. Villalobos, Moving beyond the rules: The development of a central nervous system multiparameter optimization (CNS MPO) approach to enable alignment of druglike properties, ACS Chem. Neurosci. 1 (2010) 435–449.

[2] L. Di, E.H. Kerns, K. Fan, O.J. McConnell, G.T. Carter, High throughput artificial membrane permeability assay for blood-brain barrier, Eur. J. Med. Chem. 38 (2003) 223–232.

N-{1-Benzyl-5-(4-chlorophenyl)-1,2,3,4-tetrahydrobenzo[h][1,6]naphthyridin-9-yl}methanamine ***2***

^{1}H NMR (400 MHz , CD_3OD)

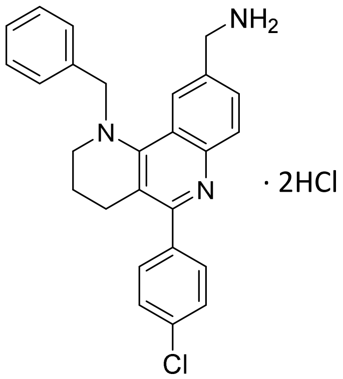

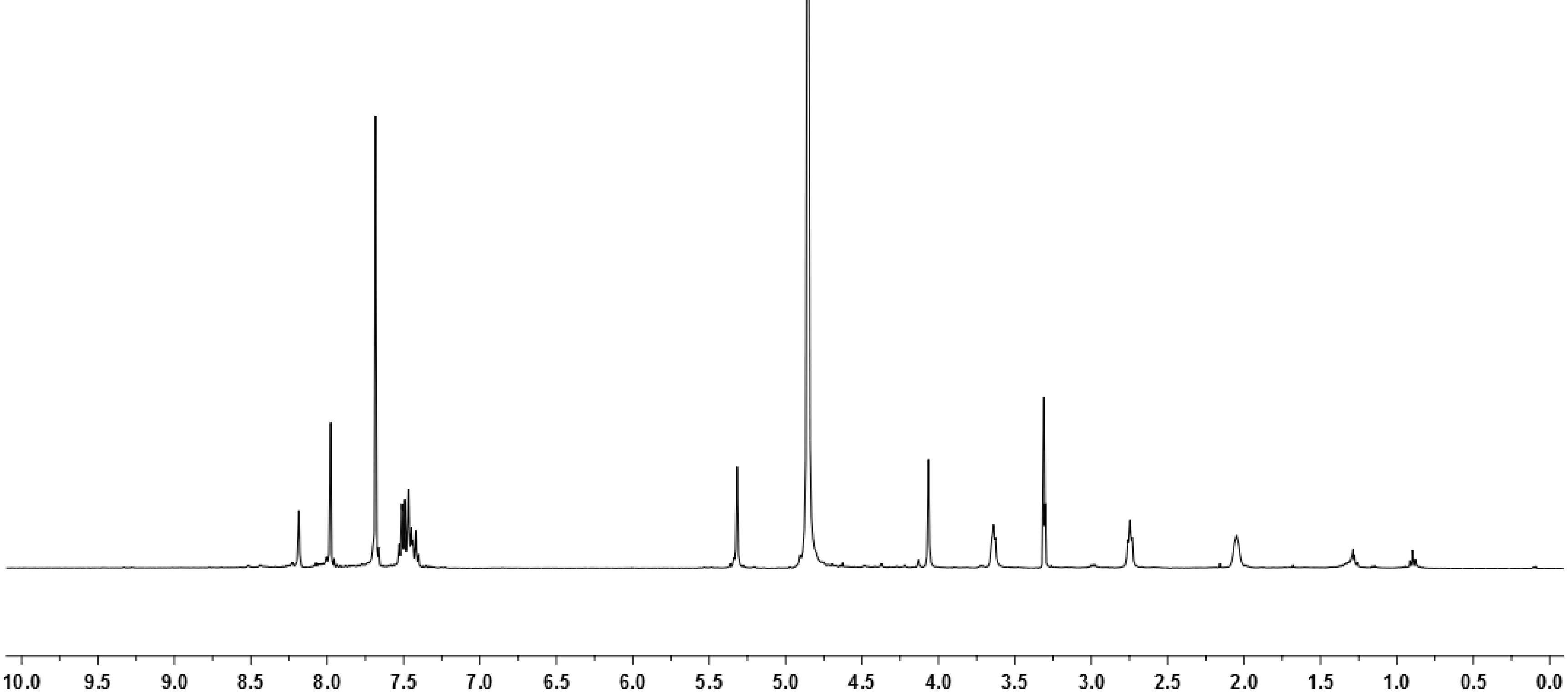

N-{1-Benzyl-5-(4-chlorophenyl)-1,2,3,4-tetrahydrobenzo[h][1,6]naphthyridin-9-yl}methanamine ***2***

^{13}C NMR (100.6 MHz, CD_3OD)

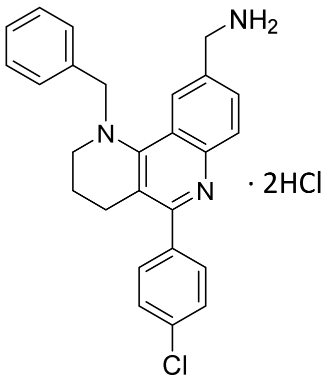

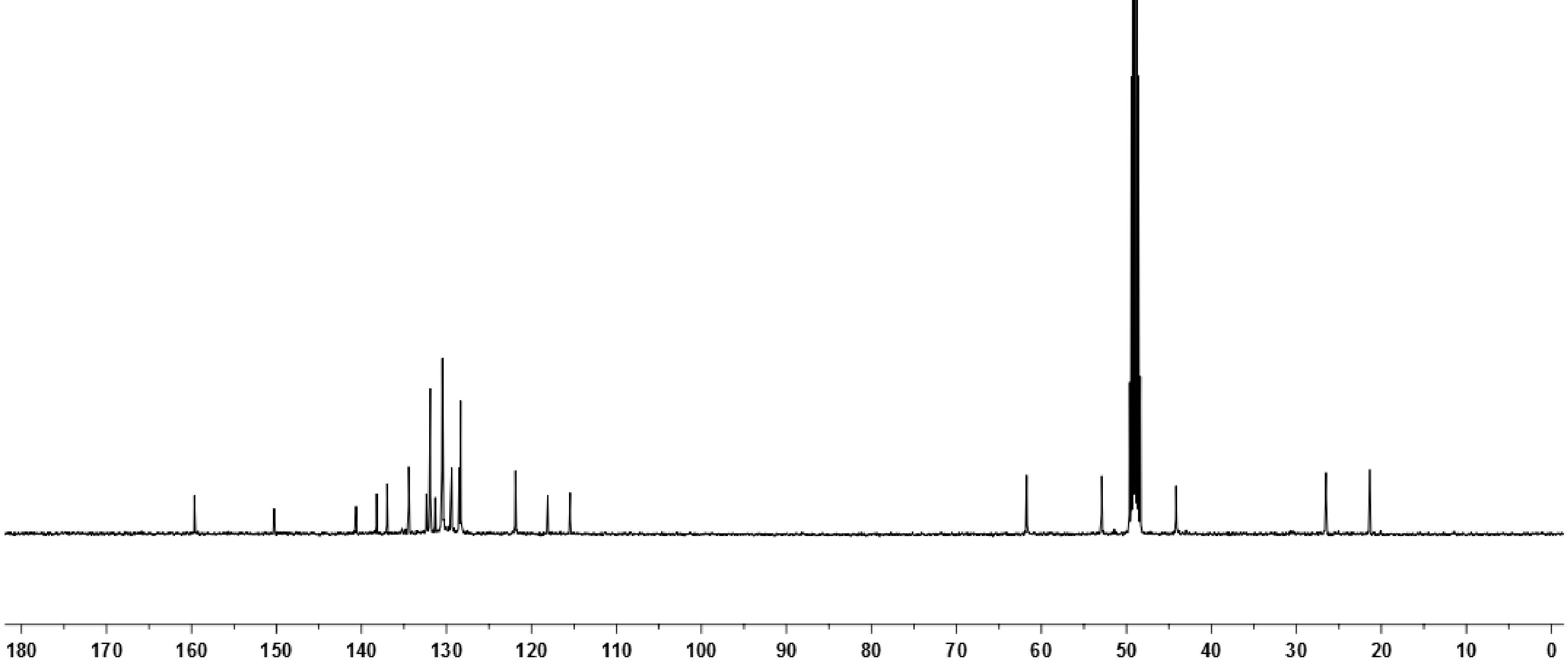

4-{1-Benzyl-9-chloro-1,2,3,4-tetrahydrobenzo[h][1,6]naphthyridin-5-yl}benzylamine ***3***

^{1}H NMR (400 MHz , CD_3OD)

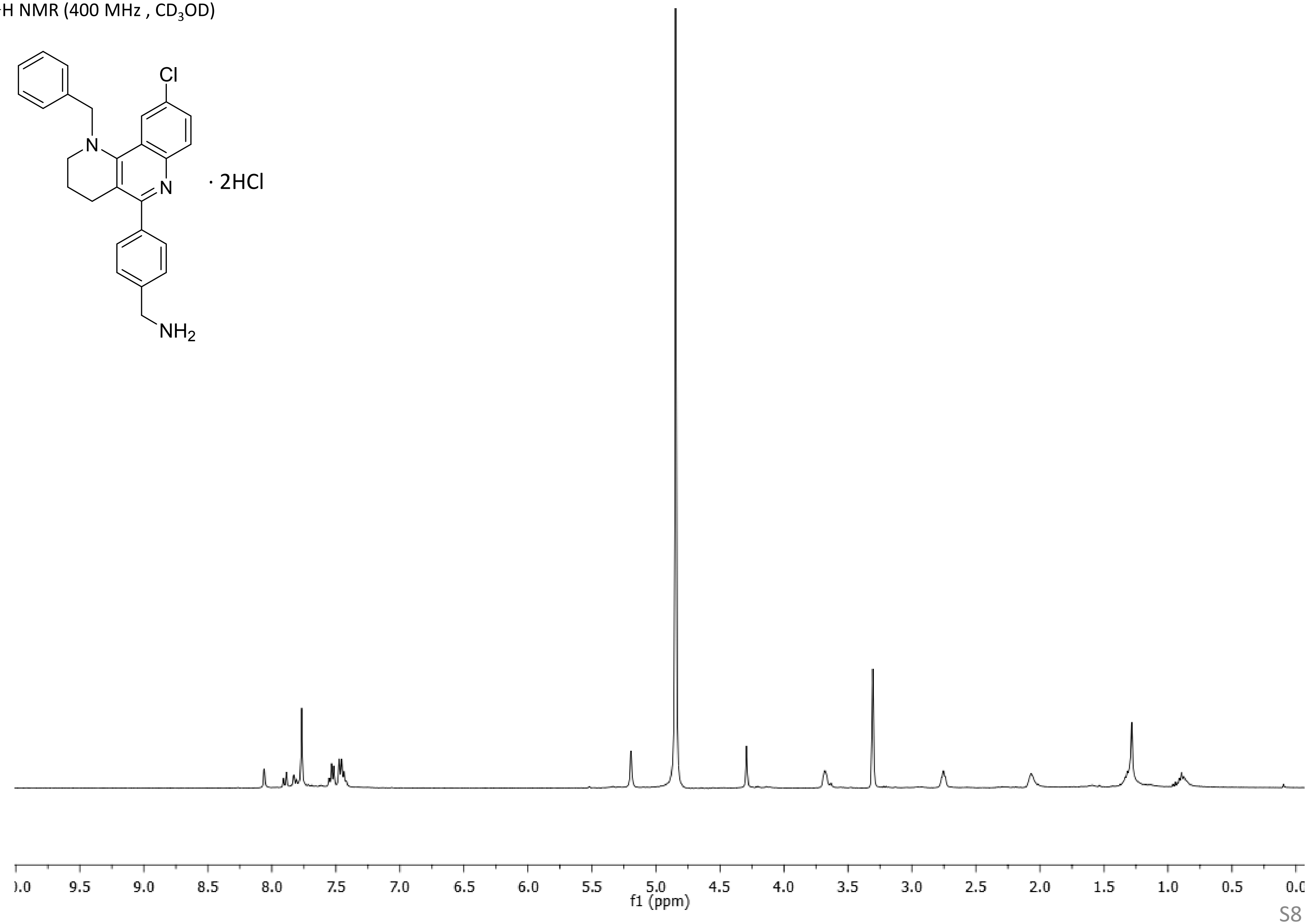

4-{1-Benzyl-9-chloro-1,2,3,4-tetrahydrobenzo[h][1,6]naphthyridin-5-yl}benzylamine ***3***

^{13}C NMR (100.6 MHz, CD_3OD)

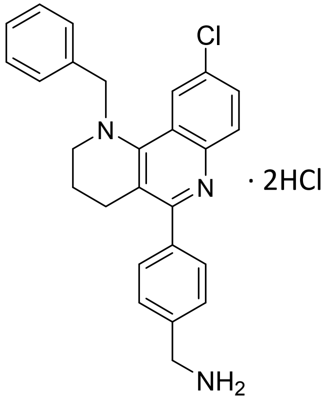

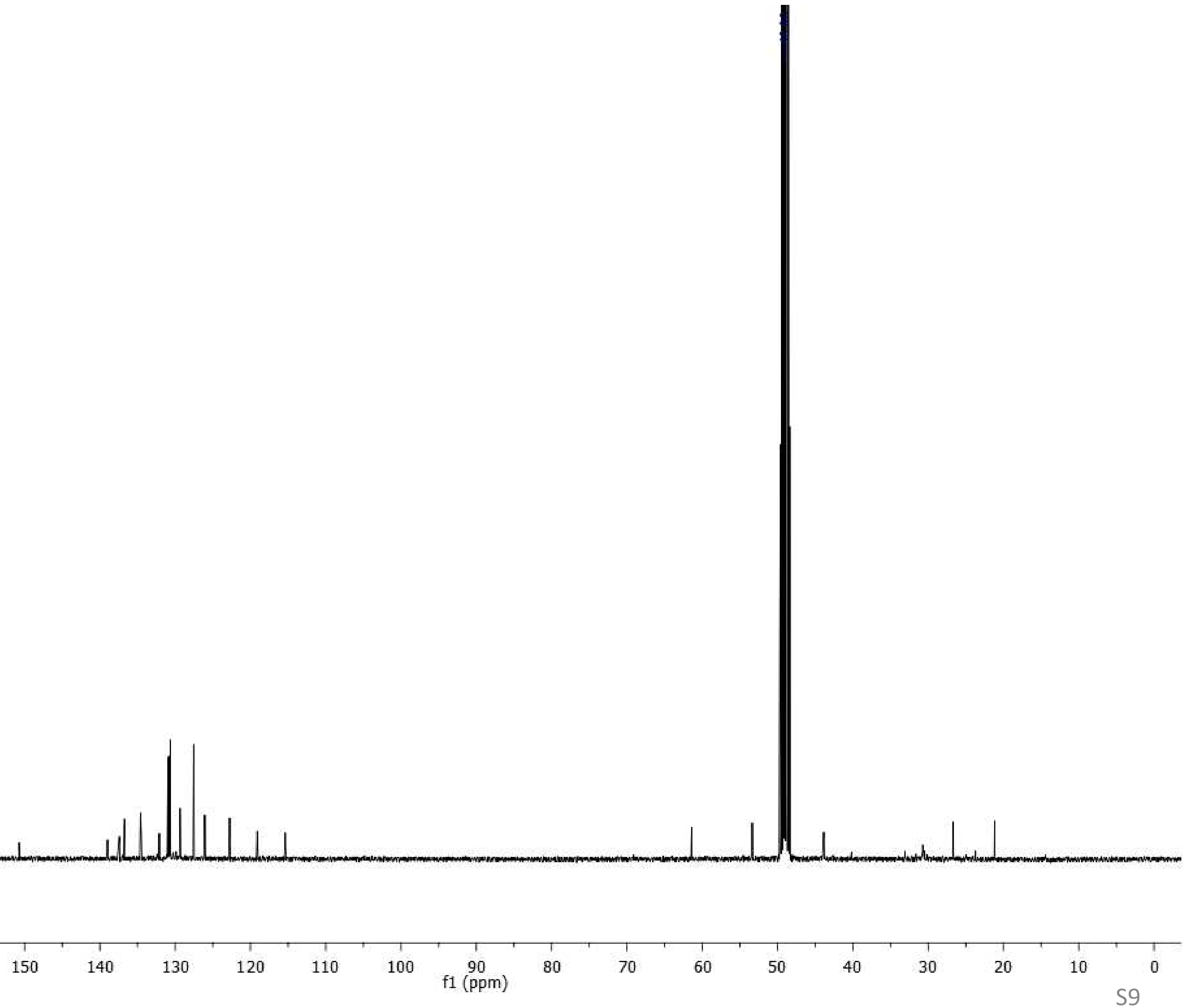

4-{8-Chloro-2,3-dihydro-1H-pyrrolo[3,2-c]quinolin-4-yl}benzonitrile ***26***

^{1}H NMR (400 MHz , CD_3OD)

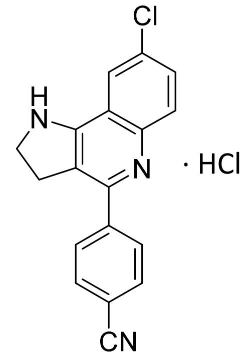

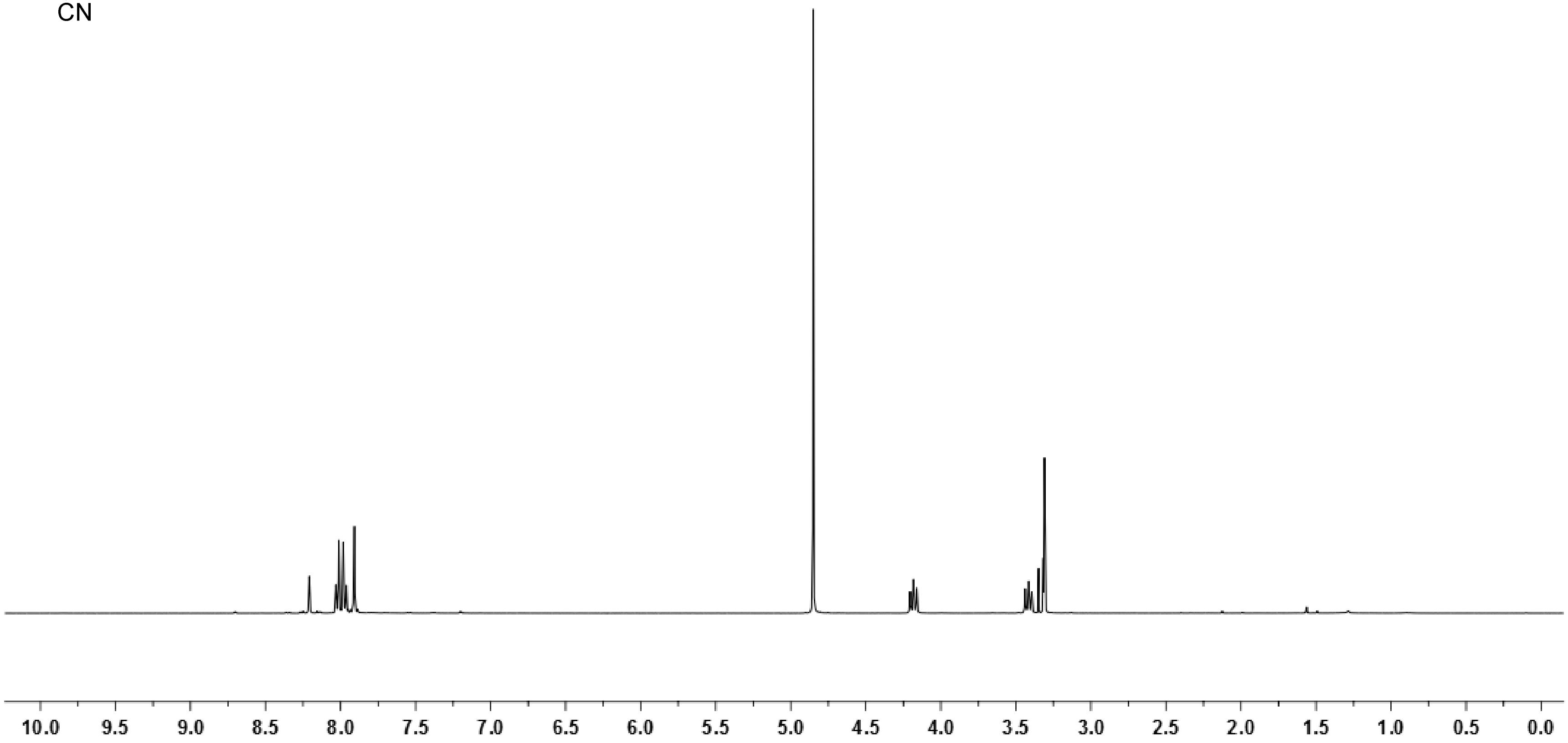

4-{8-Chloro-2,3-dihydro-1H-pyrrolo[3,2-c]quinolin-4-yl}benzonitrile ***26***

^{13}C NMR (100.6 MHz, CD_3OD)

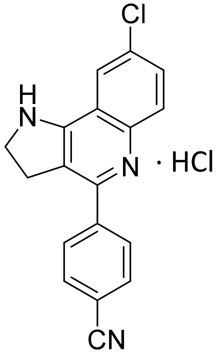

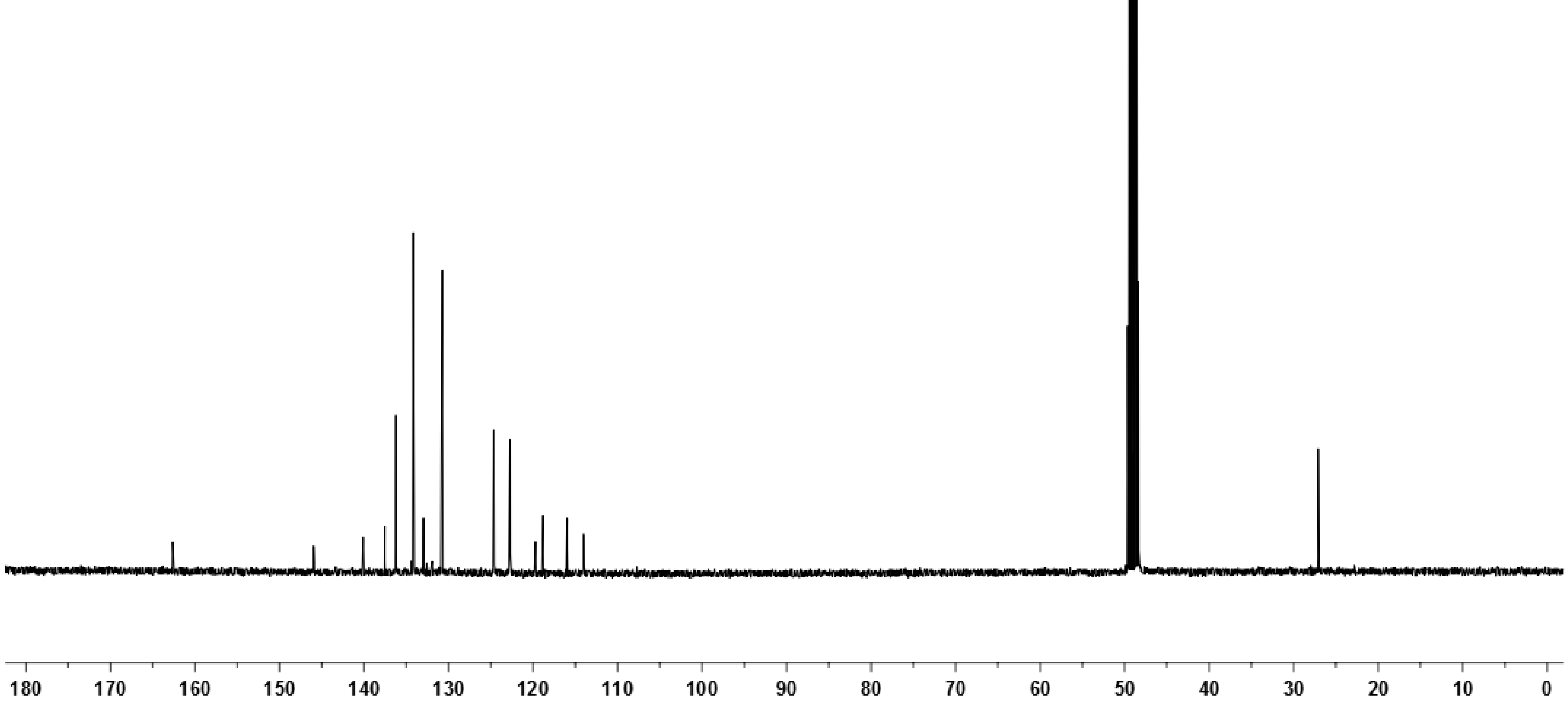

4-{9-Chloro-1,2,3,4-tetrahydrobenzo[h][1,6]naphthyridin-5-yl}benzonitrile ***27***

^{1}H NMR (400 MHz , CD_3OD)

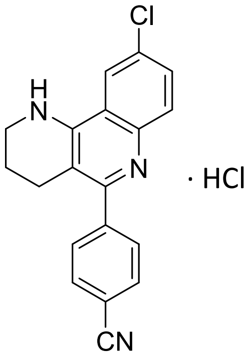

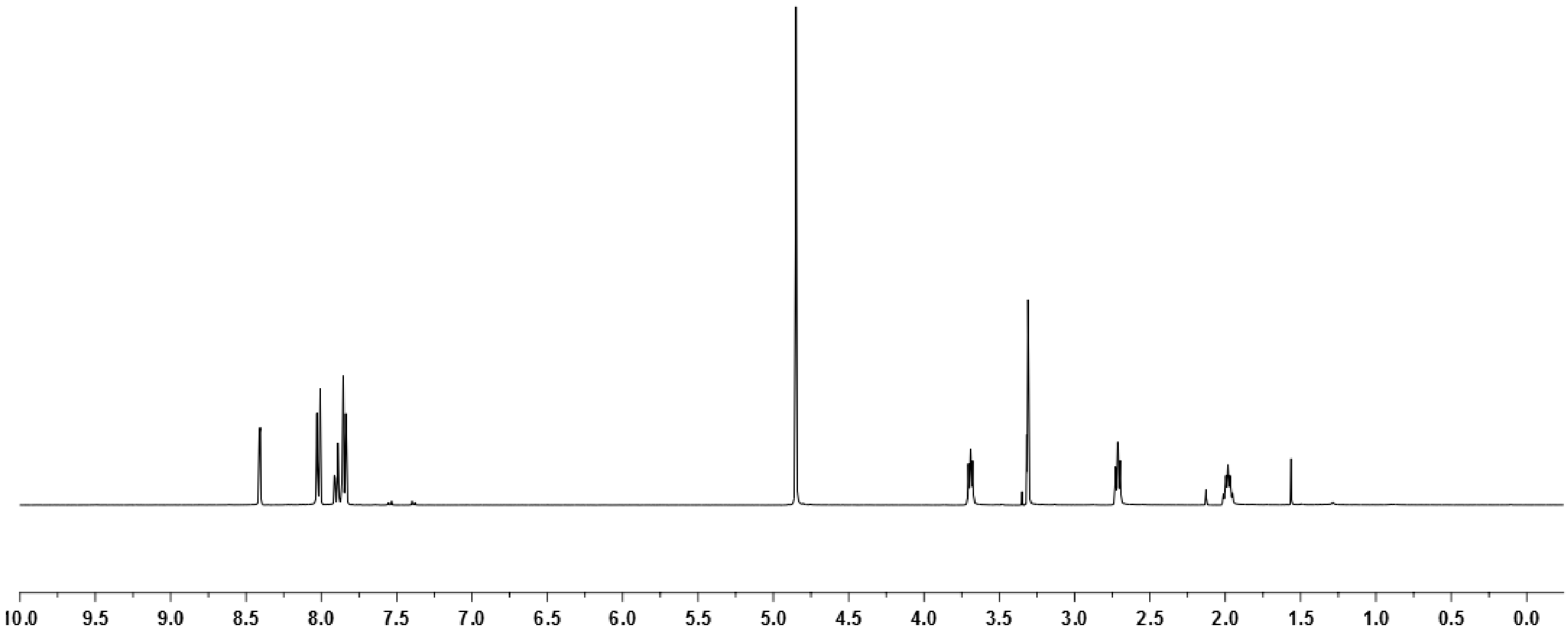

4-{9-Chloro-1,2,3,4-tetrahydrobenzo[h][1,6]naphthyridin-5-yl}benzonitrile ***27***

^{13}C NMR (100.6 MHz, CD_3OD)

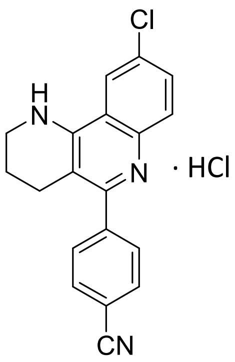

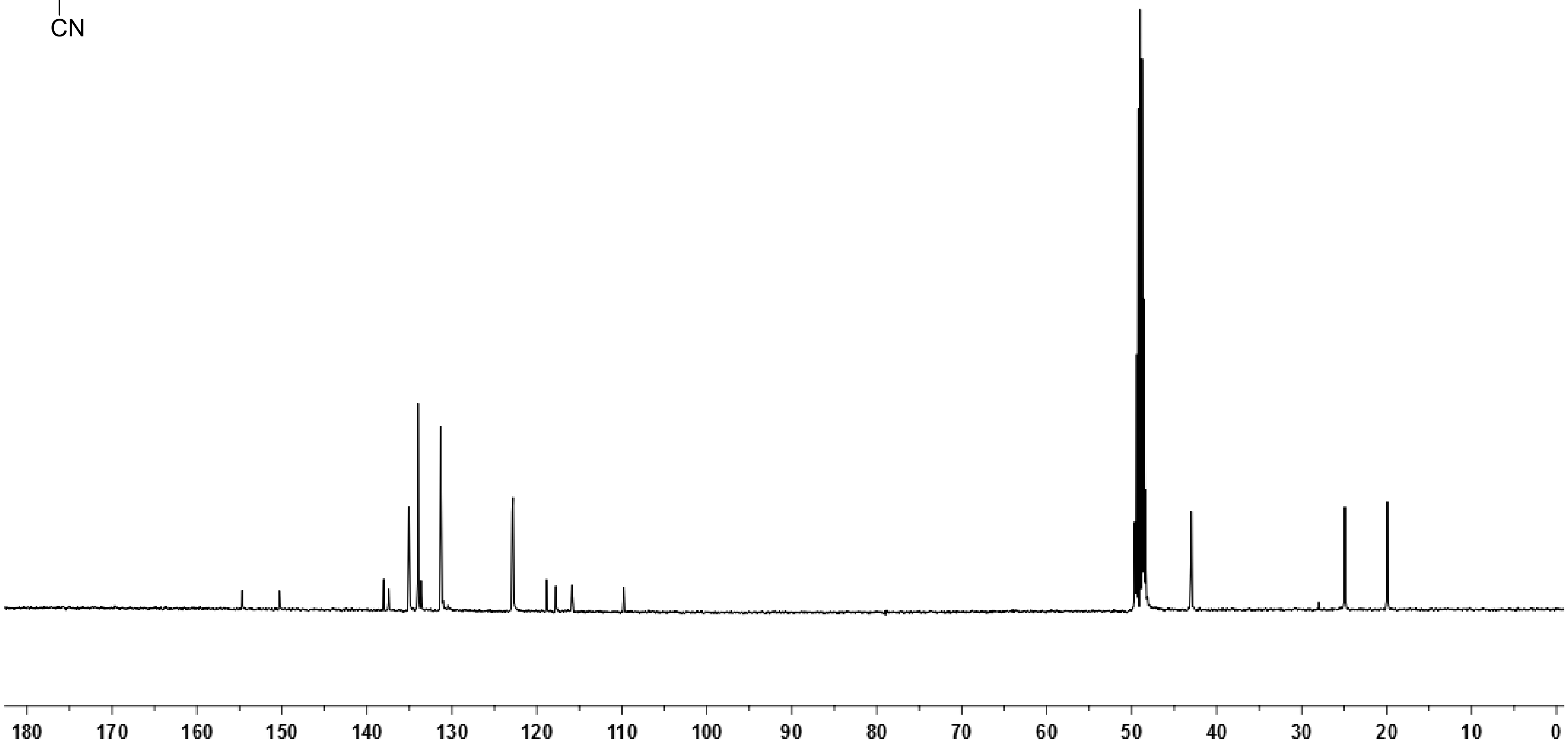

*4-{10-Chloro-2,3,4,5-tetrahydro-1H-azepino[3,2-c]quinolin-6-yl}benzonitrile **28***

^{1}H NMR (400 MHz , CD_3OD)

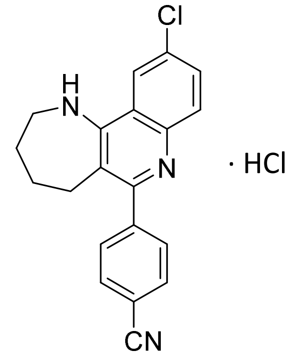

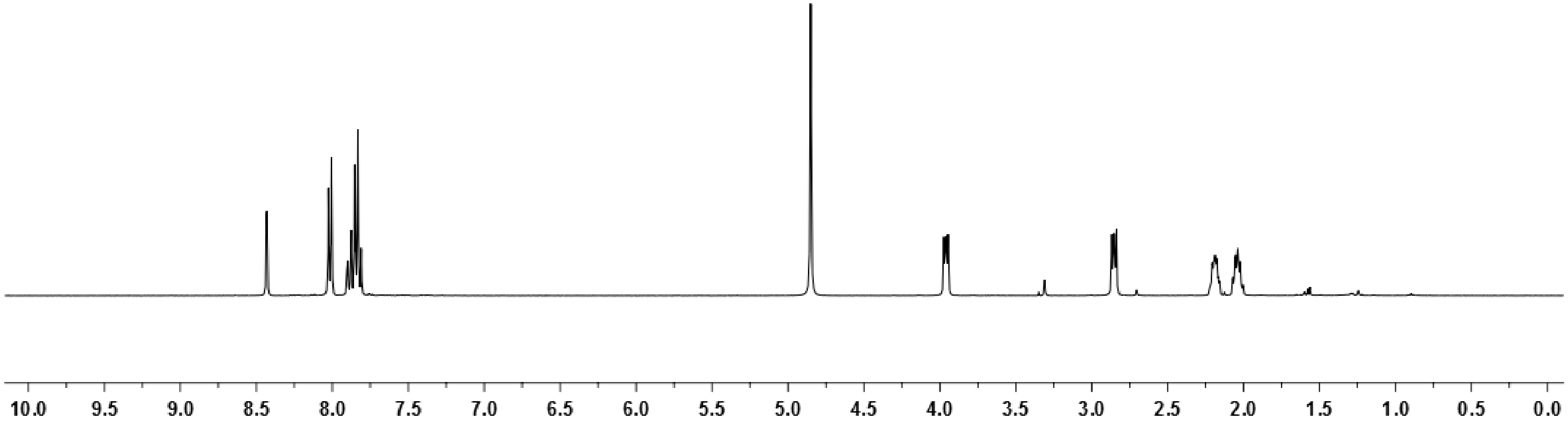

4-{10-Chloro-2,3,4,5-tetrahydro-1H-azepino[3,2-c]quinolin-6-yl}benzonitrile ***28***

^{13}C NMR (100.6 MHz, CD_3OD)

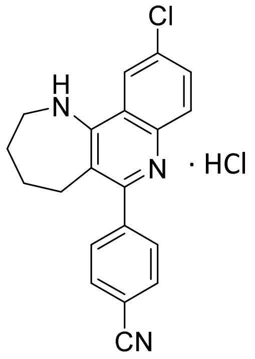

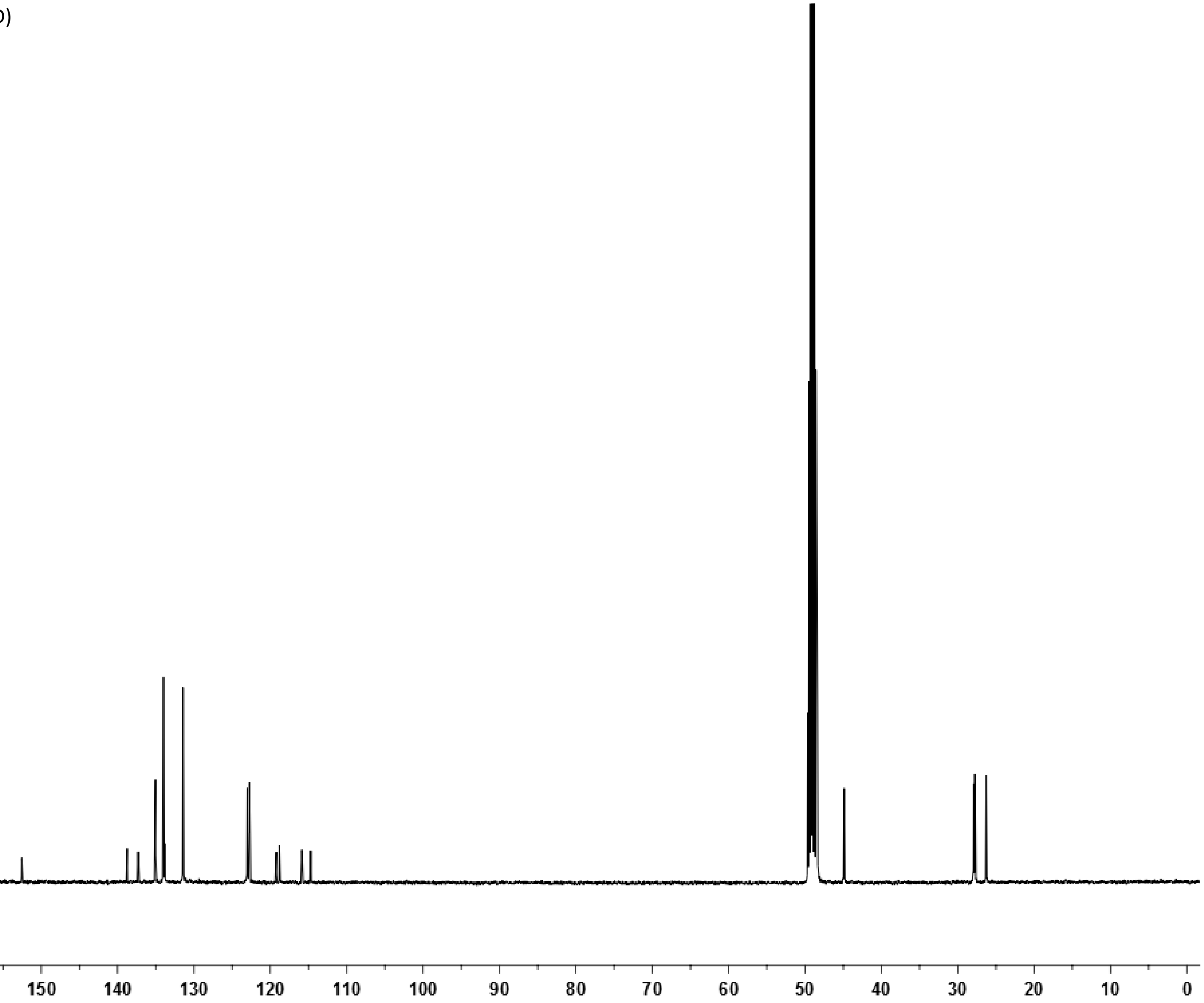

4-{8-Chloro-1,2,3,4-tetrahydrobenzo[h][1,6]naphthyridin-5-yl}benzonitrile ***29***

^{1}H NMR (400 MHz , CD_3OD)

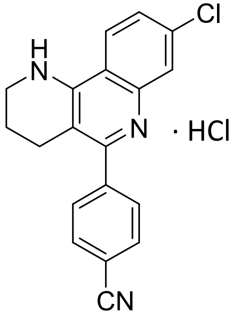

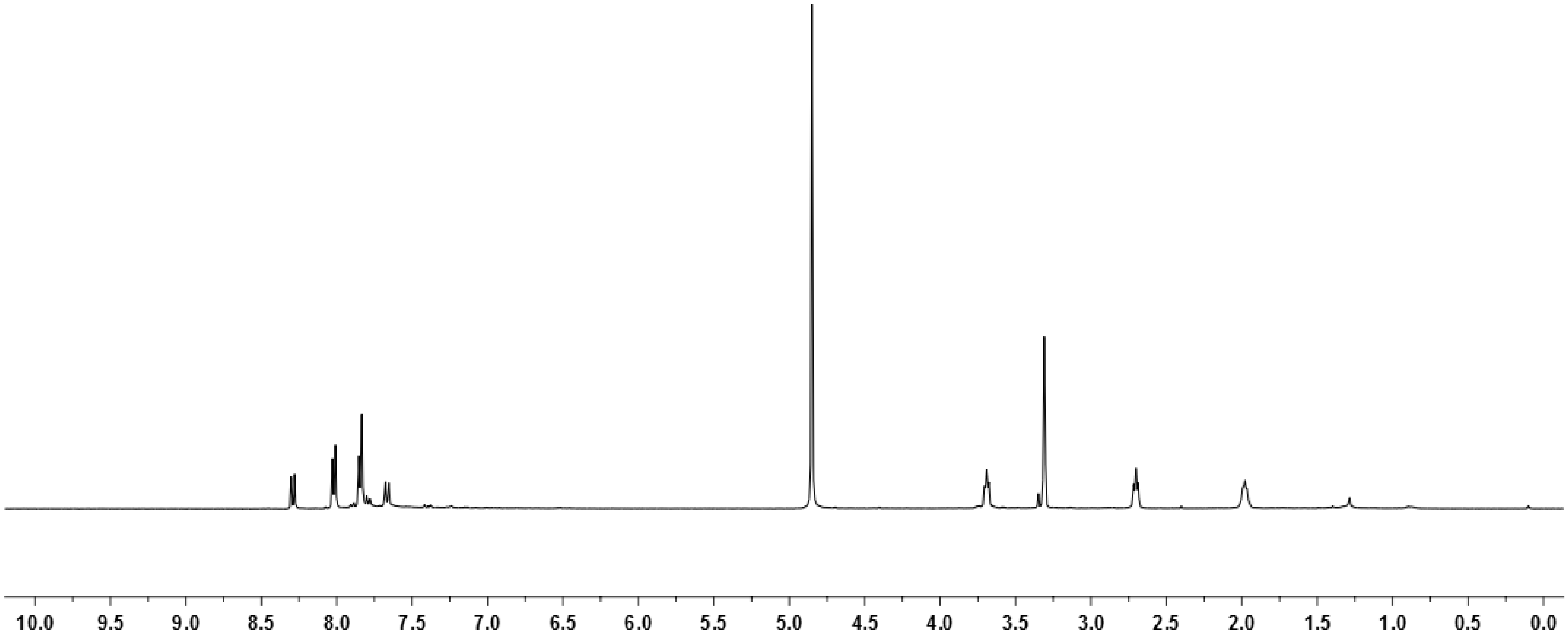

4-{8-Chloro-1,2,3,4-tetrahydrobenzo[h][1,6]naphthyridin-5-yl}benzonitrile ***29***

^{13}C NMR (100.6 MHz, CD_3OD)

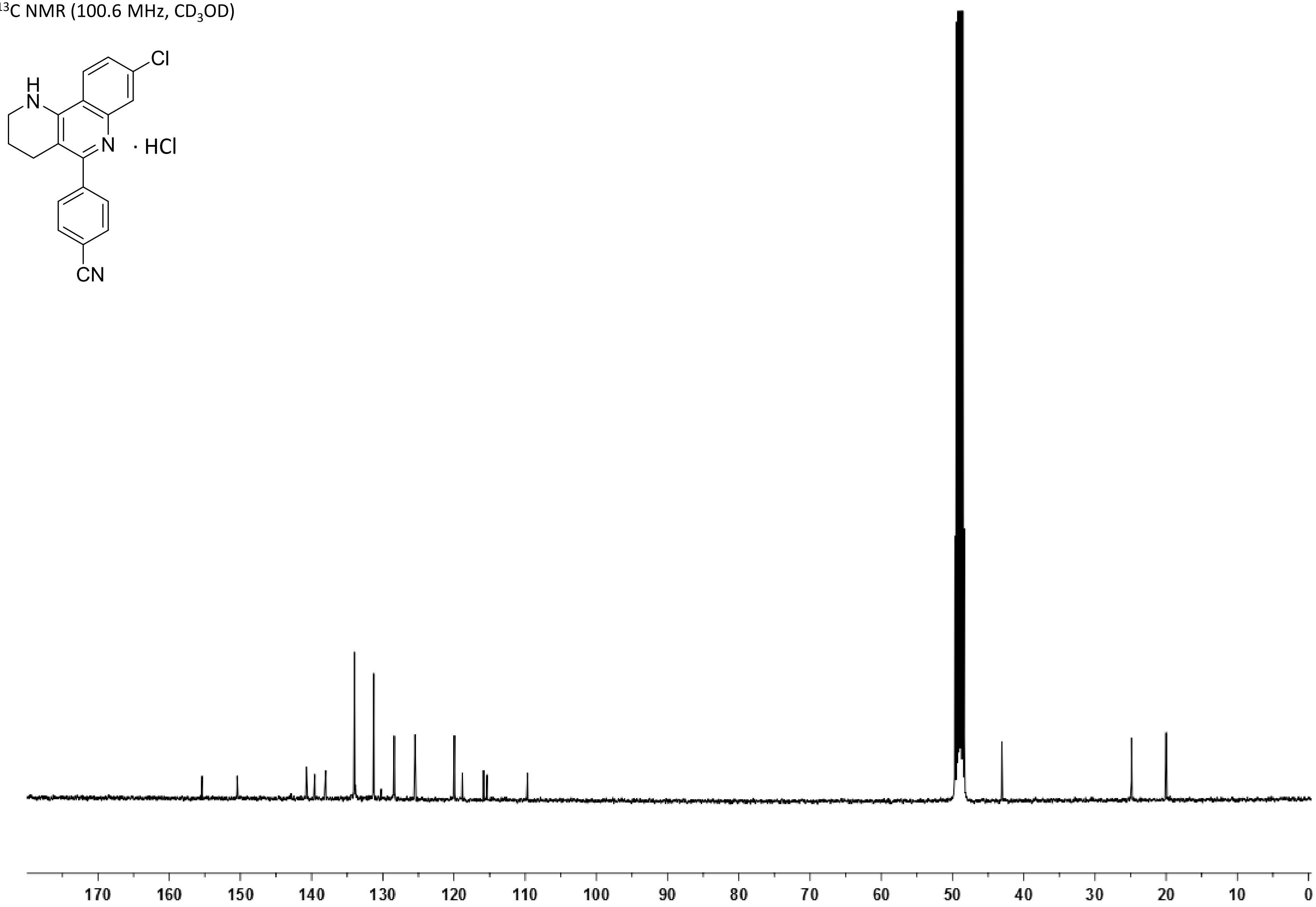

4-{8-Chloro-2,3-dihydro-1H-pyrrolo[3,2-c]quinolin-4-yl}benzylamine ***30***

^{1}H NMR (400 MHz , CD_3OD)

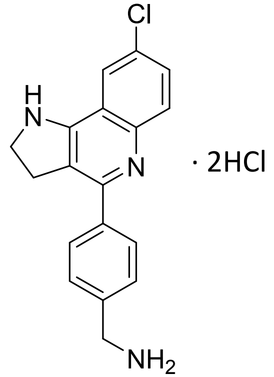

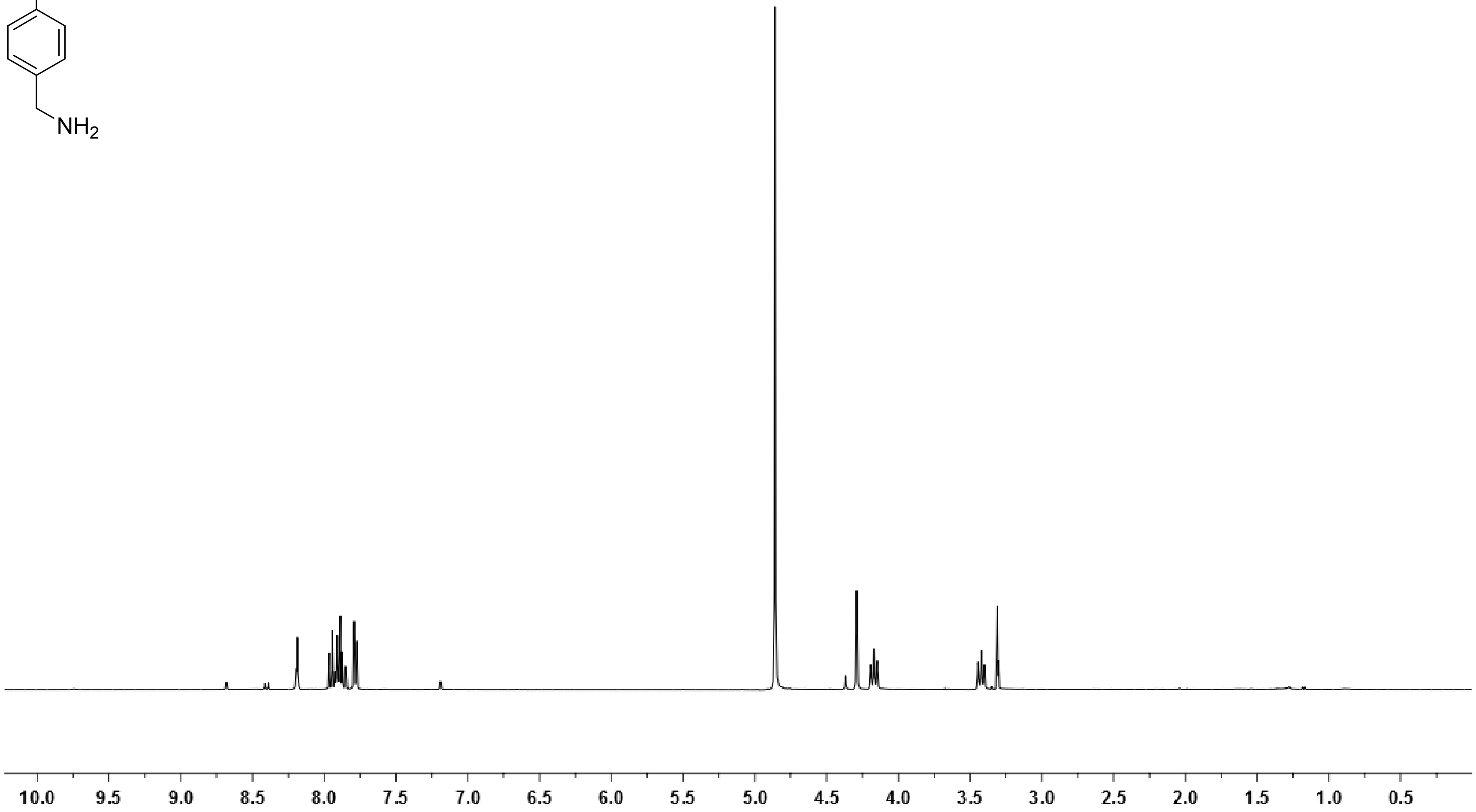

4-{8-Chloro-2,3-dihydro-1H-pyrrolo[3,2-c]quinolin-4-yl}benzylamine ***30***

^{13}C NMR (100.6 MHz, CD_3OD)

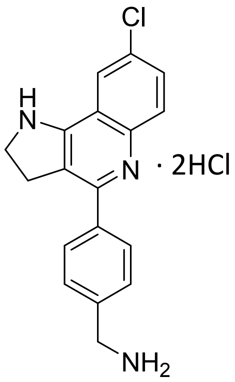

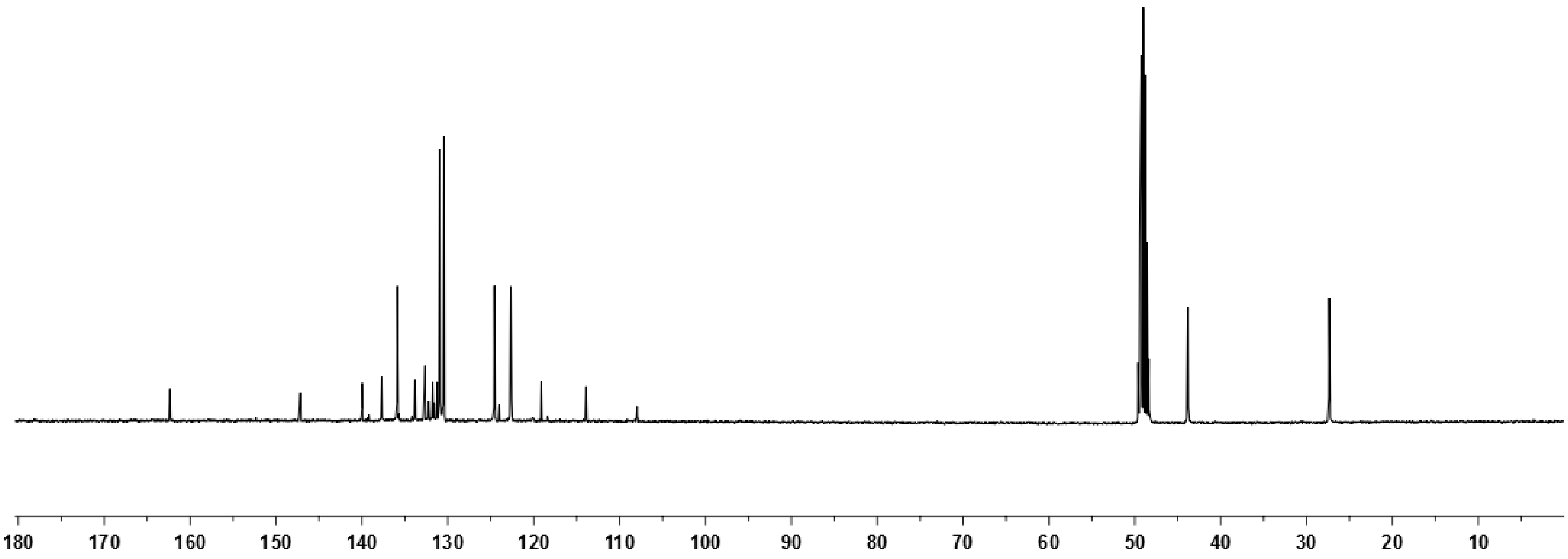

4-{9-Chloro-1,2,3,4-tetrahydrobenzo[h][1,6]naphthyridin-5-yl}benzylamine ***31***

^{1}H NMR (400 MHz , CD_3OD)

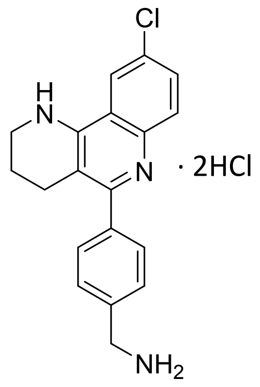

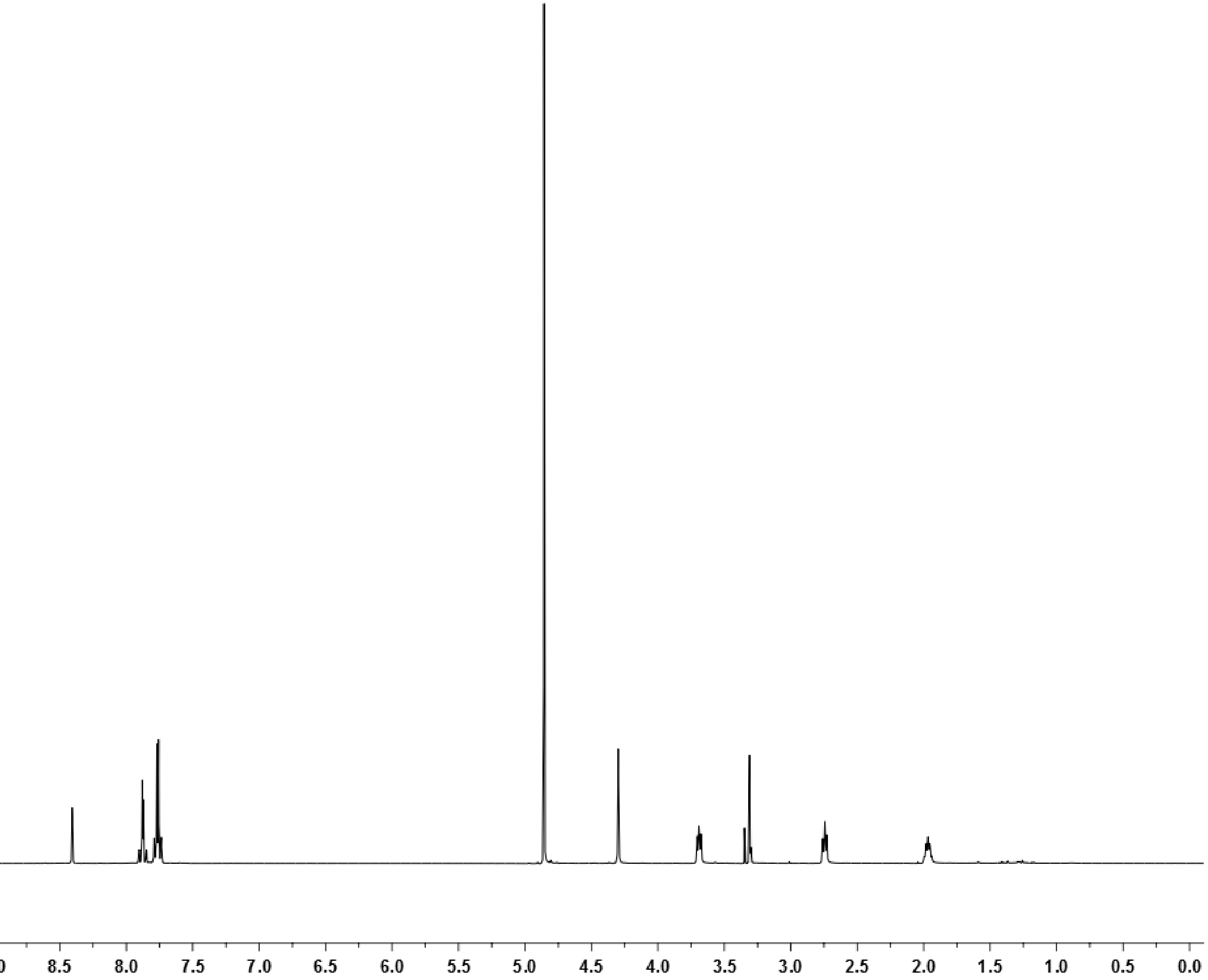

4-{9-Chloro-1,2,3,4-tetrahydrobenzo[h][1,6]naphthyridin-5-yl}benzylamine ***31***

^{13}C NMR (100.6 MHz, CD_3OD)

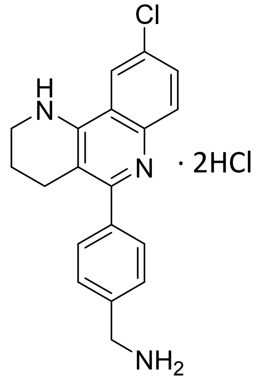

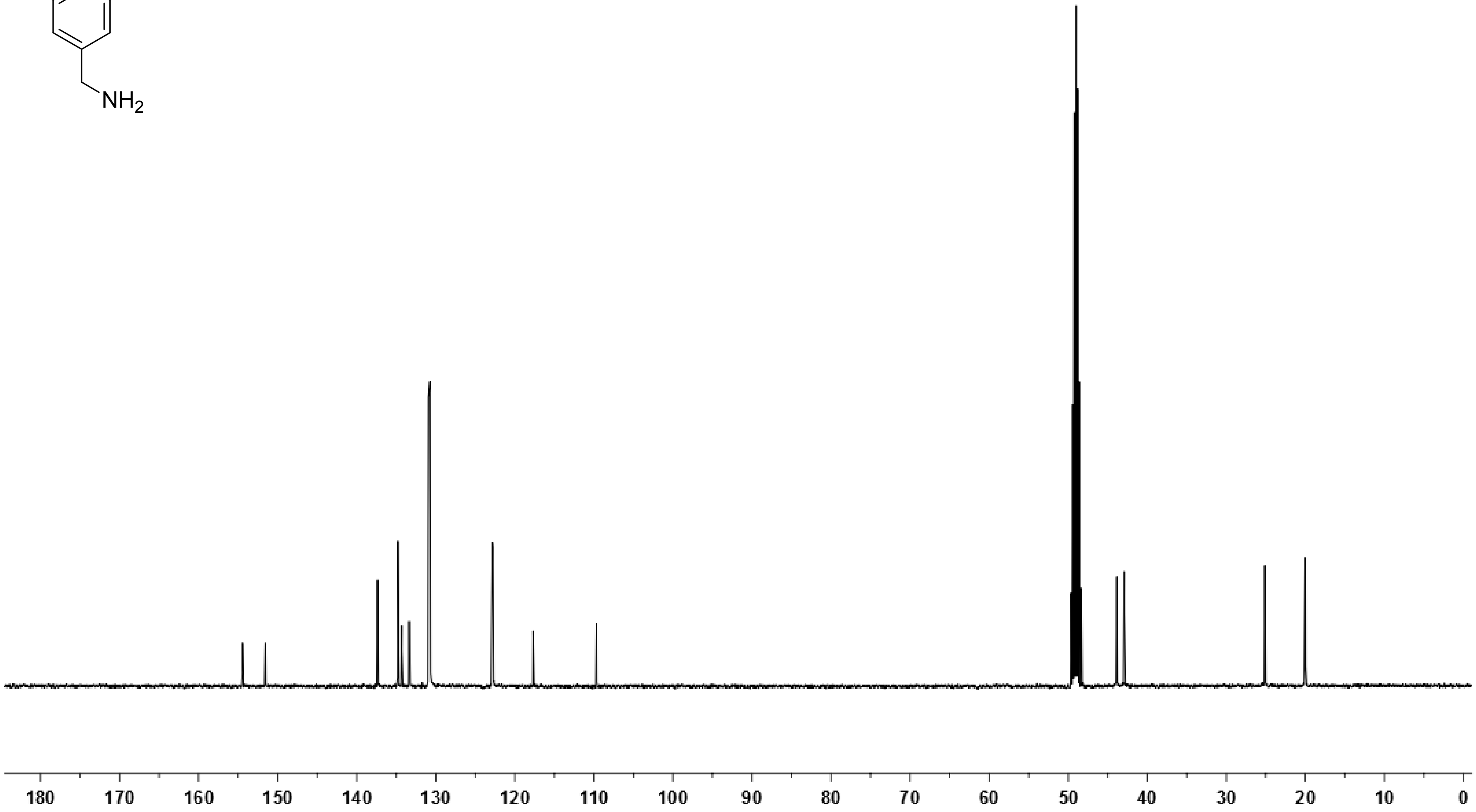

4-{10-Chloro-2,3,4,5-tetrahydro-1H-azepino[3,2-c]quinolin-6-yl}benzylamine ***32***

^{1}H NMR (400 MHz , CD_3OD)

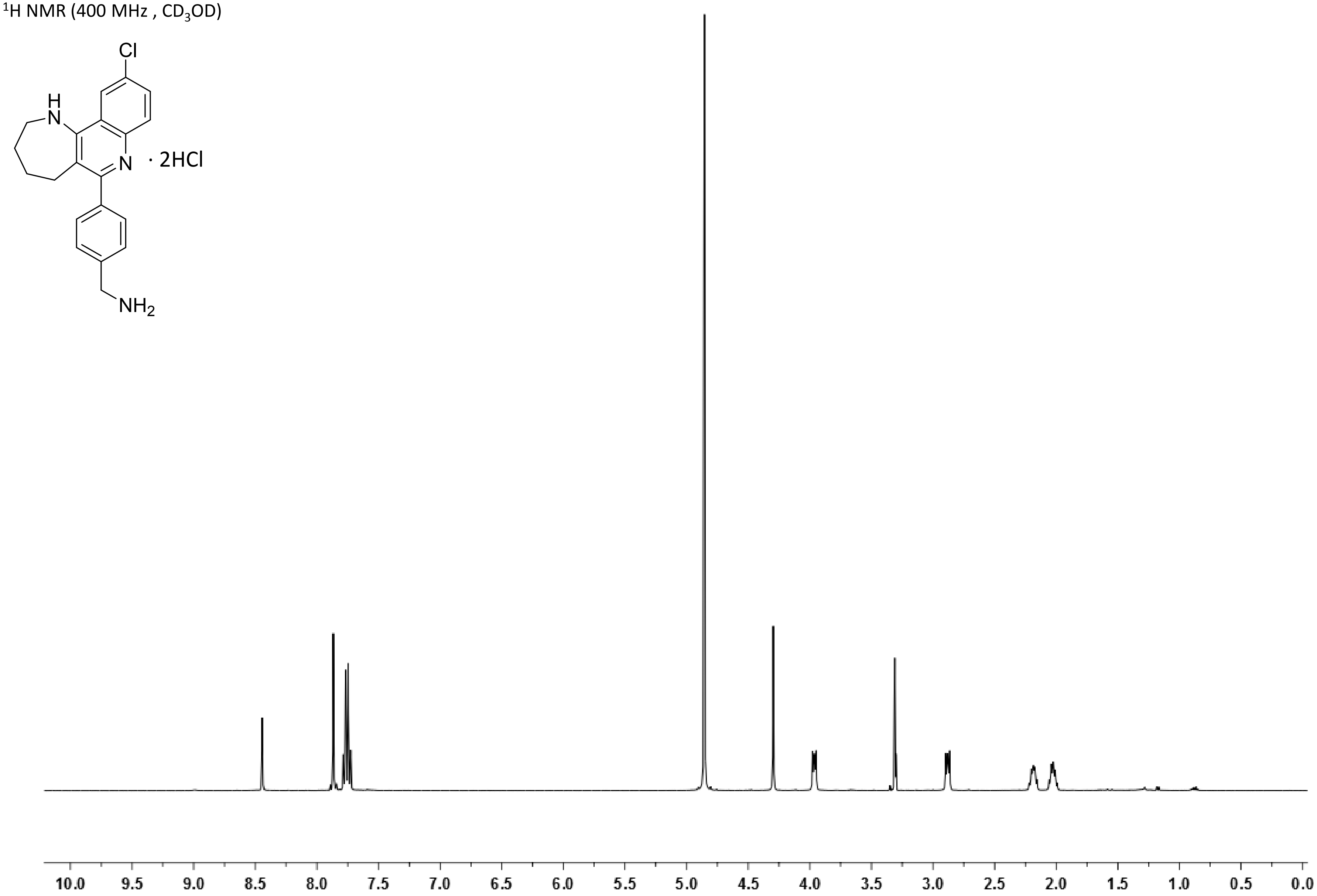

4-{10-Chloro-2,3,4,5-tetrahydro-1H-azepino[3,2-c]quinolin-6-yl}benzylamine ***32***

^{13}C NMR (100.6 MHz, CD_3OD)

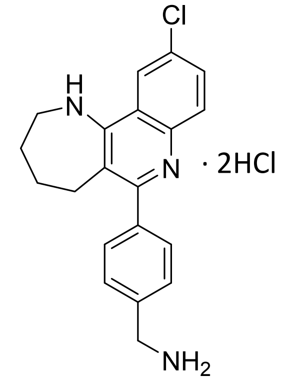

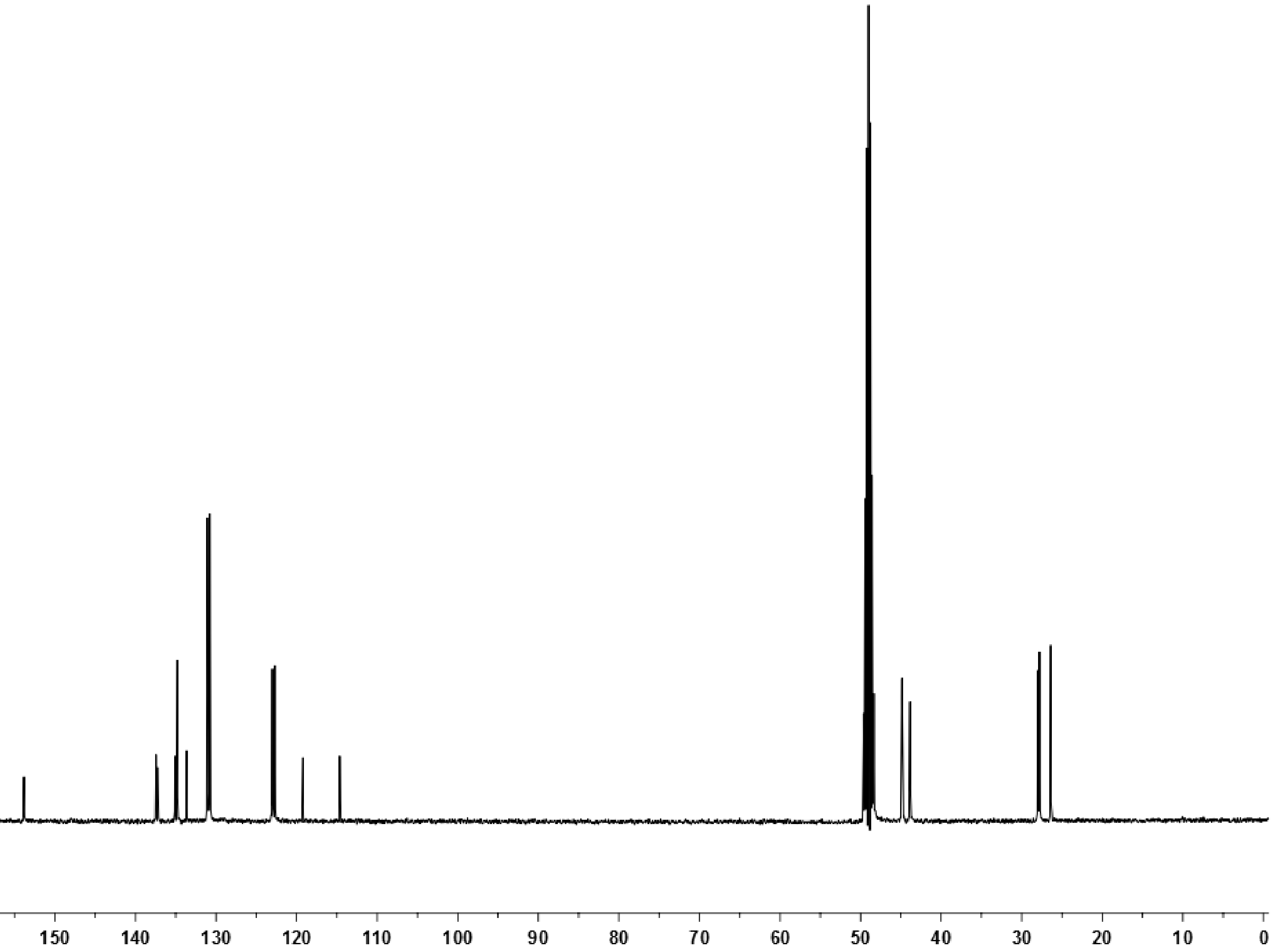

4-{8-Chloro-1,2,3,4-tetrahydrobenzo[h][1,6]naphthyridin-5-yl}benzylamine ***33***

^{1}H NMR (400 MHz , CD_3OD)

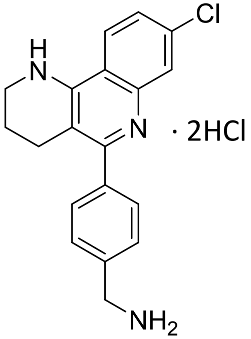

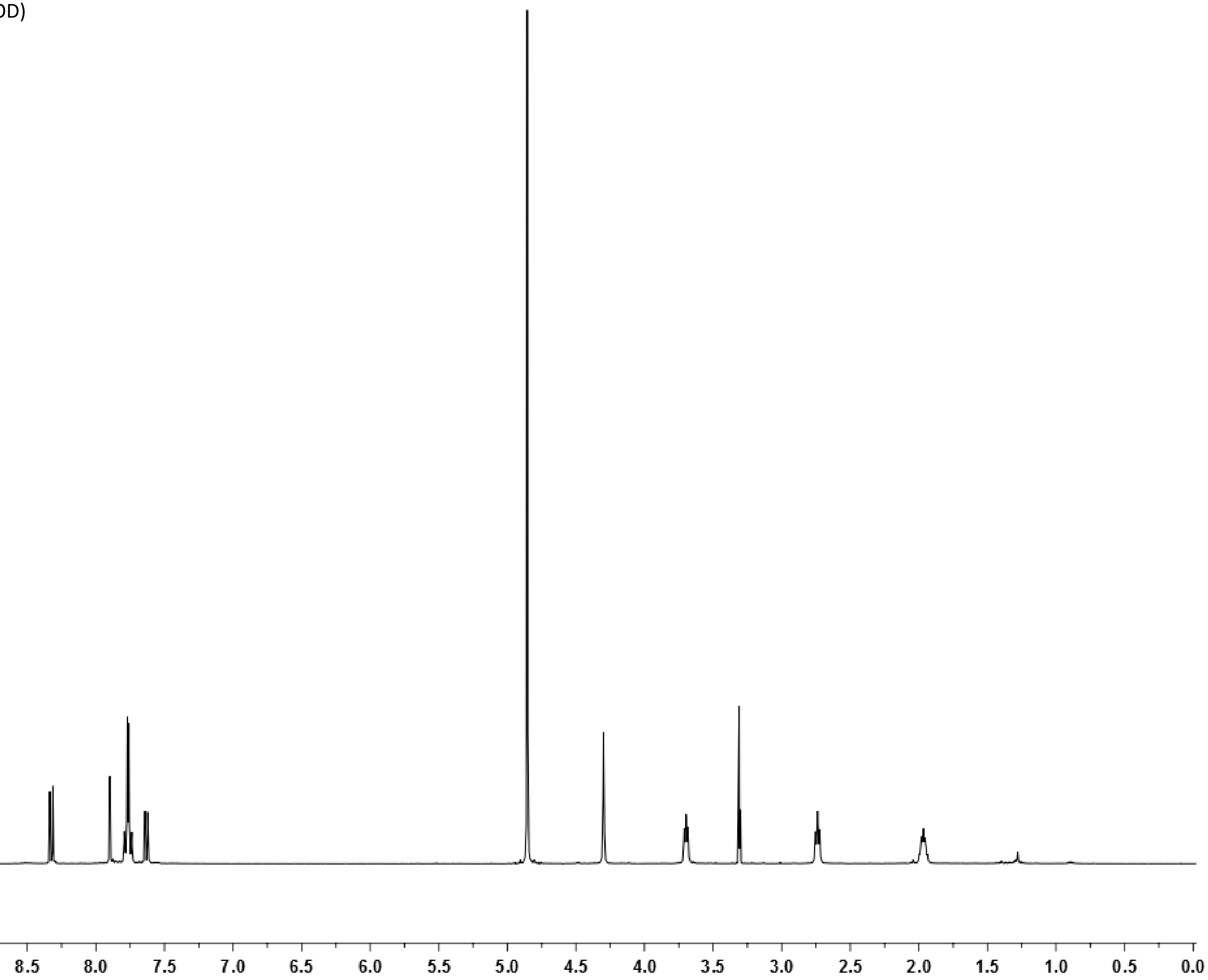

*4-{8-Chloro-1,2,3,4-tetrahydrobenzo[h][1,6]naphthyridin-5-yl}benzylamine **33***

^{13}C NMR (100.6 MHz, CD_3OD)

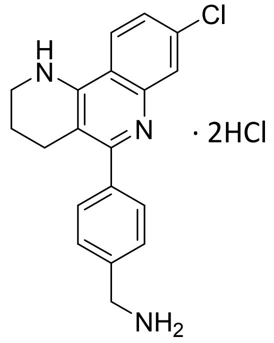

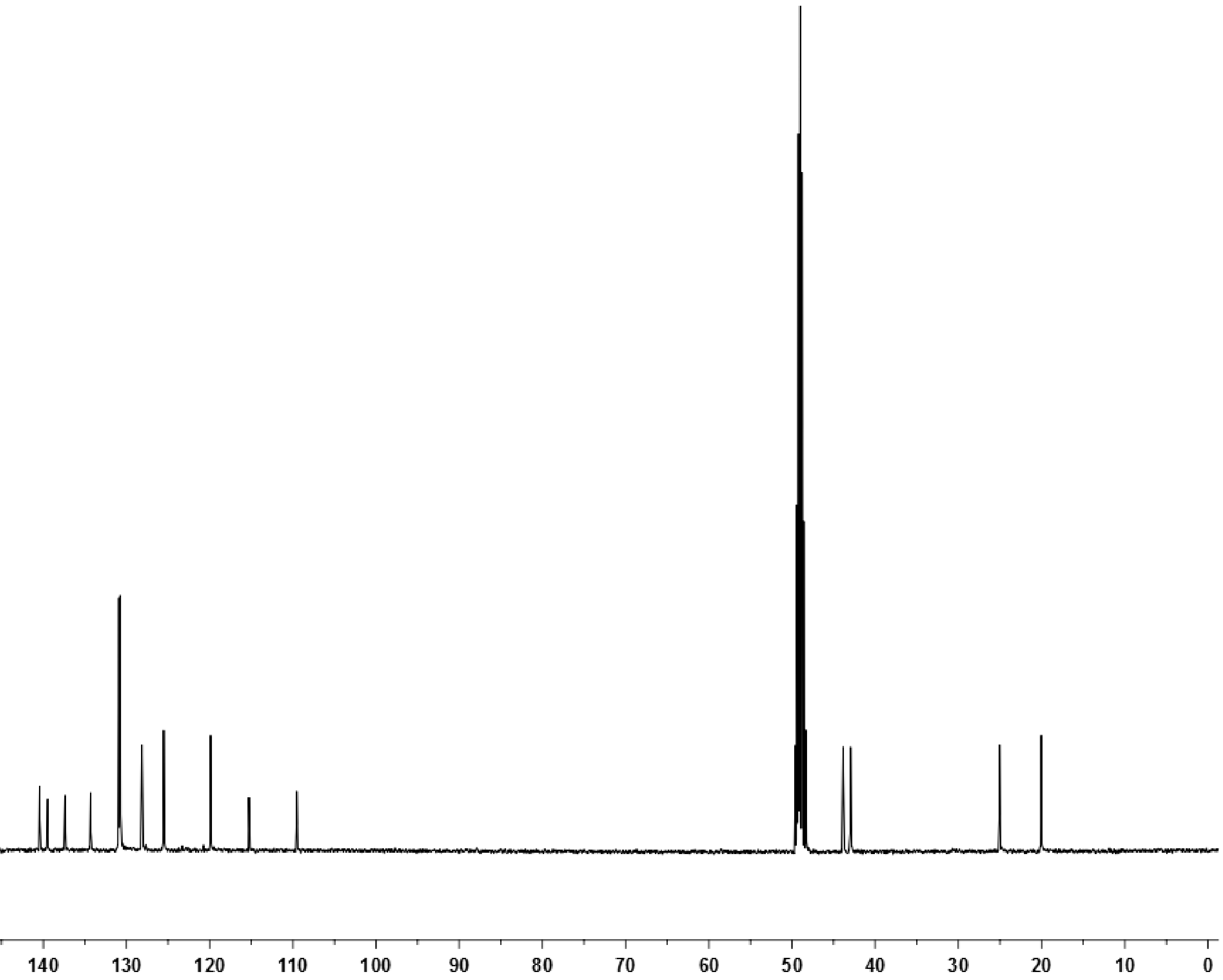

4-[3-(3-Aminopropyl)-7-chloroquinolin-2-yl]benzonitrile ***35***

^{1}H NMR (400 MHz , CD_3OD)

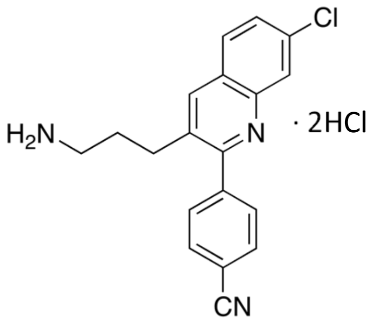

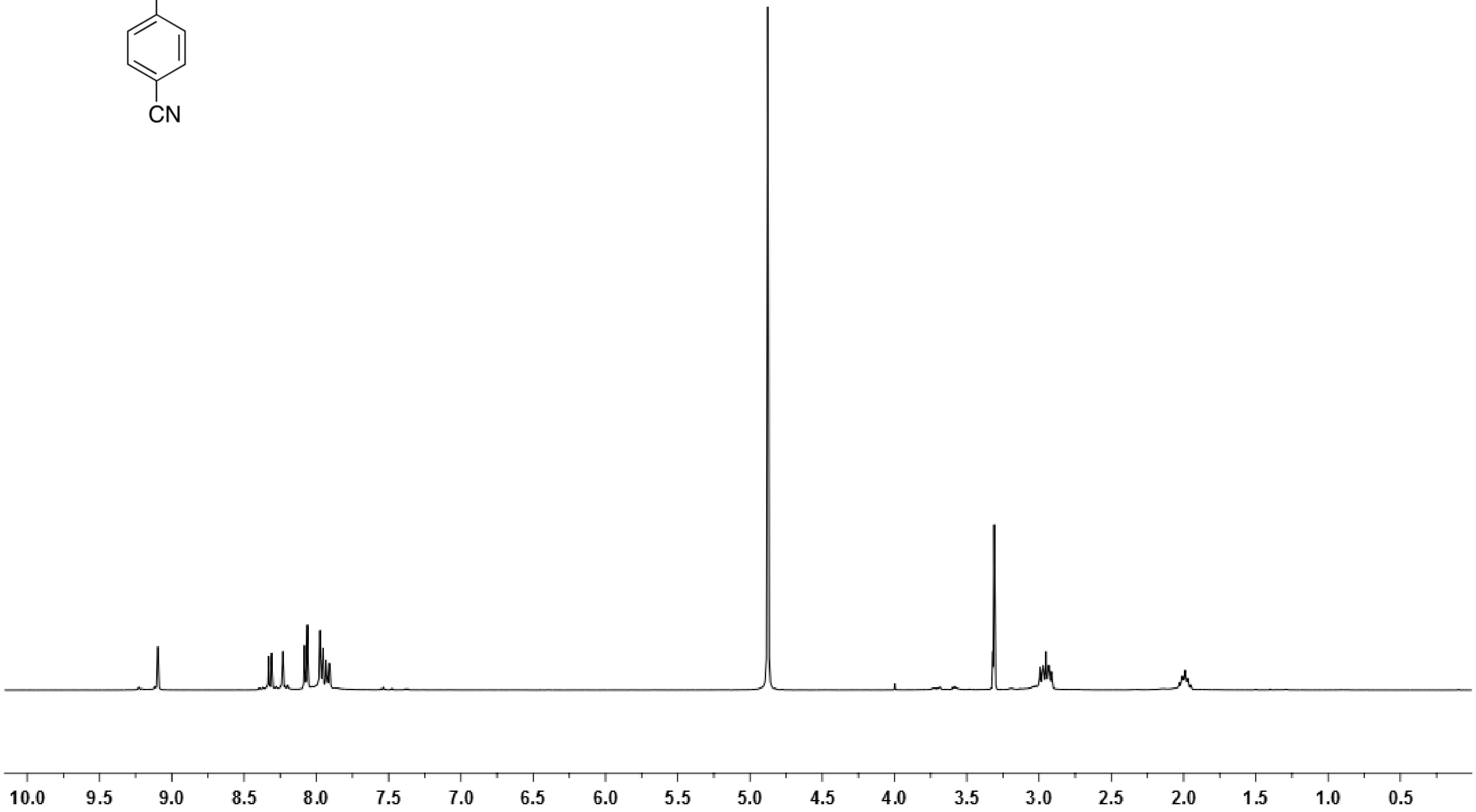

4-[3-(3-Aminopropyl)-7-chloroquinolin-2-yl]benzonitrile ***35***

^{13}C NMR (100.6 MHz, CD_3OD)

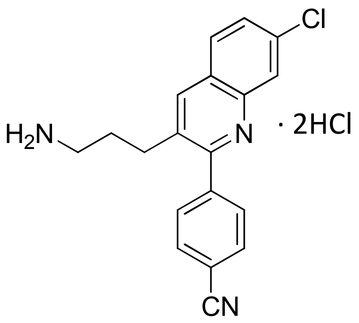

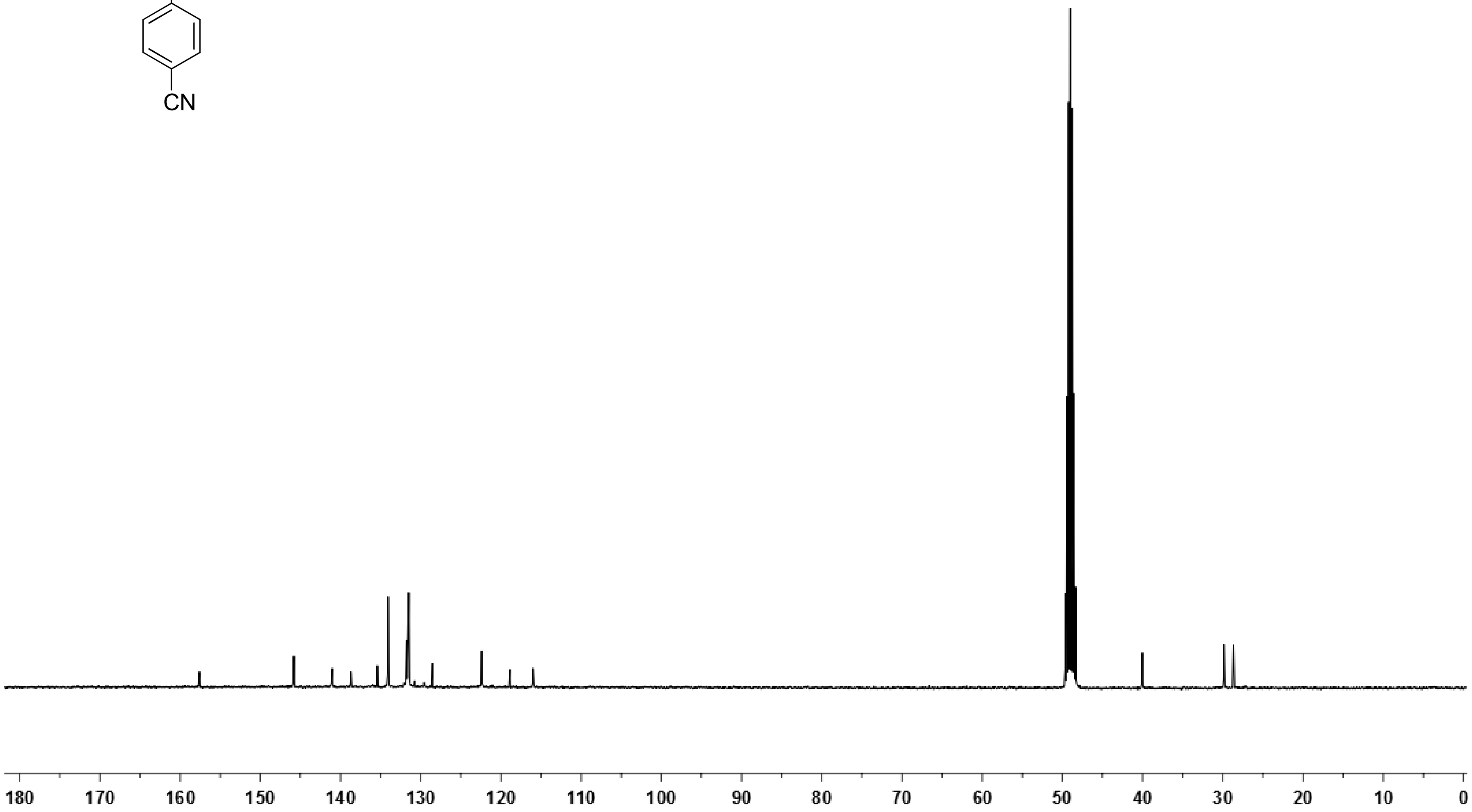

4-{9-Chloro-3,4,4a,5,6,10b-hexahydro-2H-pyrano[3,2-c]quinolin-5-yl}benzonitrile ***37***

^{1}H NMR (400 MHz , $CDCl_3$)

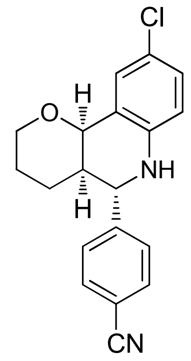

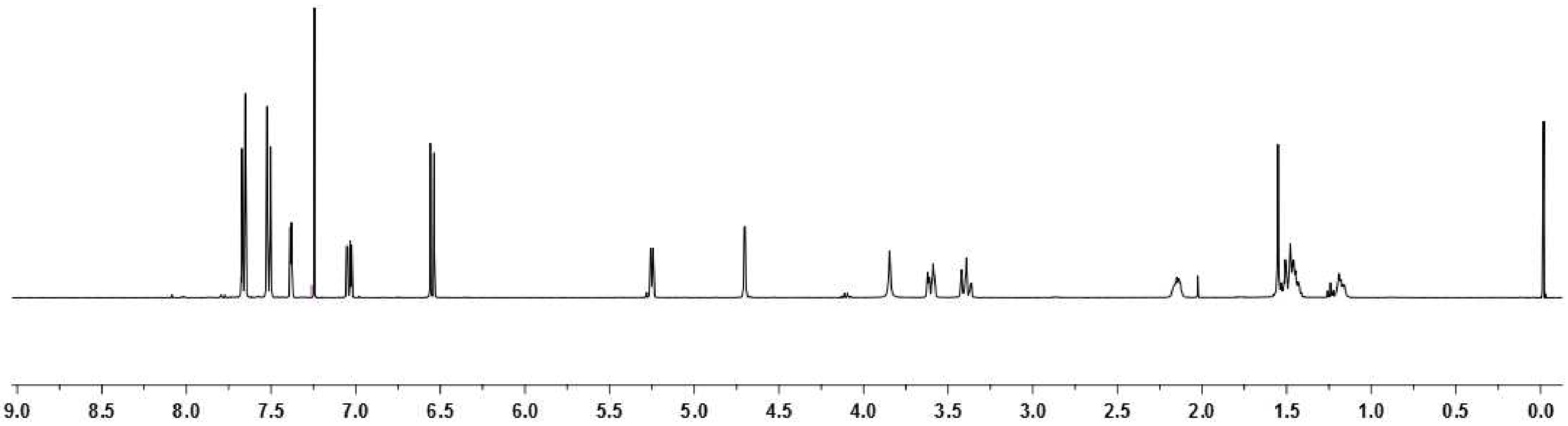

4-{9-Chloro-3,4,4a,5,6,10b -hexahydro-2H-pyrano[3,2-c]quinolin-5-yl}benzonitrile ***37***

^{13}C NMR (100.6 MHz, $CDCl_3$)

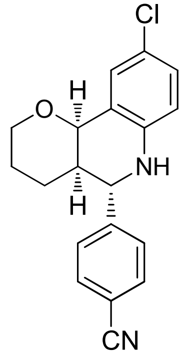

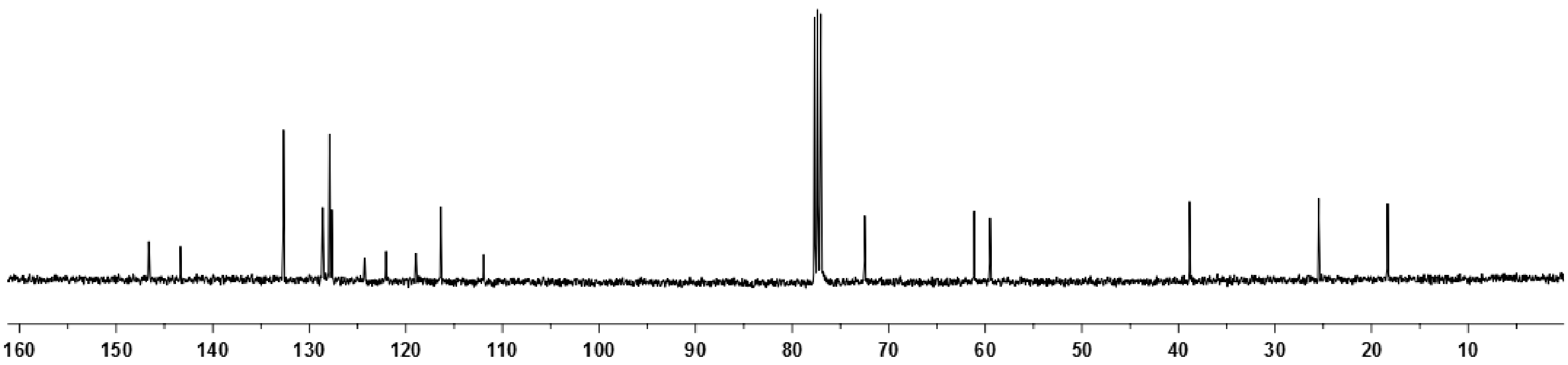

4-{9-Chloro-3,4-dihydro-2H-pyrano[3,2-c]quinolin-5-yl}benzonitrile ***38***

^{1}H NMR (400 MHz , CD_3OD)

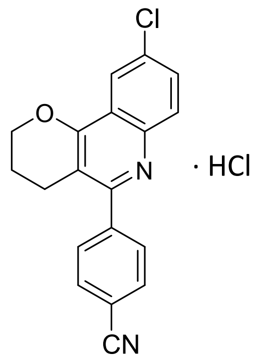

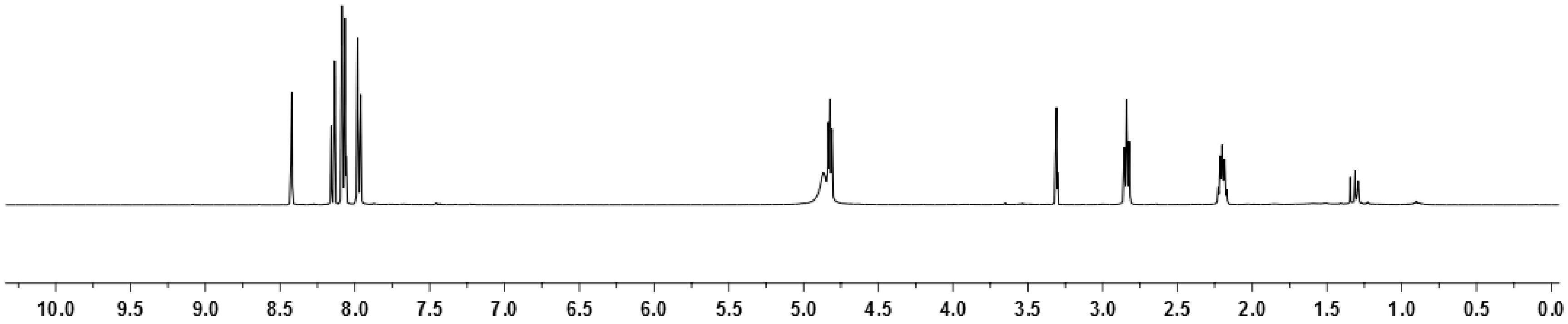

*4-{9-Chloro-3,4-dihydro-2H-pyrano[3,2-c]quinolin-5-yl}benzonitrile **38***

^{13}C NMR (100.6 MHz, CD_3OD)

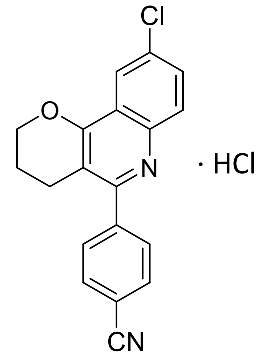

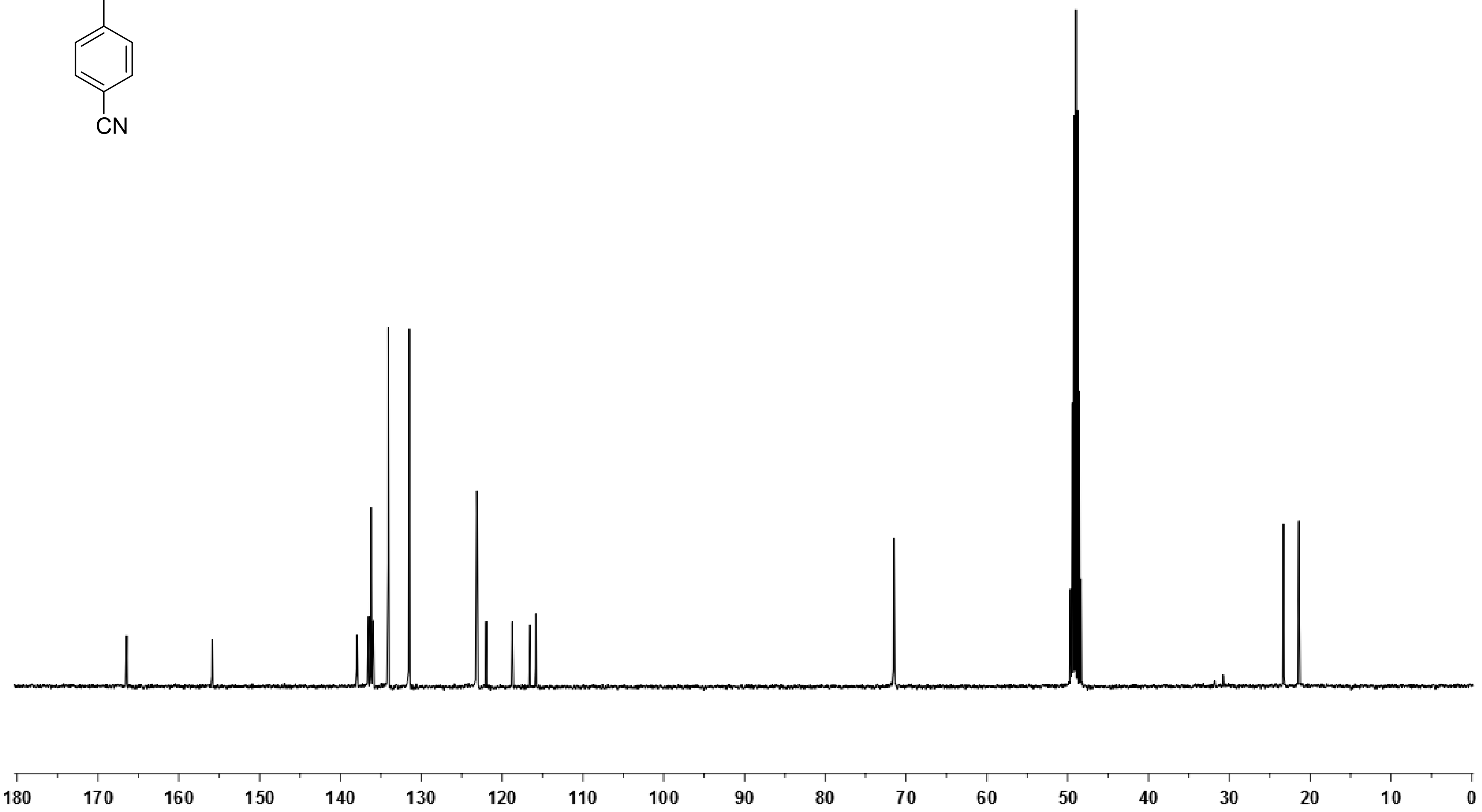

4-{9-Chloro-3,4-dihydro-2H-pyrano[3,2-c]quinolin-5-yl}benzylamine ***39***

^{1}H NMR (400 MHz , CD_3OD)

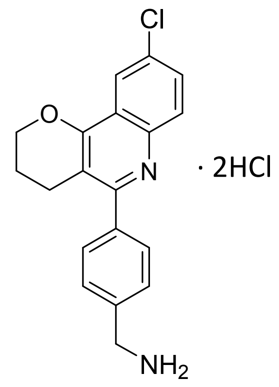

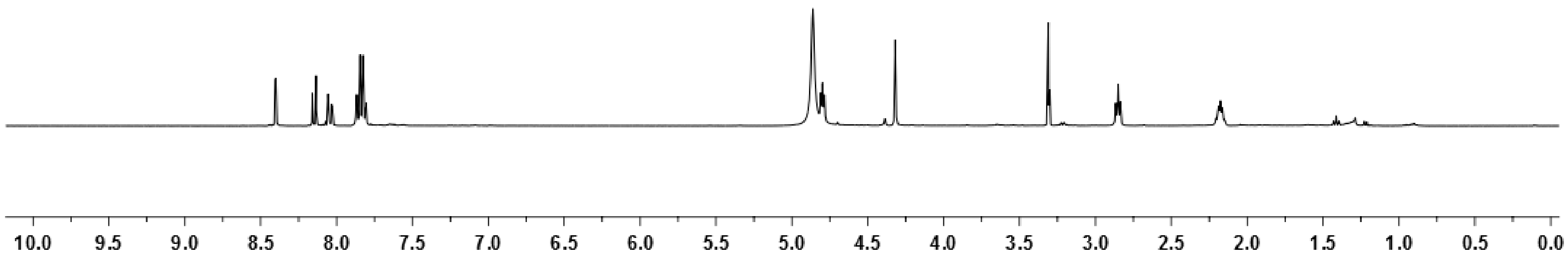

4-{9-Chloro-3,4-dihydro-2H-pyrano[3,2-c]quinolin-5-yl}benzylamine ***39***

^{13}C NMR (100.6 MHz, CD_3OD)

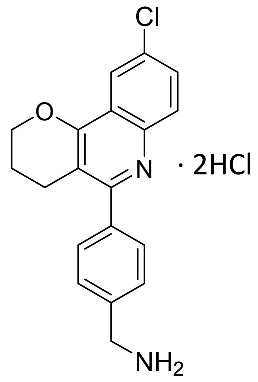

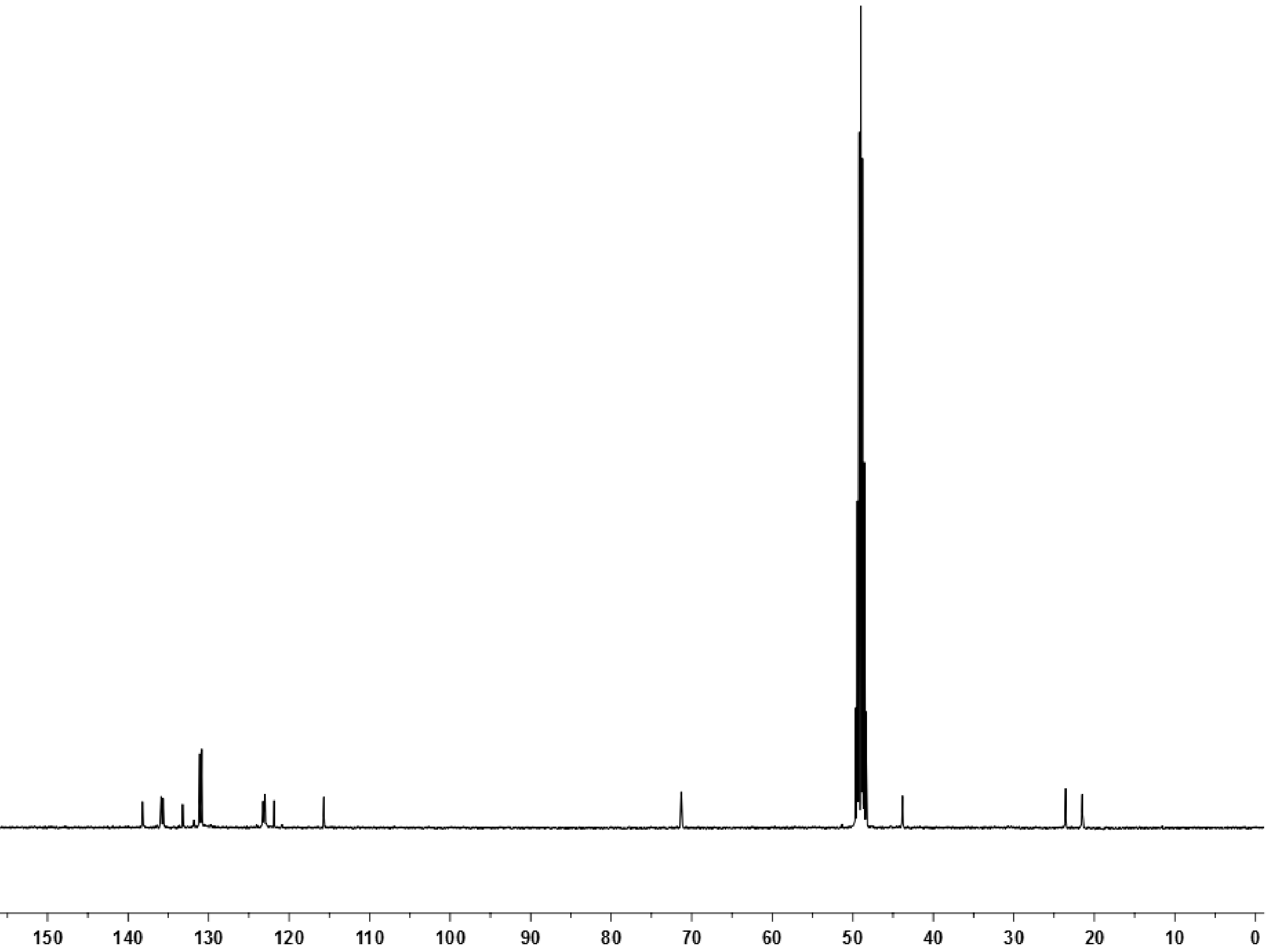

*9-Chloro-5-(2-furyl)-3,4-dihydro-2H-pyrano[3,2-c]quinoline **42***

^1H NMR (400 MHz , CD_3OD)

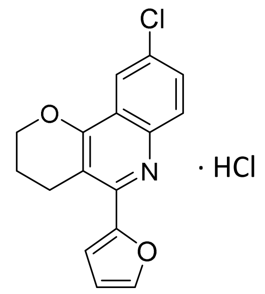

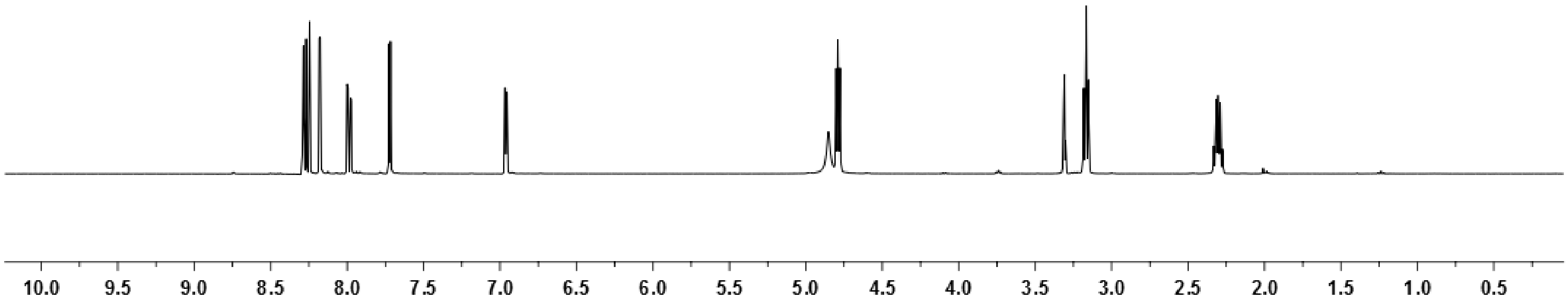

9-Chloro-5-(2-furyl)-3,4-dihydro-2H-pyrano[3,2-c]quinoline ***42***

^{13}C NMR (100.6 MHz, CD_3OD)

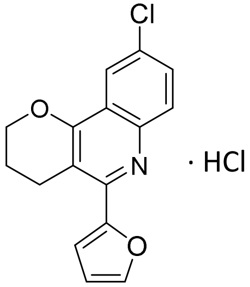

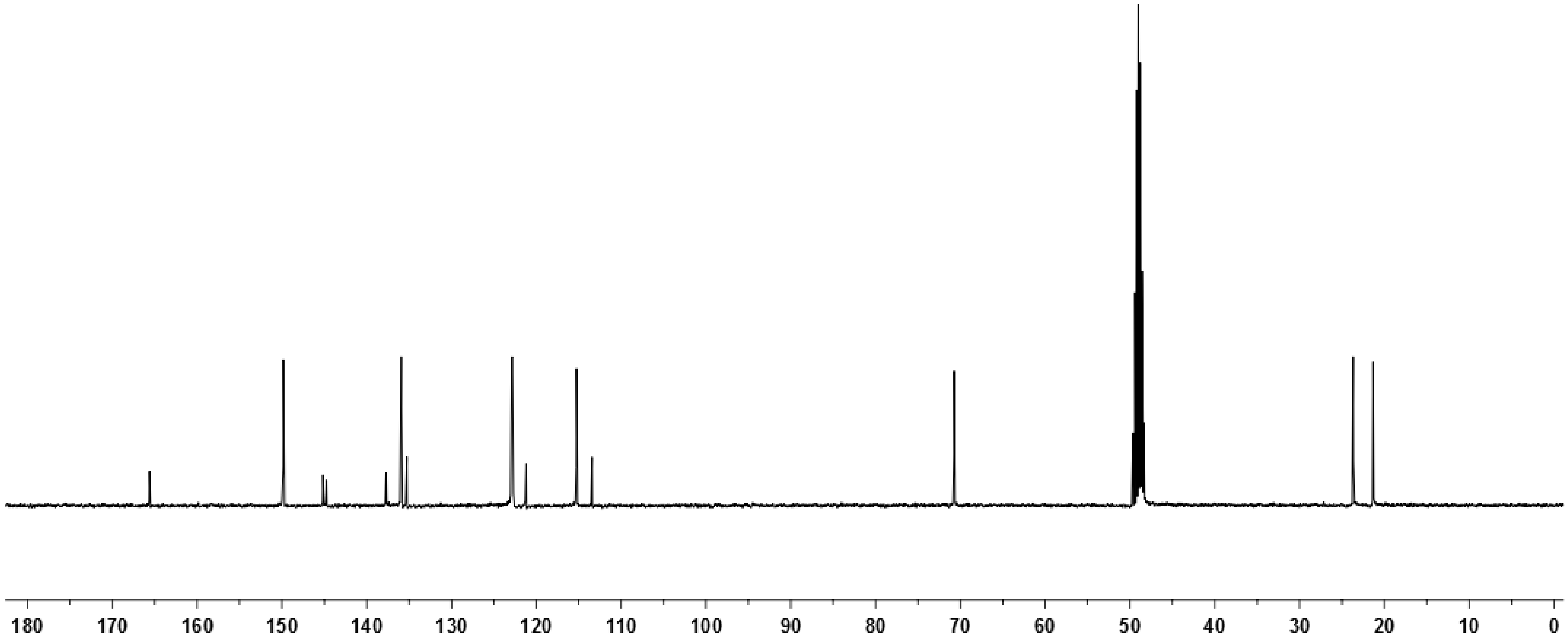

9-Chloro-3,4-dihydro-5-(2-thienyl)-2H-pyrano[3,2-c]quinoline ***43***

^{1}H NMR (400 MHz , CD_3OD)

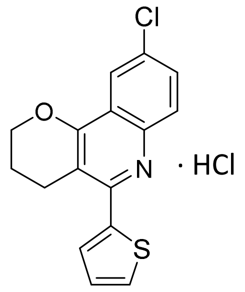

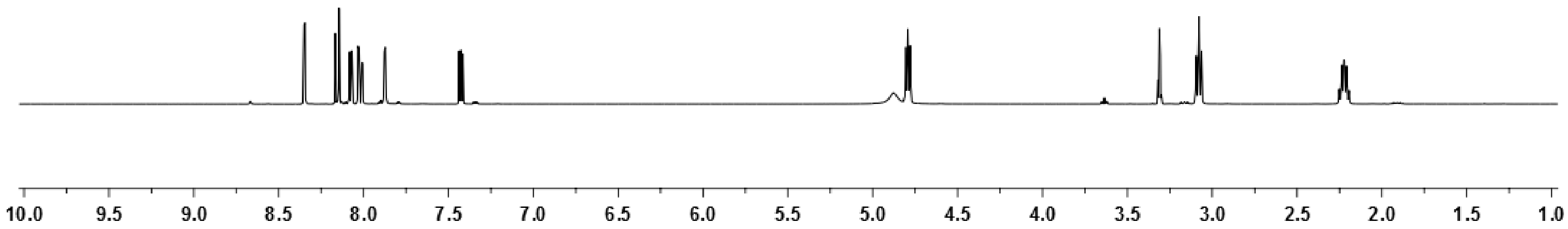

9-Chloro-3,4-dihydro-5-(2-thienyl)-2H-pyrano[3,2-c]quinoline ***43***

^{13}C NMR (100.6 MHz, CD_3OD)

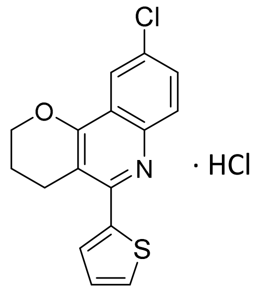

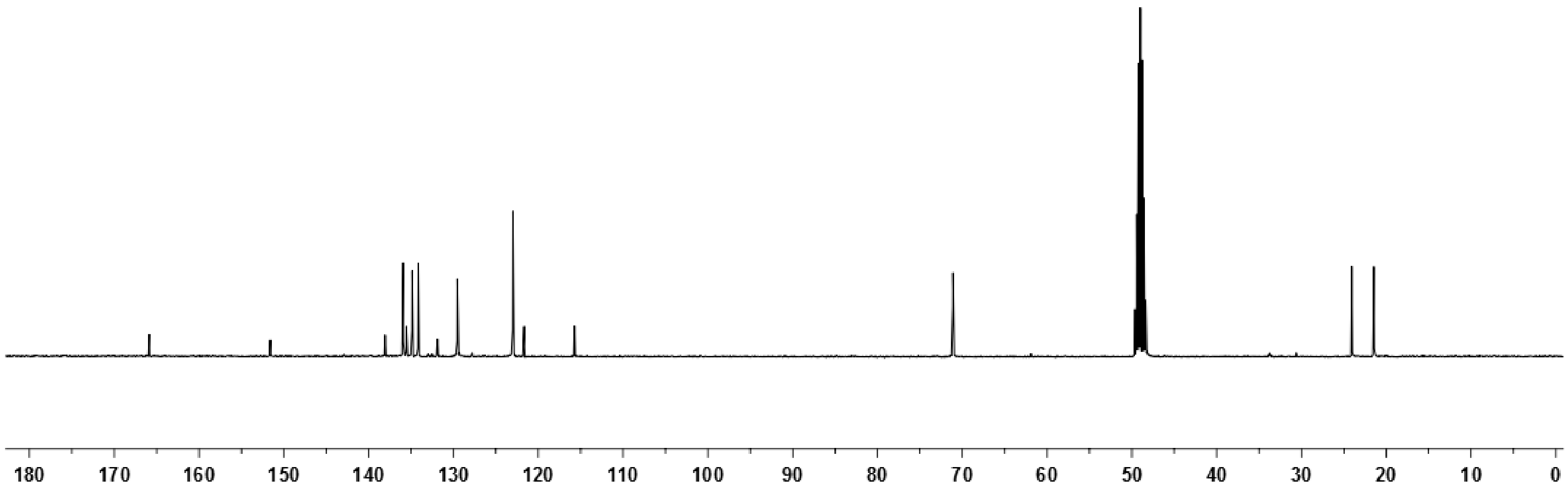

3-[6-Chloro-2-(2-furyl)quinolin-3-yl]-1-propanol **44**

^{1}H NMR (400 MHz , CD_3OD)

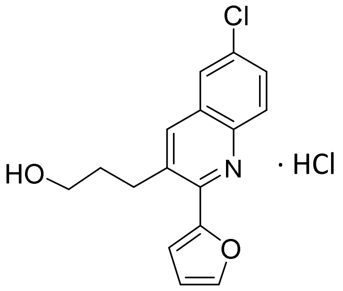

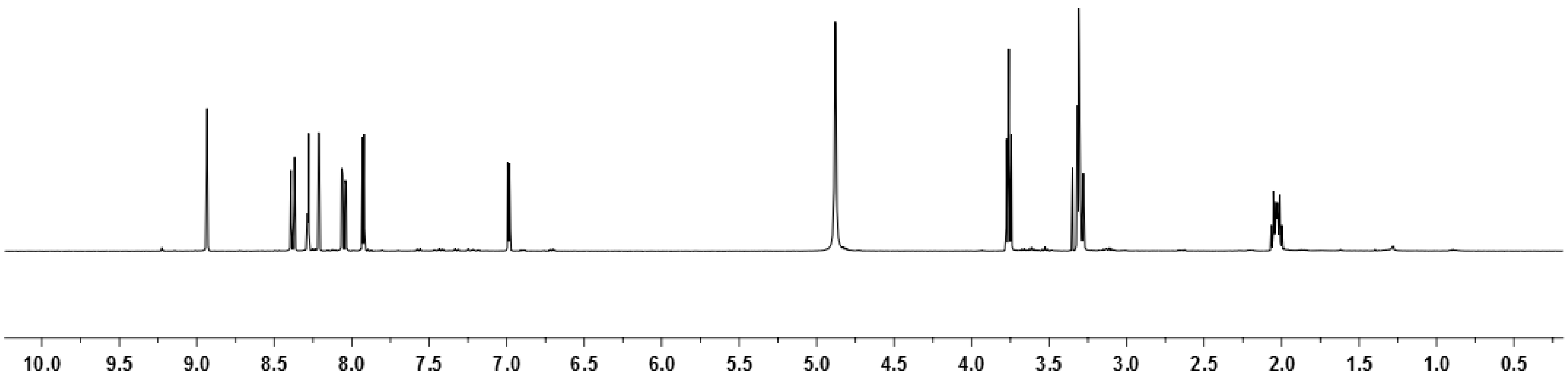

3-[6-Chloro-2-(2-furyl)quinolin-3-yl]-1-propanol **44**

^{13}C NMR (100.6 MHz, CD_3OD)

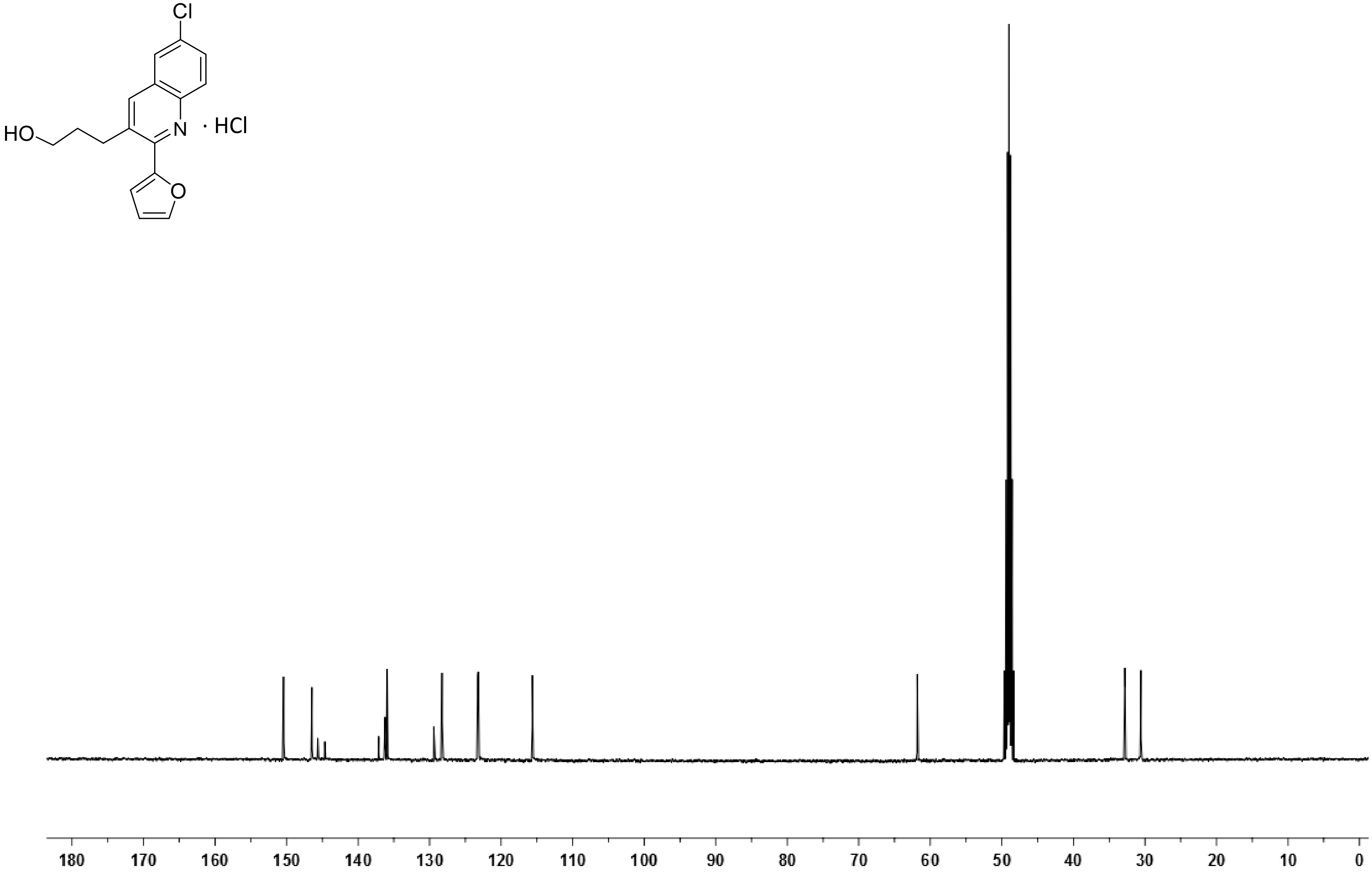

3-[6-Chloro-2-(2-thienyl)quinolin-3-yl]-1-propanol **45**

^{1}H NMR (400 MHz , CD_3OD)

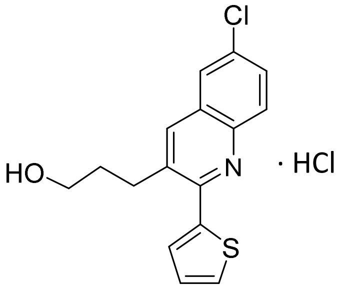

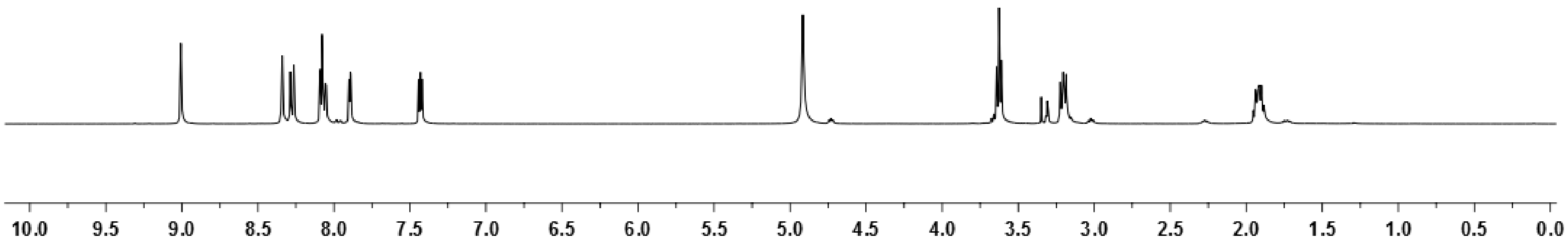

3-[6-Chloro-2-(2-thienyl)quinolin-3-yl]-1-propanol **45**

^{13}C NMR (100.6 MHz, CD_3OD)

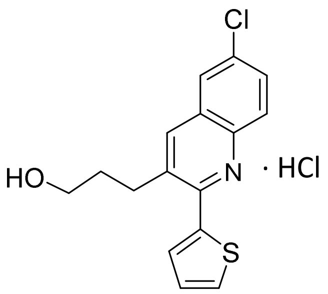

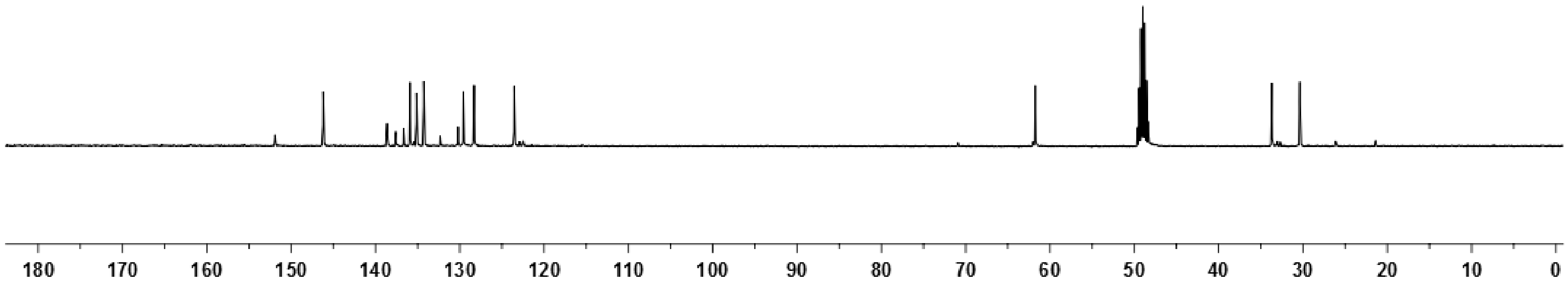

3-[6-Chloro-2-(2-thienyl)quinolin-3-yl]propanenitrile ***47***

^{1}H NMR (400 MHz , CD_3OD)

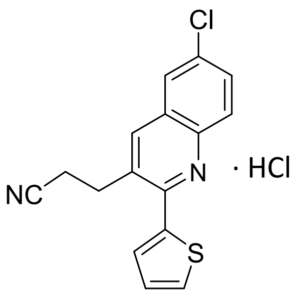

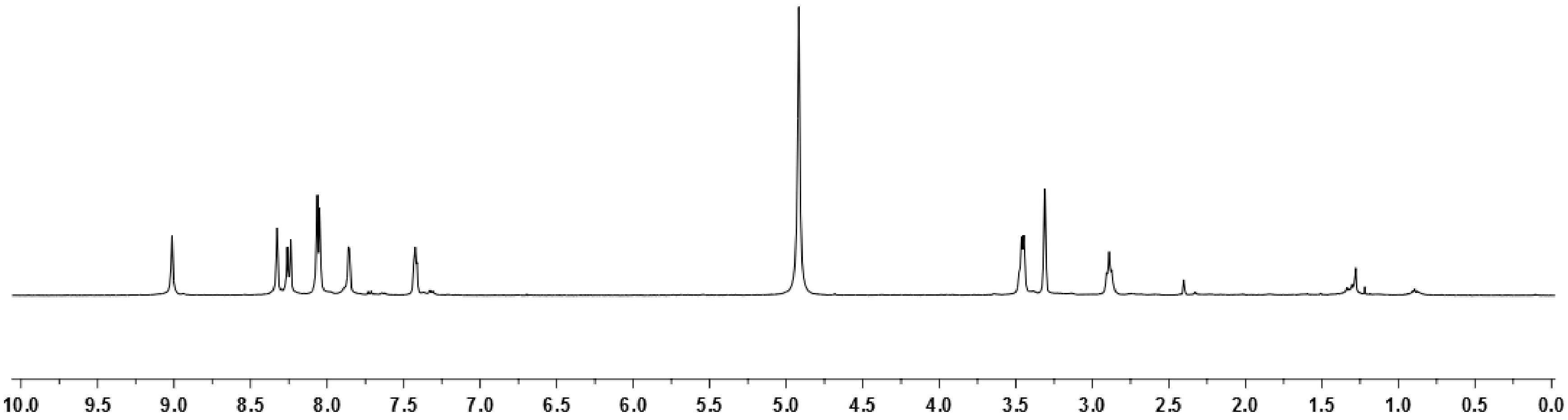

3-[6-Chloro-2-(2-thienyl)quinolin-3-yl]propanenitrile ***47***

^{13}C NMR (100.6 MHz, CD_3OD)

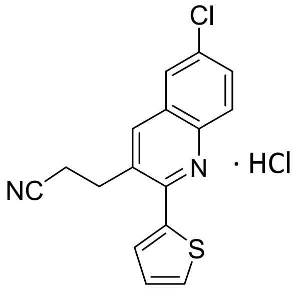

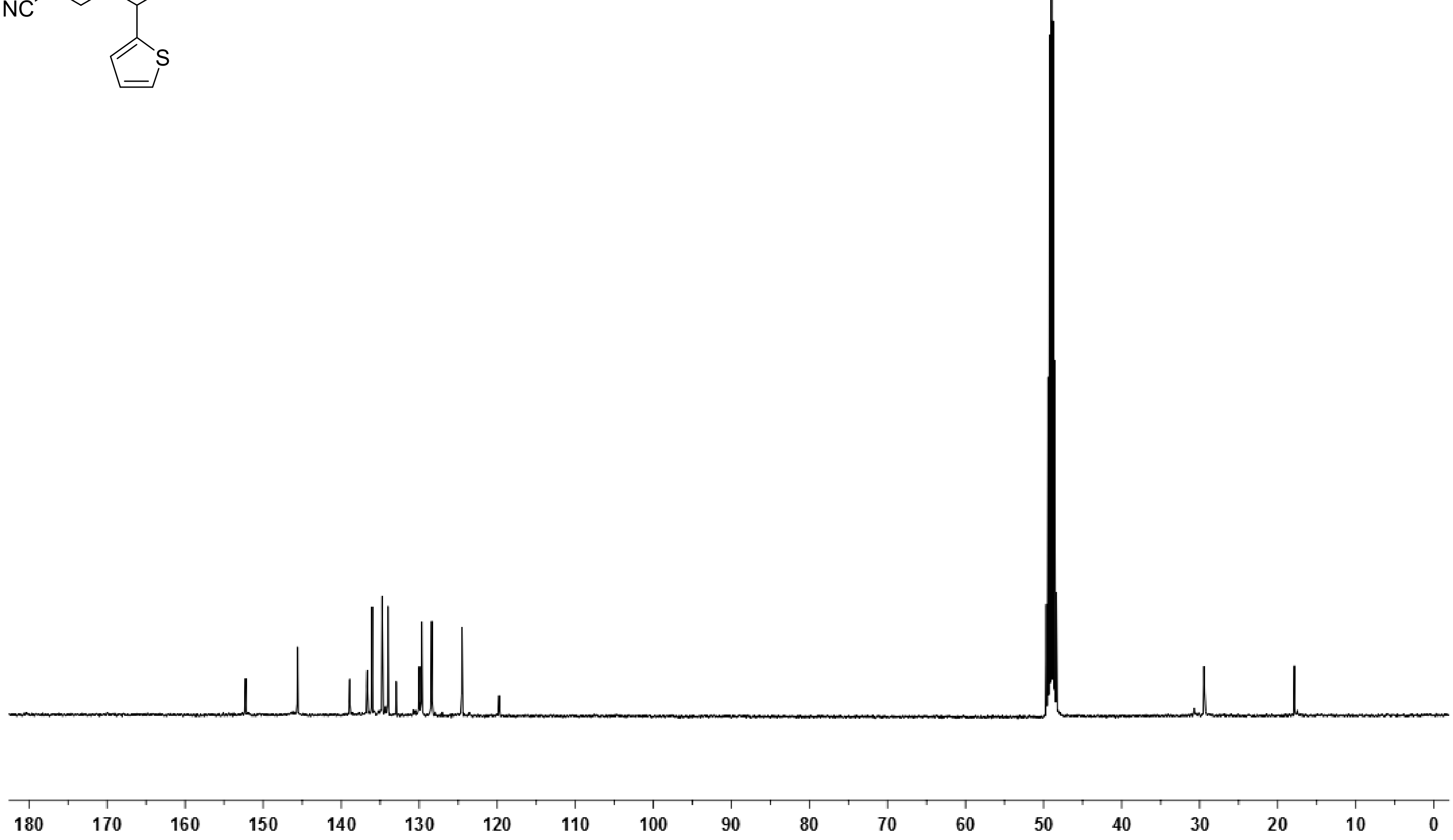

N-{3-[6-Chloro-2-(2-thienyl)quinolin-3-yl]propyl}pyrrolidine ***49***

^{1}H NMR (400 MHz , CD_3OD)

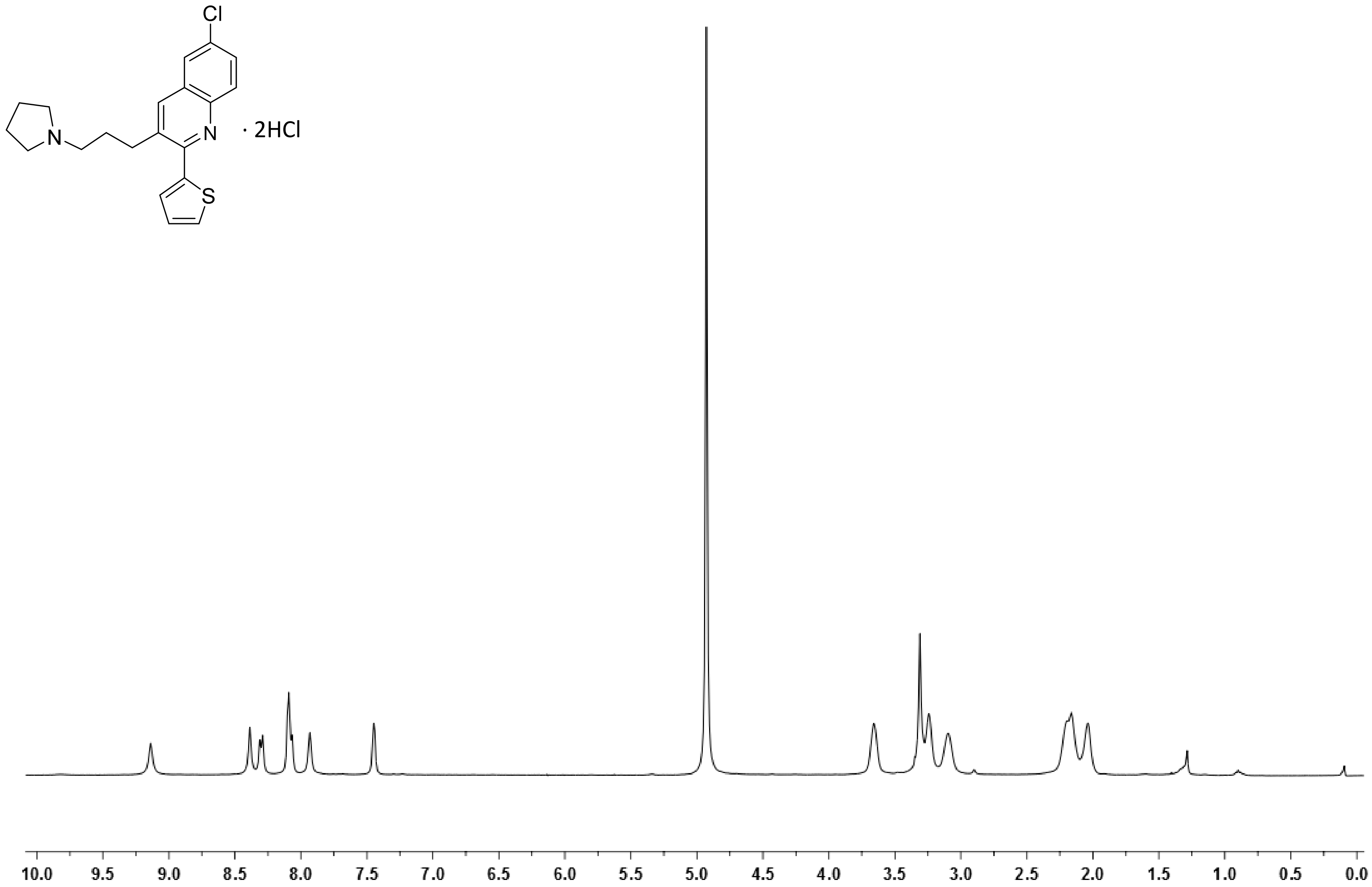

N-{3-[6-Chloro-2-(2-thienyl)quinolin-3-yl]propyl}pyrrolidine ***49***

^{13}C NMR (100.6 MHz, CD_3OD)

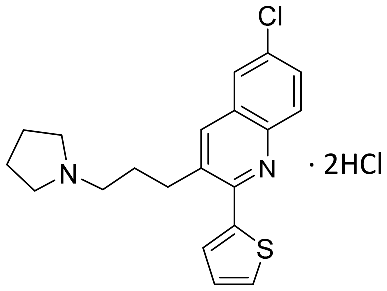

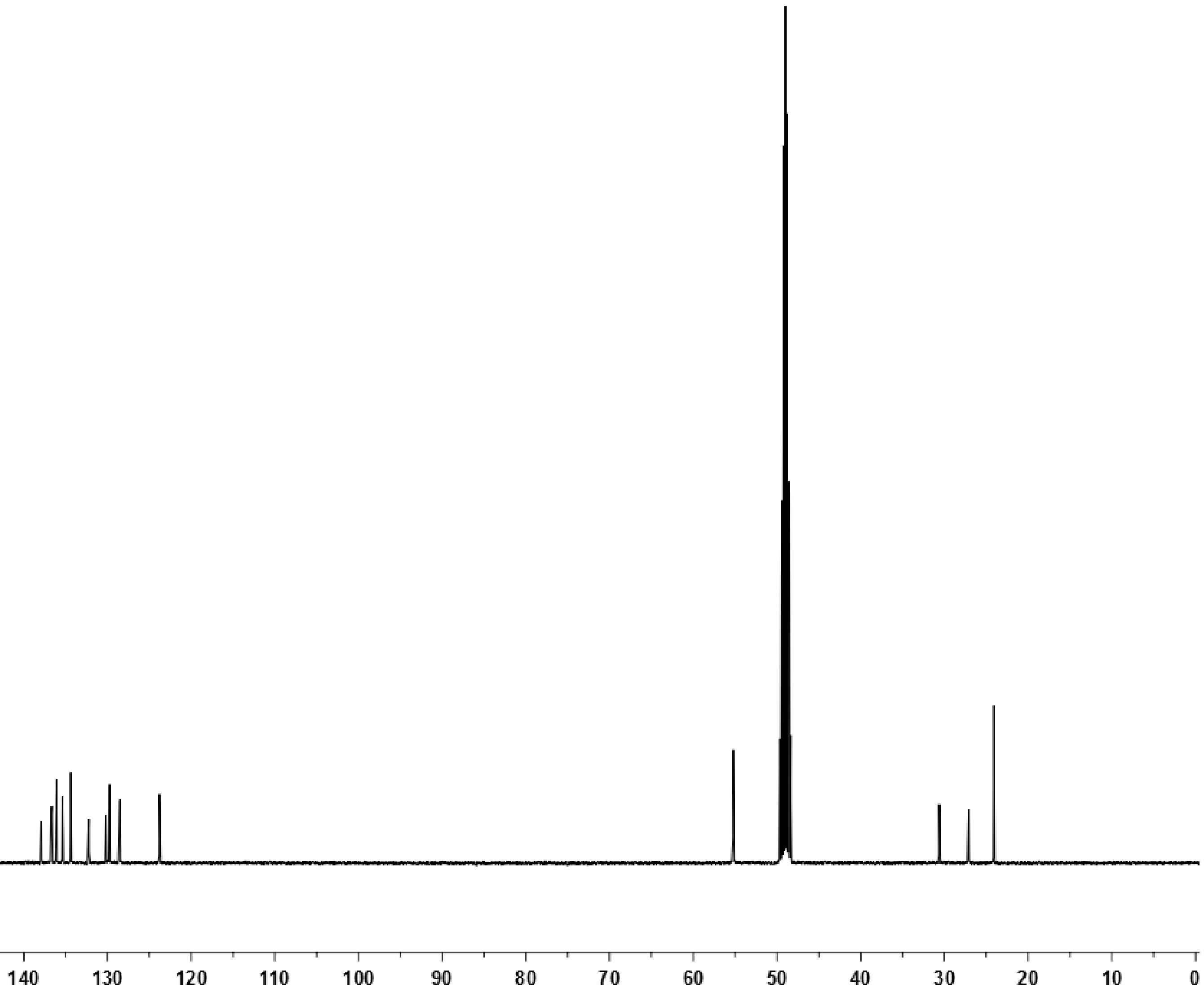

N-{3-[6-Chloro-2-(2-thienyl)quinolin-3-yl]propyl}-N,N-diethylamine ***50***

^{1}H NMR (400 MHz , CD_3OD)

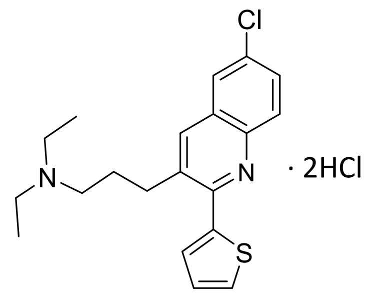

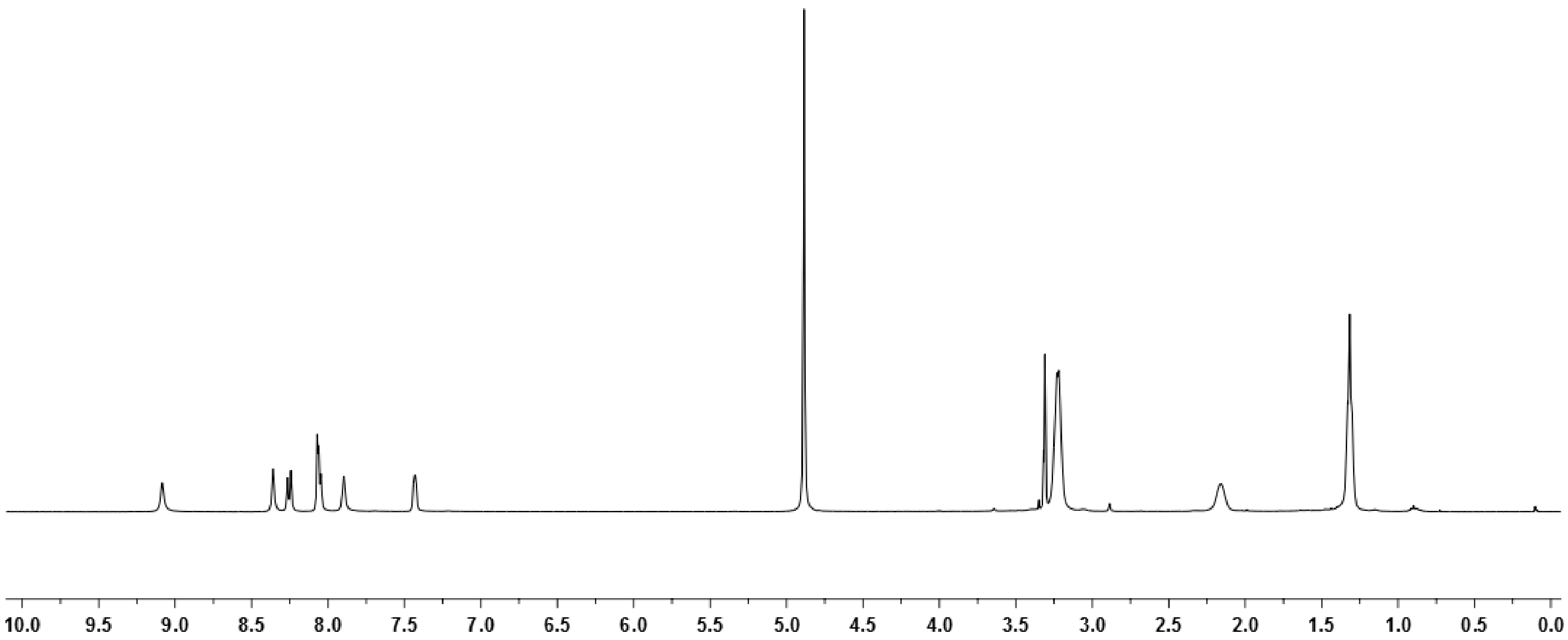

N-{3-[6-Chloro-2-(2-thienyl)quinolin-3-yl]propyl}-N,N-diethylamine ***50***

^{13}C NMR (100.6 MHz, CD_3OD)

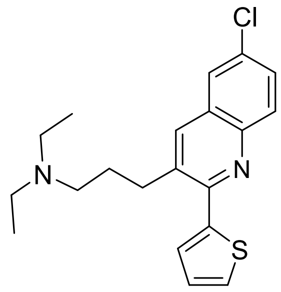

· 2HCl

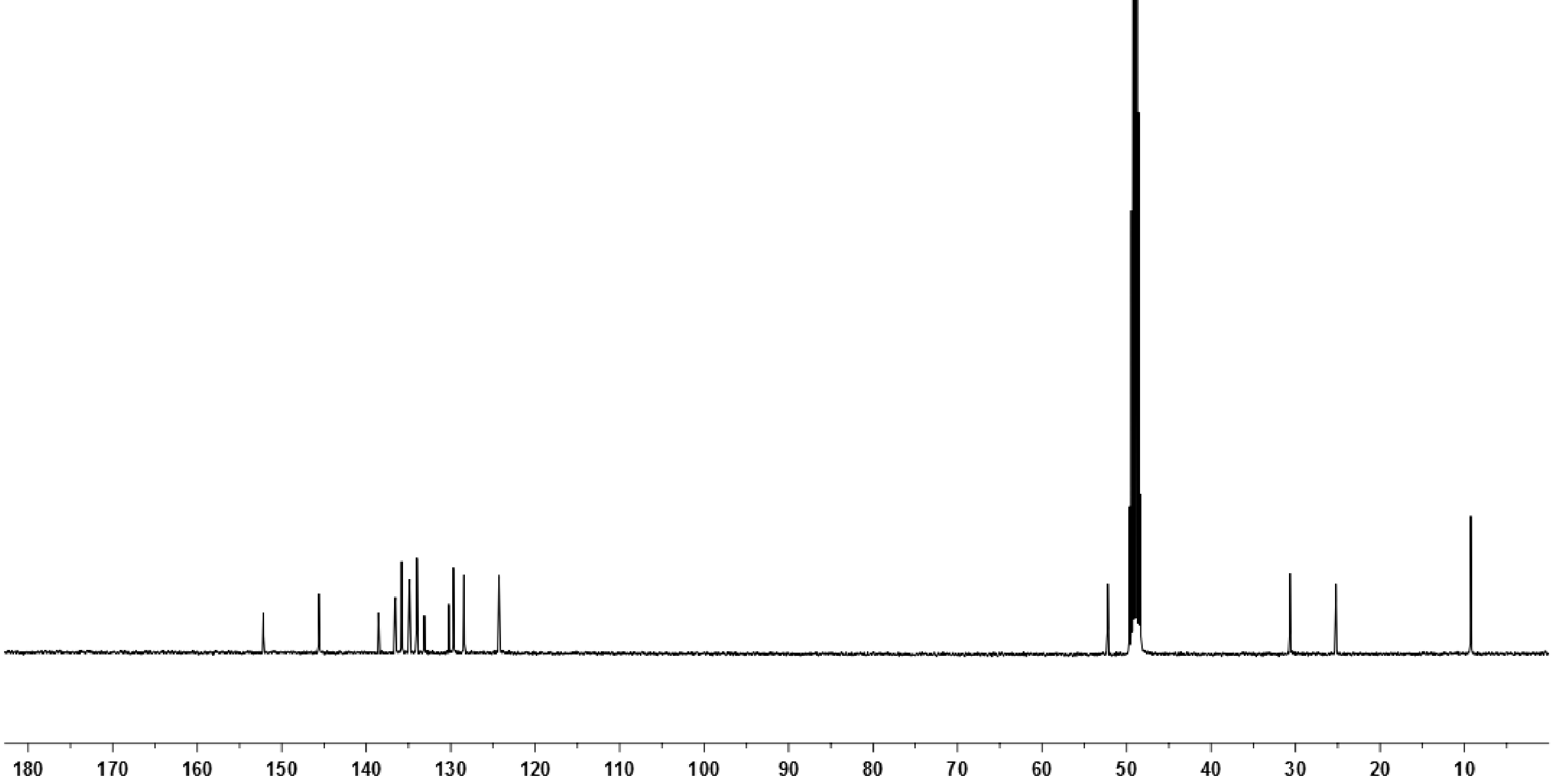

Supplement: Supplementary file 1 [file mmc1.pdf]
